# Hierarchical, Template‐Controlled Self‐Assembly of Two Different Organometallic Capsules from a Tetrakisimidazolium Salt

**DOI:** 10.1002/anie.202520814

**Published:** 2025-11-13

**Authors:** Yang Li, Xue‐Ting Chang, Le Zhang, Guozan Yuan, Franz Ekkehardt Hahn, Ying‐Feng Han

**Affiliations:** ^1^ Key Laboratory of Synthetic and Natural Functional Molecule of the Ministry of Education, Xi'an Key Laboratory of Functional Supramolecular Structure and Materials, College of Chemistry and Materials Science Northwest University Xi'an 710127 China; ^2^ School of Chemistry and Chemical Engineering Anhui University of Technology Ma'anshan 243000 China; ^3^ Institute für Anorganische und Analytische Chemie Universität Münster Corrensstrasse 30 48149 Münster Germany

**Keywords:** Calix[4]resorcinarene, Hierarchical template, N‐heterocyclic carbene, Organometallic capsule, Structural transformation

## Abstract

Controlling the assembly of molecular nanocapsules through metal‐carbon bonds constitutes a major challenge in the field of supramolecular organometallic chemistry. Here, we demonstrate the construction of two nanosized dodecanuclear organometallic capsules, [{Ag(CH_3_CN)_4_(BF_4_)_8_}⊂Ag_12_(**1**)_6_](BF_4_)_5_ and [(CF_3_SO_3_)_8_⊂Ag_12_(**1**)_6_](CF_3_SO_3_)_4_ from the calix[4]resorcinarene‐based tetrakisimidazolium salts H_4_‐**1**(X)_4_ and Ag^I^ ions employing the metal‐carbene directed self‐assembly and hierarchical template strategies. An [Ag(CH_3_CH)_4_(BF_4_)_8_]^7−^ anion or eight triflate anions served as templates. The dodecanuclear capsules are the largest poly‐NHC‐derived assemblies known so far. Salt H_4_‐**1**(SbF_6_)_4_ reacts with Ag_2_O to give the tetranuclear assembly [(SbF_6_)⊂Ag_4_(**1**)_2_](SbF_6_)_3_ featuring an encapsulated SbF_6_^−^ anion. Transmetalation of the tetra‐NHC ligands from silver to gold yielded in all cases tetranuclear assemblies of type [Au_4_(**1**)_2_]X_4_.

## Graphical abstract

Two dodecasilver capsules were synthesized from tetra‐NHC‐decorated calix[4]resorcinarene ligands and Ag_2_O through hierarchical template‐directed self‐assembly utilizing {Ag(NCCH_3_)_4_(BF_4_)_8_}^7−^ or (CF_3_SO_3_)_8_^8−^ templates. The dodecanuclear assemblies represent the largest organometallic capsules obtained to date from poly‐NHC ligands.

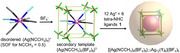

## Introduction

Rational control over the self‐assembly of metallosupramolecular architectures from small building blocks has become an attractive but challenging research topic involving various strategies.^[^ ^1^, ^2^, ^3^, ^4^, ^5^, ^6^, ^7^, ^8^, ^9^, ^10^, ^11^, ^12^, ^13^, ^14^, ^15^ ^]^ Among these, the template‐controlled assembly has become a powerful tool allowing the controlled construction of complex supramolecular architectures. For example, Sauvage et al. demonstrated more than 40 years ago that copper(I) cations control the pre‐assembly of selected building blocks for the subsequent formation of catenanes.^[^ ^16^, ^17^ ^]^ To ensure the directionality of self‐assembly and obtain the desired topological structure, the template strategy requires a perfect match in shape and size between the final product and the template. During the assembly process, metal‐ligand directed coordination bonds and other interactions, such as hydrogen bonds, van der Waals forces, π···π stacking, and CH···π interactions, collectively contribute to the formation of the targeted assemblies. This strategy has become an important and likely the only means to construct complex coordination systems.^[^ ^18^, ^19^, ^20^, ^21^, ^22^, ^23^, ^24^, ^25^, ^26^, ^27^, ^28^, ^29^, ^30^, ^31^, ^32^ ^]^

In nature, the formation of life‐related complex functional structures through spontaneous assembly with high geometric precision is often aided by small molecule templates. This hierarchical self‐assembly strategy is based on the combination of molecular units into secondary structures via non‐covalent interactions, which subsequently serve as templates for the formation of more complex superstructures.^[^ ^33^, ^34^, ^35^, ^36^, ^37^ ^]^

Werner‐type ligands have been widely used as subcomponents for the generation of supramolecular assemblies, including various nanocapsule structures.^[^ ^38^, ^39^, ^40^, ^41^, ^42^ ^]^ In contrast, N‐heterocyclic carbene (NHC) ligands, featuring carbon donors, have been employed much less for the synthesis of nanocasules,^[^ ^43^, ^44^, ^45^, ^46^, ^47^, ^48^, ^49^, ^50^, ^51^, ^52^ ^]^ although NHCs have shown great promise in the construction of supramolecular organometallic structures.^[^ ^53^, ^54^, ^55^, ^56^, ^57^, ^58^ ^]^

The synthesis of poly‐NHC Ag^I^ assemblies from suitable polyazolium precursors and Ag_2_O has been investigated not only because of the simplicity of the synthetic procedure but also because of the lability of Ag^I^─C_NHC_ bonds, allowing the formation of the thermodynamically most stable assembly. In addition, metallosupramolecular assemblies based on Ag─C_NHC_ bonds can serve as NHC transfer agents, allowing for assemblies containing other metals.^[^ ^43^, ^46^, ^50^, ^58^ ^]^

Based on the fixed and nearly linear coordination mode observed in the C_NHC_─Ag─C_NHC_ moiety, most reported metallosupramolecular assemblies obtained from polycarbene ligands are sandwich‐type structures where metal ions are sandwiched in between two essentially planar poly‐NHC ligands.^[^ ^43^, ^44^, ^46^, ^50^ ^]^ The construction of organometallic architectures with large inner cavities is less explored and would require the use of new types of linkers between the NHC donors forming the poly‐NHCs.

Calix[4]resorcinarene is a bowl‐shaped macrocyclic cavitand with a hydrophobic cavity and terminal polar phenolic groups. It served as a building block for various self‐assembled capsules, which can act as hosts for guest molecules.^[^ ^59^, ^60^, ^61^ ^]^ A capsule composed of six calix[4]‐resorcinarene and eight water molecules held together by 60 hydrogen bonds has been described by Atwood and MacGillivray.^[^ ^62^ ^]^ Later, tetradonor‐functionalized calix[4]resorcinarene cavitands have been prepared and reacted with metal ions to give nanocapsules.^[^ ^63^, ^64^, ^65^, ^66^, ^67^, ^68^, ^69^, ^70^ ^]^ In addition, tetrakisaldehyde‐substituted calix[4]resorcinarene cavitands have been covalently linked by Schiff‐base condensation with a diamine, forming a large organic capsule.^[^ ^71^ ^]^ Most of the capsules obtained from functionalized calix[4]‐resorcinarene are composed of four cavitand molecules. While some capsules composed of six functionalized calix[4]resorcinarene units have also been reported,^[^ ^60^, ^64^, ^66^, ^68^, ^69^ ^]^ their synthesis becomes increasingly difficult as the number of components increases. To date, only a few complexes bearing NHC‐functionalized calix[4]resorcinarene are known,^[^ ^72^, ^73^ ^]^ and assemblies with such ligands have not yet been described.

**Scheme 1:**
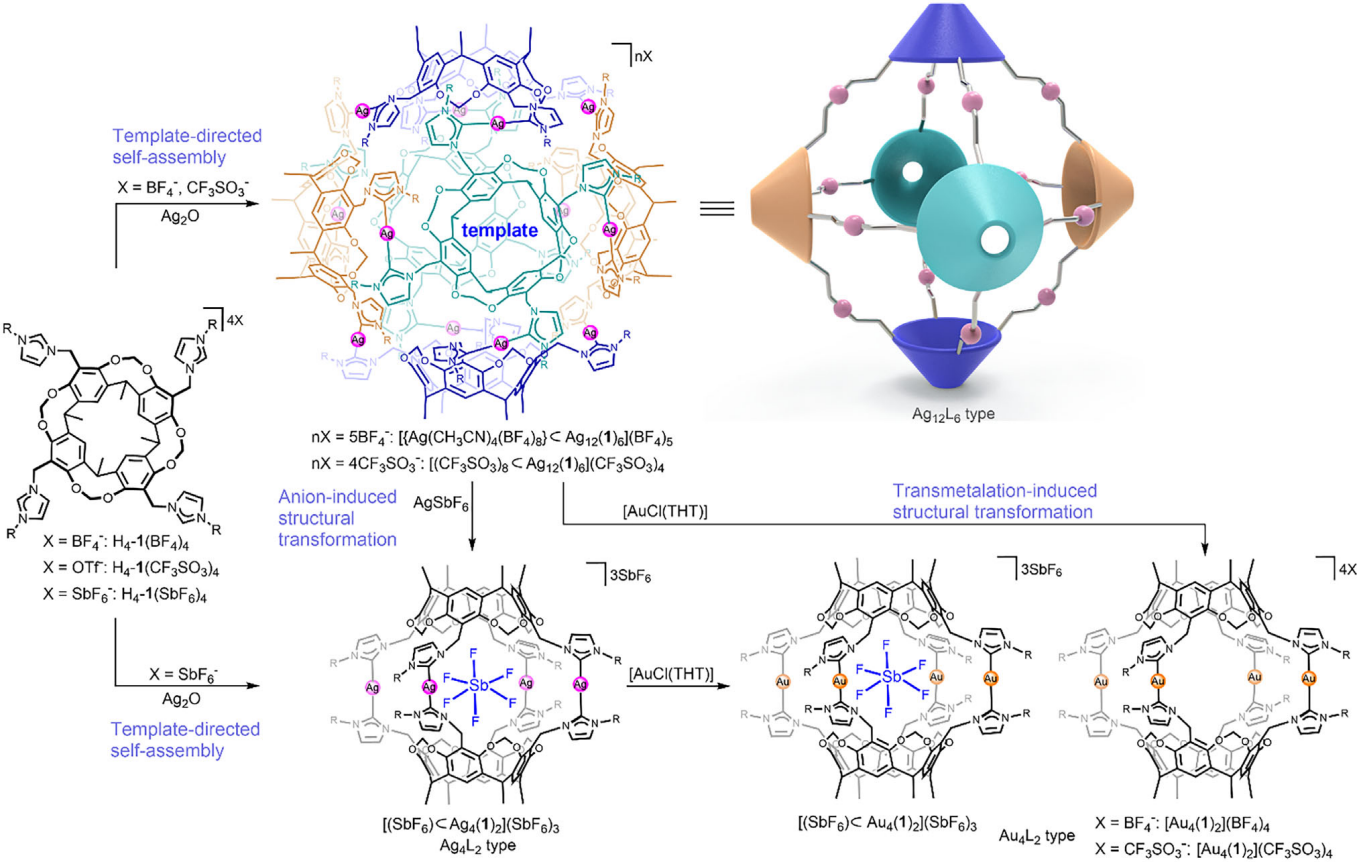
Formation of the organometallic assemblies [{Ag(NCCH_3_)_4_(BF_4_)_8_}⊃Ag_12_(**1**)_6_](BF_4_)_5_, [(CF_3_SO_3_)_8_⊃Ag_12_(**1**)_6_](CF_3_SO_3_)_4_, and [(SbF_6_)⊃Ag_4_(**1**)_2_](SbF_6_)_3_ and their structural transformations.

Herein, we report the synthesis of two nanosized organometallic capsules built from six tetrakis‐NHC‐substituted calix[4]resorcinarene molecules **1** and twelve silver(I) ions (Scheme 1). A disordered [Ag(NCCH_3_)_4_(BF_4_)_8_]^7−^ trianion or eight CF_3_SO_3_^−^ anions forming hydrogen bonds to the calix[4]resorcinarene were found as templates inside the dodecanuclear capsules. A tetranuclear capsule [(SbF_6_)⊃Ag_4_(**1**)_2_](SbF_6_)_3_ containing an SbF_6_^−^ template was obtained from H_4_‐**1**(SbF_6_)_2_ and Ag_2_O. Transmetalation reactions of the dodenuclear or tetranuclear silver capsules with [AuCl(THT)] led in all cases to the tetranuclear capsules [(SbF_6_)⊃Au_4_(**1**)_2_](SbF_6_)_3_ and [Au_4_(**1**)_2_](X)_4_ (X = BF_4_^−^, CF_3_SO_3_^−^).

## Results and Discussion

The three tetrakisimidazolium salts H_4_‐**1**(BF_4_)_4_‒H_4_‐**1**(SbF_6_)_4_ were prepared starting with the reaction of commercially available 2‐methylresocinol with paraldehyde followed by the reaction with CH_2_ClBr to give the methylene‐bridged calix[4]resorcinarene **3** (Scheme S1). Tetrabromination of **3** with N‐bromosuccinimide yielded **4**, which was subsequently reacted with *n*‐butylimidazole to give the tetrakisimidazolium salt H_4_‐**1**(Br)_4_. This salt was converted by anion exchange into the tetrafluoroborate (BF_4_^−^), trifluoromethanesulfonate (CF_3_SO_3_^−^) or hexafluoro‐antimonate (SbF_6_^−^) salts H_4_‐**1**(X)_4_ (X = BF_4_^−^, CF_3_SO_3_^−^, SbF_6_^−^). These salts and their precursors **2**‒**4** were fully characterized by ^1^H and ^13^C NMR spectroscopy and by ESI TOF mass spectroscopy (Figures S1‒S15). An X‐ray diffraction study with crystals of composition H_4_‐**1**(BF_4_)_4_·2CH_3_CN confirmed the bowl‐type molecular structure of cation **1** ^4+^ (Table S2 and Figure S58).^[^ ^74^ ^]^

The reaction of salt H_4_‐**1**(BF_4_)_4_ or H_4_‐**1**(CF_3_SO_3_)_4_ with Ag_2_O in CH_3_CN at 80 °C for 18 h resulted in the formation of two dodecanuclear capsule‐type assemblies, [{Ag(NCCH_3_)_4_(BF_4_)_8_}⊃Ag_12_(**1**)_6_](BF_4_)_5_ (yield 88%) and [(CF_3_SO_3_)_8_⊃Ag_12_(**1**)_6_](CF_3_SO_3_)_4_ (yield 83%) (Schemes 1 and S2). Both assemblies were characterized by ^1^H and ^19^F NMR spectroscopy and by ESI‐TOF mass spectrometry. The poor solubility of [{Ag(NCCH_3_)_4_(BF_4_)_8_}⊃Ag_12_(**1**)_6_](BF_4_)_5_ prevented further characterization by ^13^C NMR spectroscopy. However, the ^19^F NMR spectrum (Figure S18) featured two resonances indicating the presence of two types of BF_4_^−^ anions, most likely due to encapsulated and non‐encapsulated BF_4_^−^ anions. The ^1^H DOSY spectrum showed that all peaks in the range of *δ* = 4.0 to 8.0 ppm displayed a single diffusion constant, indicating the formation of a single species in solution (Figure S17).

**Figure 1:**
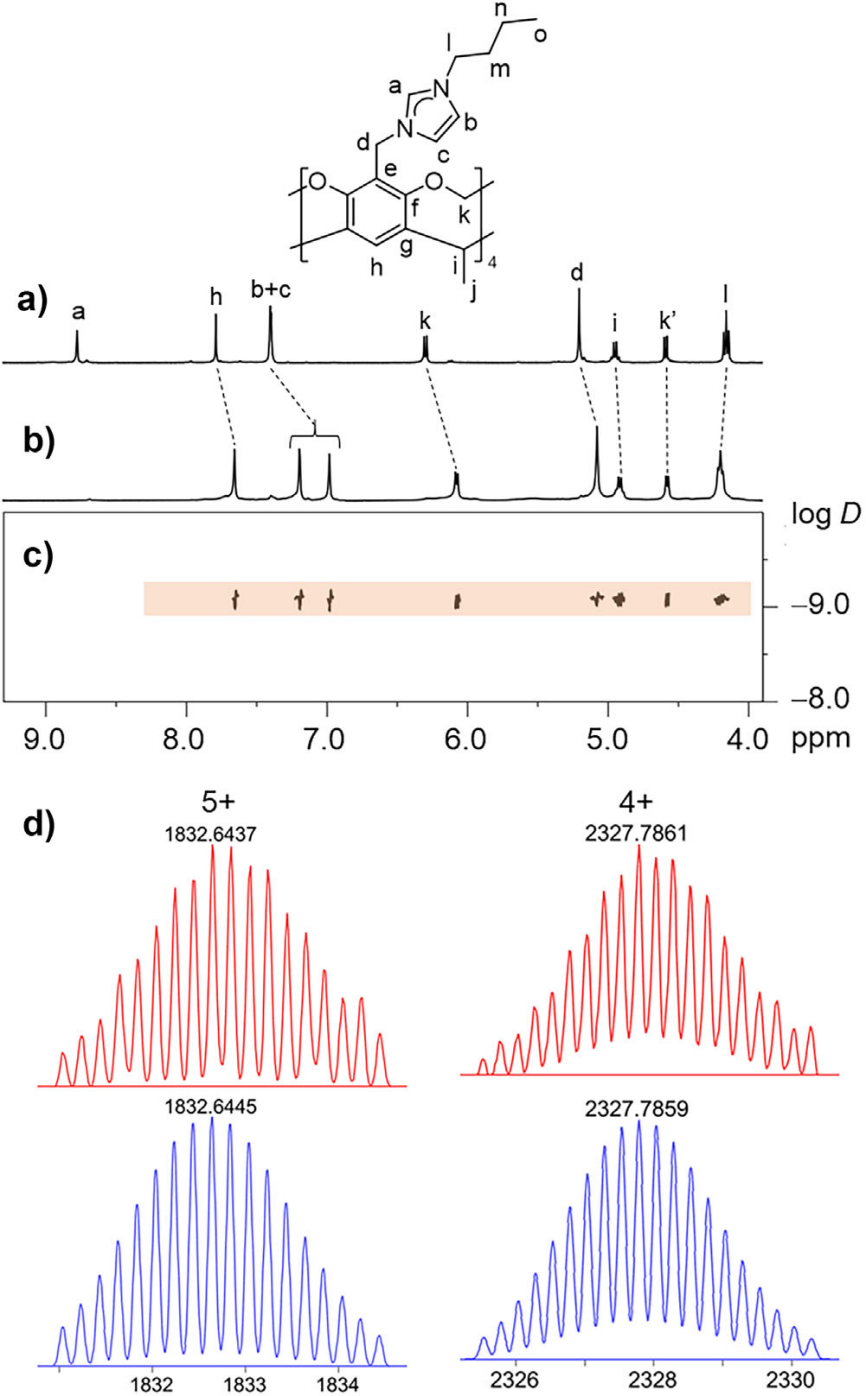
Partial ^1^H NMR spectra (400 MHz, CD_3_CN) of a) the ligand H_4_‐**1**(CF_3_SO_3_)_4_ and b) assembly [(CF_3_SO_3_)_8_⊃Ag_12_(**1**)_6_](CF_3_SO_3_)_4_. c) ^1^H DOSY spectrum of [(CF_3_SO_3_)_8_⊃Ag_12_(**1**)_6_](CF_3_SO_3_)_4_. d) ESI‐TOF mass spectrum of [(CF_3_SO_3_)_8_⊃Ag_12_(**1**)_6_](CF_3_SO_3_)_4_ (experimental in red, calculated in blue).

The ^1^H NMR spectrum of [(CF_3_SO_3_)_8_⊃Ag_12_(**1**)_6_](CF_3_SO_3_)_4_ (Figures 1b and S20) revealed the absence of the resonance for the imidazolium proton H_a_, previously observed for the tetrakisimidazolium salt at *δ* = 8.74 ppm (Figures 1a and S10). This observation, together with the detection of a typical low‐field C_NHC_ resonance^[^ ^54^, ^55^, ^56^ ^]^ at *δ* = 180.3 ppm (from HBMC spectroscopy, Figure S25), indicated the formation of an Ag(I)−‒NHC complex. DOSY spectroscopy confirmed the formation of a single product with a single vertical trace at a diffusion coefficient *D* = 8.46 × 10^−10^ m^2^·S^−1^ (log *D* = –9.07) (Figure 1c). The ^19^F NMR spectrum in CD_3_CN revealed two signals at *δ* = −79.25 and −79.92 ppm (Figure S22), indicating two different environments for the CF_3_SO_3_^−^ anions in solution. The composition of the [(CF_3_SO_3_)_8_⊃Ag_12_(**1**)_6_](CF_3_SO_3_)_4_ assembly was further confirmed by ESI‐TOF mass spectrometry with two prominent peaks observed at *m/z* = 2327.7861 (calcd for [Ag_12_(**1**)_6_(CF_3_SO_3_)_8_]^4+^ 2327.7859) and 1832.6437 (calcd for [Ag_12_(**1**)_6_(CF_3_SO_3_)_7_]^5+^ 1832.6445, Figures 1d and S26), indicating the formation of an Ag_12_(**1**)_6_ assembly.

**Figure 2:**
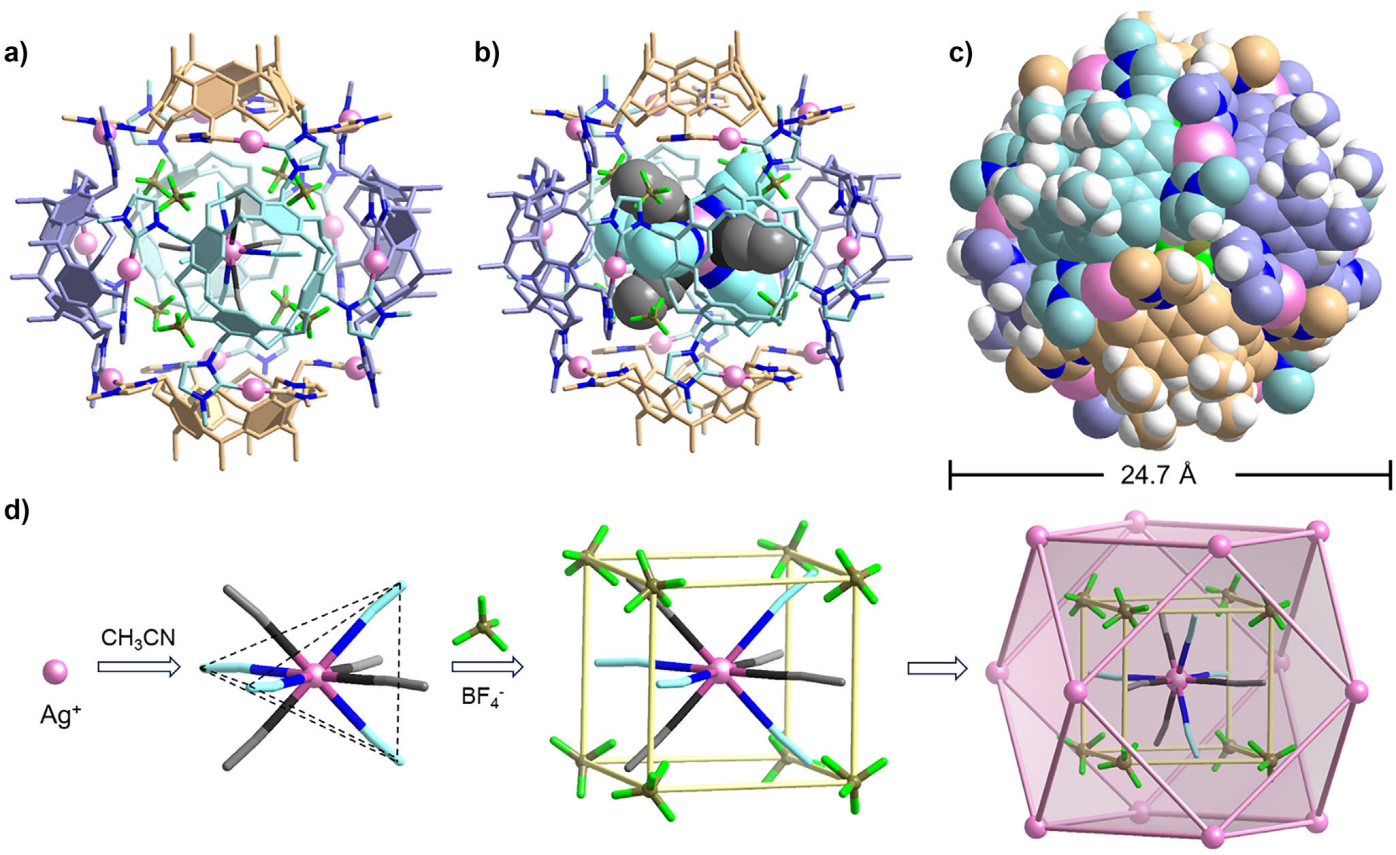
a), b) Two drawings of the [{Ag(CH_3_CN)_4_(BF_4_)_8_}⊃Ag_12_(**1**)_6_]^5+^ cation in [{Ag(CH_3_CN)_4_(BF_4_)_8_}⊃‐Ag_12_(**1**)_6_](BF_4_)_5_]·88CH_3_CN. c) space‐filling drawing of the [{Ag(CH_3_CN)_4_(BF_4_)_8_}⊃Ag_12_(**1**)_6_]^5+^ cation. The carbon atoms of the ligands are displayed in different colors (azure, orange, and gray) to differentiate the six ligands within the structure in a)–c). Hydrogen atoms have been omitted for clarity, and only the first atom of each N‐substituent is shown. d) Depiction of the internal {Ag(CH_3_CN)_4_(BF_4_)_8_}^7−^ template inside the [Ag_12_(**1**)_6_]^12+^ assembly. The four CH_3_CN ligands of the primary [Ag(CH_3_CN)_4_]^+^ template are disordered over two positions (SOF = 0.5; each tetrahedral set of ligands is depicted in a different color, blue and gray). Eight BF_4_^−^ anions also occupy the inner void of the capsule connected via B─F···H hydrogen bonds to the acetonitrile molecules.

The topology and composition of the dodecanuclear assemblies were finally determined using X‐ray diffraction analysis. These analyses revealed that the crystals of the assembly containing BF_4_^−^ anions have the actual composition [{Ag(NCCH_3_)_4_(BF_4_)_8_}⊃Ag_12_(**1**)_6_](BF_4_)_5_·88CH_3_CN (Figure 2)^[^ ^74^ ^]^ while the crystals of the assembly containing CF_3_SO_3_^−^ anions have the composition [(CF_3_SO_3_)_8_⊃Ag_12_(**1**)_6_(CF_3_SO_3_)_4_] (Figure 4).^[^ ^74^ ^]^

The assembly containing BF_4_^−^ anions is a capsule‐type structure composed of twelve silver ions and six calix[4]resorcinarene‐bridged tetra‐NHC ligands. Contrary to the situation in previously described related polycarbene‐derived assemblies,^[^ ^43^, ^44^, ^45^, ^46^, ^47^, ^48^, ^49^, ^50^ ^]^ the C_NHC_─Ag─C_NHC_ vectors are not oriented perpendicular to the plane of the tetradentate ligand but are rather nearly parallel to the outer surface of the capsule (Figure 2). Each calix[4]resorcinarene unit is connected to four adjacent calix[4]resorcinarene units through four C_NHC_─Ag─C_NHC_ links (Figure 2a). The twelve carbene‐coordinated metal centers are located at the corners of a truncated cube. The structure is composed of six identical cavitands and has a void space within the discrete capsule framework. The metric parameters found for the [Ag_12_(**1**)_6_]^12+^ cation [Ag–C_NHC_, 2.051(7)–2.064(10) Å, C_NHC_─Ag─C_NHC_, 171.0(3)–174.2(4)°] are in good agreement with the equivalent values previously described for linear Ag^I^(NHC)_2_ complexes.^[^ ^43^, ^44^, ^45^, ^46^, ^47^, ^48^, ^49^, ^50^ ^]^

**Figure 3:**
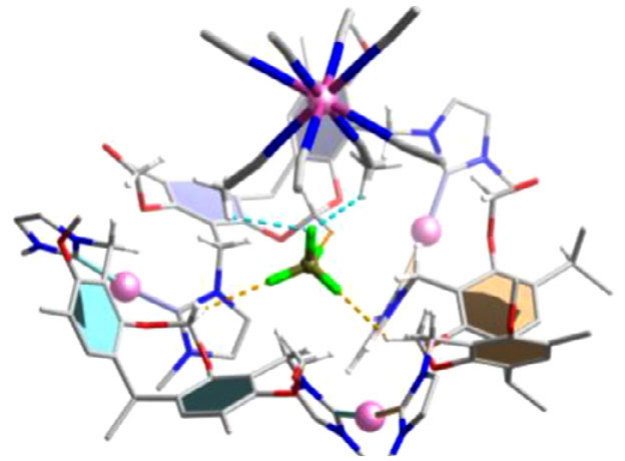
Hydrogen bonds between one of the internal BF_4_^−^ anions and two acetonitrile (in light blue) and the O─CH_2_─O (in yellow) protons of three different calix[4]resorcinarene units. Only the shortest hydrogen bonds are depicted.

The crystallographic analysis revealed that a disordered [Ag(CH_3_CN)_4_]^+^ cation is encapsulated within the cavity. Tetracoordinated Ag^+^ cations are rare but not uncommon.^[^ ^75^, ^76^ ^]^ The site occupation factors are 0.125 for Ag^+^ and 0.5 for CH_3_CN, leading, together with the given site symmetry, to a cube‐type arrangement of eight acetonitrile ligands around the Ag^+^ cation (Figure 2d). This cation serves as a primary cation template. Eight BF_4_^−^ anions are arranged around the disordered [Ag(CH_3_CN)_4_]^+^ cation. These internal BF_4_^−^ anions are each located in between two acetonitrile molecules (with SOF = 0.5), forming a slightly twisted cube (Figure 2d), thereby completing a hierarchical template. The internal BF_4_^−^ anions utilize one fluorine atom to maintain two B─F···H hydrogen bonds to the two next adjacent acetonitrile molecules (B─F···H─CH_2_ 2.396 and 2.577 Å, Figure 3). The other three fluorine atoms are engaged in B─F···H─CH hydrogen bonds to the protons of the O─CH_2_─O bridges of three different calix[4]resorcinarene units (BF···H─CH_2_ range 2.330‒2.477 Å, Figure 3). The B─F···H hydrogen bonds of the internal BF_4_^−^ anions are the reason for the observation of two resonances in the ^19^F NMR spectrum (Figure S18). The internal [Ag(NCCH_3_)_4_(BF_4_)_8_]^7−^ moiety acts as a hierarchical template for the formation and stabilization of the capsule structure of [{Ag(CH_3_CN)_4_(BF_4_)_8_}⊃Ag_12_(**1**)_6_](BF_4_)_5_].

**Figure 4:**
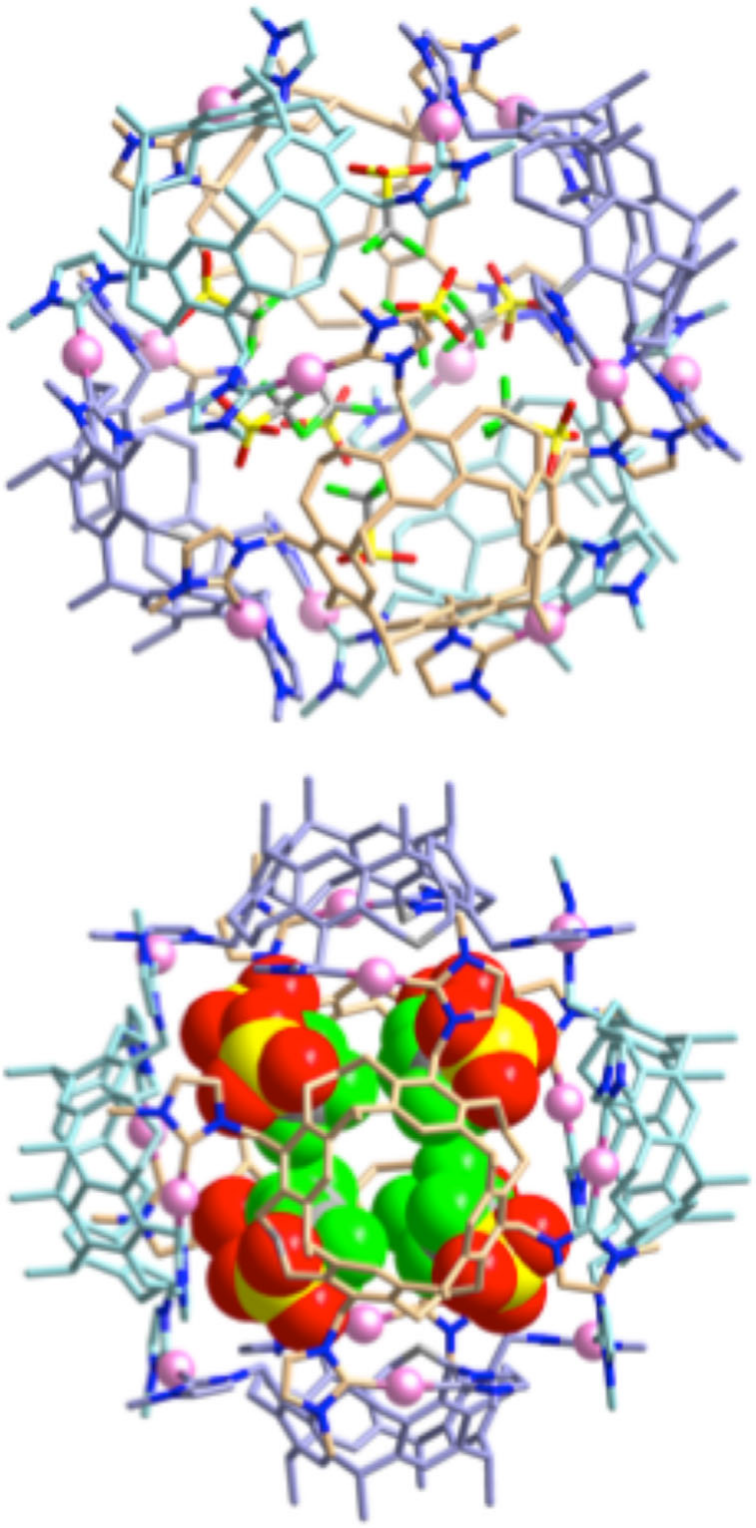
Molecular structure of the [(CF_3_SO_3_)_8_⊃Ag_12_(**1**)_6_]^4+^ cation in [(CF_3_SO_3_)_8_⊃Ag_12_(**1**)_6_](CF_3_SO_3_)_4_·54CH_3_CN. Top: ball and stick representation; bottom: depiction of the assembly with eight space‐filling encapsulated CF_3_SO_3_^−^ anions pointing with the SO_3_ groups toward the walls of the capsule. Hydrogen atoms have been omitted for clarity, and only the first atom of the N‐butyl substituents is shown.

A related situation was found for the molecular structure of the triflate salt [Ag_12_(**1**)_6_](CF_3_SO_3_)_12_. Again, an Ag_12_ **1**_6_ capsule was formed, this time starting from H_4_‐**1**(CF_3_SO_3_)_4_ and Ag_2_O. Eight of the twelve triflate anions are encapsulated. Thus, the structure is best described as [(CF_3_SO_3_)_8_⊃Ag_12_(**1**)_6_]‐(CF_3_SO_3_)_4_ (Figure 4). The SO_3_ groups of all CF_3_SO_3_^−^ anions point toward the inner walls of the capsule, where they are engaged in S─O···H hydrogen bonds. Each SO_3_ group maintains three hydrogen bonds to the O─CH_2_─O groups of three different calix[4]resorcinarene units in the range of 2.325‒2.640 Å. Clearly, the eight CF_3_SO_3_^−^ anions act as templates for the formation and stabilization of the assembly. The four triflate anions outside the capsule also form S─O···H hydrogen bonds to imidazole or phenyl protons. In addition, the asymmetric unit contains a total of 54 disordered acetonitrile molecules.

A different picture emerges if the tetrakisimidazolium salt H_4_‐**1**(SbF_6_)_4_ is reacted with Ag_2_O. This salt differs from salts H_4_‐**1**(BF_4_)_4_ and H_4_‐**1**(CF_3_SO_3_)_4_ only in the choice of the anion. The reaction of H_4_‐**1**(SbF_6_)_4_ with Ag_2_O yields exclusively the tetranuclear capsule [Ag_4_(**1**)_2_](SbF_6_)_4_ in 92% yield (Scheme 1). The formation of [Ag_4_(**1**)_2_](SbF_6_)_4_ was initially indicated by ESI‐TOF mass spectrometry showing prominent peaks at *m/z* = 980.5311 (calcd for [Ag_4_(**1**)_2_SbF_6_]^3+^ 980.5788) and 676.6748 (calcd for [Ag_4_(**1**)_2_]^4+^ 676.7109, Figure S33). Multinuclear NMR spectroscopy also indicated the formation of a carbene complex (Figures S27‒S32). The ^1^H‐^13^C HMBC spectrum (Figure S32) revealed the resonance for the C_NHC_ atom as a doublet at *δ* = 180.1 (^1^ *J*(C‐Ag^107/109^) = 212.4 Hz, Figure S32). This value falls in the expected range for linear C_NHC_─Ag─C_NHC_ complexes.^[^ ^54^, ^55^, ^56^ ^]^

**Figure 5:**
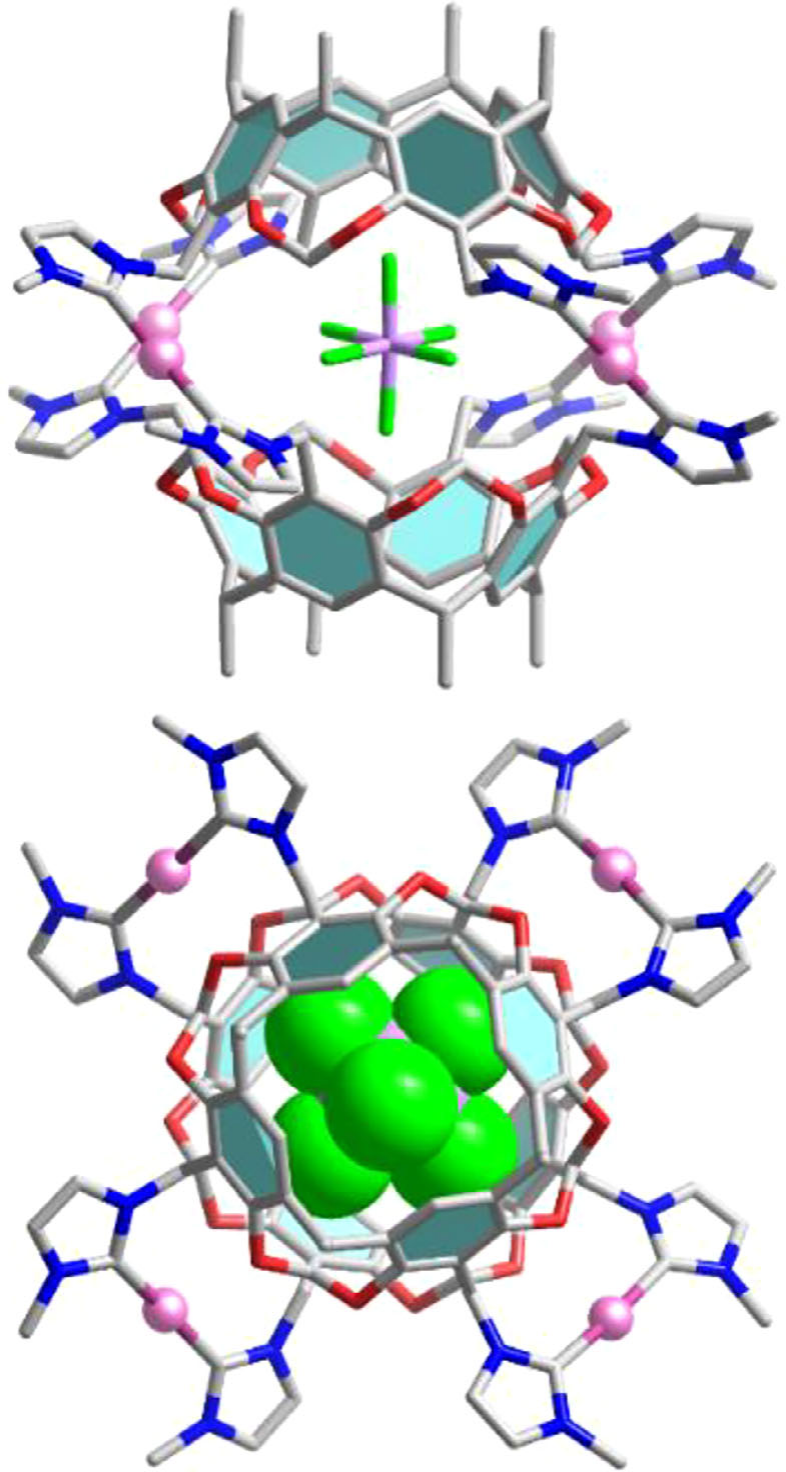
Molecular structure of the [(SbF_6_)⊃Ag_4_(**1**)_2_]^3+^ cation in [(SbF_6_)⊃Ag_4_(**1**)_2_](SbF_6_)_3_·2CH_3_CN (only the first atom of each N‐butyl substituent is shown). Top: side view (ball and stick representation); bottom: top view (with space‐filling representation of the SbF_6_^−^ anion).

Unequivocal evidence for the formation of a tetrasilver assembly was provided by an X‐ray diffraction study with crystals of composition [Ag_4_(**1**)_2_](SbF_6_)_4_·2CH_3_CN.^[^ ^74^ ^]^ The X‐ray diffraction analysis revealed a capsule‐type structure composed of four silver atoms sandwiched in between two tetracarbene ligands **1**. One of the four SbF_6_‒ anions is encapsulated inside the Ag_4_ **1**_2_ assembly (Figure 5).

The molecular structure is thus best described as [(SbF_6_)⊃Ag_4_(**1**)_2_](SbF_6_)_3_. The [(SbF_6_)⊃Ag_4_(**1**)_2_]^3+^ cation resides on a crystallographic twofold axis bisecting two F─Sb─F planes. The four fluorine atoms of these planes are engaged in Sb─F···HCH hydrogen bonds with the bridging O─CH_2_─O groups of the tetracarbene ligands. Each fluorine atom forms two hydrogen bonds (range 2.58–2.70 Å) connecting the two tetracarbene ligands via H···F···H hydrogen bonds (Figure S59), thereby stabilizing the whole assembly. The ^19^F NMR spectrum (Figure S29) proved non‐informative regarding the encapsulation of the SbF_6_^−^ anion. It featured multiple signals in the range of *δ* = 110‒138 ppm. Multiple peaks are expected due to coupling of the fluorine atoms to ^121^Sb and ^123^Sb.^[^ ^77^ ^]^ However, due to possible overlap of the resonances of encapsulated and non‐encapsulated SbF_6_^−^ anions, assignment of the resonances was not possible.

The hydrodynamic radii of the dodecanuclear capsules were determined as 11.2 Å for cation [(CF_3_SO_3_)_8_⊃Ag_12_(**1**)_6_]^4+^ (Figure S60) and 10.9 Å for [{Ag(CH_3_CN)_4_(BF_4_)_8_}⊃Ag_12_(**1**)_6_]^5+^ (Figure S61), while the hydrodynamic radius for the tetranuclear capsule cation [(SbF_6_)Ag_4_(**1**)_2_]^3+^ was calculated to measure 6.13 Å (Figure S63). The same trend was observed for the calculation of the internal cavity volumes after removal of the templates. For the dodecanuclear capsules, volumes of 1674.8 and 1614.6 Å^3^ were calculated, while the tetranuclear capsule features, as expected, a much smaller internal cavity volume of only 204.9 Å^3^ (Figures S64‒S66).

After the isolation of two types of capsules from the same tetracarbene ligand but depending on the anion present, we examined the interconversion of these assemblies upon addition of selected anions. In a first experiment, compound [(SbF_6_)⊃Ag_4_(**1**)_2_](SbF_6_)_3_ was treated with an excess of AgBF_4_, expecting that the corresponding dodecanuclear assembly would form. However, no conversion was observed, and the ESI‐TOF mass spectrum of the reaction mixture only exhibited signals for [Ag_4_(**1**)_2_(SbF_6_)_4_ (Figures S33 and S34).

However, the addition of AgSbF_6_ to an acetonitrile solution of assembly [(CF_3_SO_3_)_8_⊃Ag_12_(**1**)_6_](CF_3_SO_3_)_4_ and stirring of the mixture for 3 h resulted in full conversion of the dodecanuclear to the tetranuclear structure [(SbF_6_)⊃Ag_4_(**1**)_2_](SbF_6_)_3_. This transformation was monitored by ESI‐TOF mass spectrometry (Figure S35). Two strong peaks observed at *m/z* = 1588.3543 and 980.6209 were assigned to the ions [Ag_4_(**1**)_2_(SbF_6_)_2_]^2+^ and [Ag_4_(**1**)_2_(SbF_6_)]^3+^ respectively. No peaks assignable to the dodecanuclear capsule were detected anymore. Based on the molecular structure of cation [(SbF_6_)⊃Ag_4_(**1**)_2_]^3+^ in [(SbF_6_)⊃Ag_4_(**1**)_2_](SbF_6_)_3_⋅2CH_3_CN (Figure 5), the interaction of the encapsulated SbF_6_^−^ anion with the two tetracarbene ligands likely facilitated the observed transformation from the dodenuclear to the tetranuclear capsule. Similar observations were made if AgSbF_6_ was added to an acetonitrile solution of [{Ag(CH_3_CN)_4_(BF_4_)_8_}**⊃**Ag_12_(**1**)_6_](BF_4_)_5_ (Figure S36). These experiments demonstrate the importance of (hierarchical) templates for the formation of dodecanuclear and tetranuclear organometallic capsules. Apparently, the shape of the SbF_6_^−^ anion induced the formation of the tetranuclear assembly while also featuring a stronger template effect.

Due to the labile nature of the Ag─C_NHC_ bond, silver‐NHC complexes have been established as excellent carbene transfer agents, allowing the synthesis of other NHC‐derived organometallic structures.^[^ ^45^, ^46^, ^47^, ^48^, ^49^, ^50^, ^51^ ^]^ It was therefore attempted to substitute the silver atoms in the dodecanuclear and tetranuclear assemblies for gold atoms while maintaining the overall geometry of the assemblies.

Stirring acetonitrile solutions of the dodecanuclar capsules [{Ag(CH_3_CN)_4_(BF_4_)_8_}⊂Ag_12_(**1**)_6_](BF_4_)_5_ or [(CF_3_SO_3_)_8_‐ ⊂Ag_12_(**1**)_6_](CF_3_SO_3_)_4_ with an excess of [AuCl(THT)] (THT = tetrahydrothiophene) at ambient temperature for 24 h yielded exclusively the tetranuclear gold(I) assemblies [Au_4_(**1**)_2_](BF_4_)_4_ and [Au_4_(**1**)_2_](CF_3_SO_3_)_4_ in 87% and 96% yield, respectively (Schemes 1 and S3). The formation of the two tetranuclear assemblies was first indicated by ESI‐TOF mass spectrometry showing the strongest peaks at *m/z* = 765.5321 (calcd for [Au_4_(**1**)_2_]^4+^ 765.5217) and 1049.7183 (calcd for [Au_4_(**1**)_2_(BF_4_]^3+^ 1049.6972, Figure S43) and at *m/z* = 1070.4192 (calcd for [Au_4_(**1**)_2_(CF_3_SO_3_)]^3+^ 1070.3465, Figure S50), respectively. The ^13^C NMR spectra feature the C_NHC_ resonance in the typical range for Au(NHC)_2_ complexes at *δ* = 184.1 ppm (for [Au_4_(**1**)_2_](BF_4_)_4_, Figure S38) and at *δ* = 184.0 ppm (for [Au_4_(**1**)_2_](CF_3_SO_3_)_4_, Figure S45). Most importantly, only one resonance was observed for each assembly in the ^19^F NMR spectra at *δ* = 151.61 ppm (for [Au_4_(**1**)_2_](BF_4_)_4_, Figure S39) and at *δ* = 79.78 ppm (for [Au_4_(**1**)_2_](CF_3_SO_3_)_4_, Figure S46).

In addition, the conversion of the tetrasilver complex [(SbF_6_)⊃Ag_4_(**1**)_2_](SbF_6_)_3_ into the tetragold complex [(SbF_6_)⊃Au_4_(**1**)_2_](SbF_6_)_3_ through transmetalation with [AuCl(THT)] was also achieved (Scheme 1). The ESI‐TOF mass spectrum showed the strongest peaks at *m/z* = 765.5467 (calcd for [Au_4_(**1**)_2_]^4+^ 765.5217), confirming the formation of a tetranuclear assembly (Figure S57). The NMR spectra of [(SbF_6_)⊃Au_4_(**1**)_2_](SbF_6_)_3_ (Figures S51‒S56) closely resemble those of the silver analog [(SbF_6_)⊃Ag_4_(**1**)_2_](SbF_6_)_3_. We thus assumed that the gold assembly adopts a molecular structure similar to the silver derivative.

**Figure 6:**
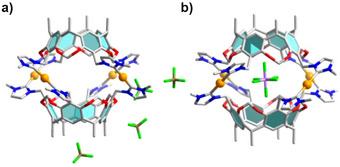
Stick representations of the cations [Au_4_(**1**)_2_]^4+^ in [Au_4_(**1**)_2_](BF_4_)_4_·11CH_3_CN a) and [(SbF_6_)⊃Au_4_(**1**)_2_]^3+^ in [(SbF_6_)⊃Au_4_(**1**)_2_](SbF_6_)_3_·5CH_3_CN⋅H_2_O b). Hydrogen atoms have been omitted for clarity, and only the first atom of each N‐substituent is shown.

Structural evidence for the Au_4_L_2_ core geometry of [Au_4_(**1**)_2_](BF_4_)_4_ and [Au_4_(**1**)_2_](SbF_6_)_4_ was provided by X‐ray crystallographic analysis.^[^ ^74^ ^]^ The molecular structure determination of [Au_4_(**1**)_2_](BF_4_)_4_·11CH_3_CN revealed a cationic cage structure [Au_4_(**1**)_2_]^4+^ composed of four gold atoms sandwiched in between two tetra‐NHC ligands **1** (Figure 6a). The four BF_4_^−^ anions reside outside the cage. The formation of the tetranuclear assembly with no encapsulated BF_4_^−^ anions was unexpected given that tetracarbene ligand **1** forms a dodecanuclear assembly [{Ag(CH_3_CN)_4_(BF_4_)_8_}⊃Ag_12_(**1**)_6_]^5+^ with silver(I) ions in the presence of BF_4_^−^ anions (Scheme 1 and Figure 2). The reason for the stability of the tetranuclear gold(I) assembly compared to the dodecanuclear silver(I) assembly must be the higher kinetic stability of the Au─C_NHC_ bond compared to the Ag─C_NHC_ bond. The tetranuclear gold(I) assembly, once formed, does not rearrange into a larger, template‐stabilized assembly based on the kinetic stability of the Au─C_NHC_ bonds and a possible shape mismatch between the BF_4_^−^ anions and the cavity.

The molecular structure determination with crystals of composition [Au_4_(**1**)_2_](SbF_6_)_4_·5CH_3_CN⋅H_2_O revealed that the molecular structure of the gold assembly (Figure 6b) is indeed isostructural to that of the silver assembly in [(SbF_6_)⊂Ag_4_(**1**)_2_](SbF_6_)_3_·2CH_3_CN (Figure 5). It is a cage structure composed of four gold atoms sandwiched in between two tetra‐NHC ligands **1**. The cage contains an encapsulated SbF_6_^−^ anion. The encapsulated SbF_6_^−^ anion stabilizes the assembly via the formation of Sb─F···H─CH interactions between the SbF_6_^−^ anion and the methylene groups of the tetra‐NHC ligand. As was observed with the silver(I) assemblies, the shape of the SbF_6_^−^ allows its encapsulation in the tetranuclear assembly, where it also features a stronger template effect than the BF_4_^−^ anions in of [Au_4_(**1**)_2_](BF_4_)_4_ or the CF_3_SO_3_^−^ anion in [Au_4_(**1**)_2_](CF_3_SO_3_)_4_.

We assumed that the tetranuclear gold assemblies formed due to the difference in the kinetic stability of Au─C_NHC_ and Ag─C_NHC_ bonds, where encapsulation of templates is not necessary as observed in the cases of [Au_4_(**1**)_2_](BF_4_)_4_ and [Au_4_(**1**)_2_](CF_3_SO_3_)_4_. The observation of an encapsulated SbF_6_^−^ anion in [(SbF_6_)⊂Au_4_(**1**)_2_](SbF_6_)_3_⋅5CH_3_CN⋅H_2_O is obviously caused by the perfect shape and size of this anion and its stronger templating effect.

The difference in the stability of Au─C_NHC_ and Ag─C_NHC_ bonds is also evident from a comparison of the ^1^H NMR spectra of [(SbF_6_)⊃Au_4_(**1**)_2_](SbF_6_)_3_ and [(SbF_6_)⊃Ag_4_(**1**)_2_](SbF_6_)_3_. The methylene protons d in these assemblies are diastereotopic, but only a broad multiplet is observed in the ^1^H NMR spectrum of the more labile cation [(SbF_6_)⊃Ag(**1**)_2_]^3+^ (Figure S27). For the kinetically more stable cation [(SbF_6_)⊃Au(**1**)_2_]^3+^ two distinct doublets are recorded for these protons at *δ* = 5.32 and 4.97 ppm (Figure S51).

## Conclusion

We describe three examples for (hierarchical) template‐directed self‐assembly. Two of these reactions lead to unprecedented organometallic dodecasilver capsules composed of six NHC‐decorated calix[4]resorcinarene ligands and twelve Ag^I^ ions. The dodecanuclear capsules obtained from tetracarbene precursors H_4_‐**1**(X)_4_ (X = BF_4_^−^, CF_3_SO_3_^−^) contained either a {Ag(NCCH_3_)_4_(BF_4_)_8_}^7−^ or a (CF_3_SO_3_)_8_^8−^ template, stabilizing the capsules via multiple CH···F or CH···O interactions between the template anions and the tetracarbene ligands. The tetra‐NHC precursor H_4_‐**1**(SbF_6_)_4_ yields in the reaction with Ag_2_O the tetranuclear assembly [(SbF_6_)⊃Ag(**1**)_2_](SbF_6_)_3_, featuring an encapsulated SbF_6_^−^ anion. All three silver assemblies undergo a transmetalation reaction with [AuCl(THT)]. For the dodecanuclear capsules this reaction leads to tetranuclear assemblies [Au_4_(**1**)_2_]X_4_ (X = BF_4_^−^, CF_3_SO_3_^−^) featuring four non‐encapsulated anions. Compound [(SbF_6_)⊃Ag(**1**)_2_](SbF_6_)_3_ also undergoes transmetalation with [AuCl(THT)] to yield [(SbF_6_)⊃Au(**1**)_2_](SbF_6_)_3_ with retention of the tetranuclear structure and an encapsulated SbF_6_^−^ anion. The structural transformation from the two dodecanuclear silver complexes to the tetranuclear gold complexes is attributed to the higher stability of the Au─C_NHC_ bonds not requiring a template to form a stable metallosupramolecular structure. This study produced the largest organometallic assemblies based on poly‐NHC ligands reported thus far and sheds light on the crucial influence that template anions can exert on the ultimately formed metallosupramolecular assembly.

## Conflict of Interests

The authors declare no conflict of interest.

## Supporting information



Supporting Information

Supporting Information

## Data Availability

The data that support the findings of this study are available in the Supporting Information of this article.
