# Supplementary material for: Hierarchical, Template‐Controlled Self‐Assembly of Two Different Organometallic Capsules from a Tetrakisimidazolium Salt
Source: Angew Chem Int Ed Engl. 2025 Nov 13;65(1):e20814. doi: 10.1002/anie.202520814 (PMC12759195; doi:10.1002/anie.202520814)
Supplement: Supplementary file 1 — Supporting Information [file ANIE-65-e20814-s001.pdf]

Table of Contents

1. Materials and methods S2
2. Synthesis of tetrakisimidazolium salts H_4-**1**$(X)_4$ ($X = BF_4^-$, OTf^-, SbF_6^-) S3
3. Synthesis of assemblies $[\{Ag(CH_3CN)_4(BF_4)_8\}\subset Ag_{12}(\mathbf{1})_6](BF_4)_5$, $[(OTf)_8\subset Ag_{12}(\mathbf{1})_6](OTf)_4$ and $[(SbF_6)\subset Ag_4(\mathbf{1})_2](SbF_6)_3$ S7
4. Synthesis of assemblies $[Au_4(\mathbf{1})_2](X)_4$ ($X = BF_4^-$, OTf^-) and of $[(SbF_6)\subset Au_4(\mathbf{1})_2](SbF_6)_3$ S10
5. Selected NMR and mass spectra of all new compounds S13
6. X-ray crystallography S42
7. Calculation of the hydrodynamic radii and the cavity volume for selected capsules S51
8. References S55

1. Materials and methods

All starting materials have been used as received from commercial sources unless stated otherwise. Solvents were freshly distilled by standard procedures before use. All reactions were carried out under a nitrogen atmosphere using standard Schlenk techniques. ^{1}H and ^{13}C{^{1}H} and 2D NMR spectra were recorded on JEOL ECZ400R, Bruker AVANCE III 400 or AVANCE III 600 spectrometers. Chemical shifts (δ) are expressed in ppm downfield from tetramethylsilane using the residual protonated solvent as an internal standard. Coupling constants are expressed in Hertz. Mass spectra were obtained with a Bruker microTOF-Q II mass spectrometer (Bruker Daltonics Corp., USA) or a Synapt G2 ESI-Q-TOF mass spectrometer in the electrospray ionization (ESI) mode. The UV-Vis experiments were conducted on an Agilent Cary-100 spectrophotometer. Compounds **2**,[1] **3**,[2] and **4**[3] were prepared using slightly modified published procedures.

2. Synthesis of tetrakismidazolium salts H_4-1(X)$_4$ (X = BF_4^-, OTf$^-$, SbF_6^-)

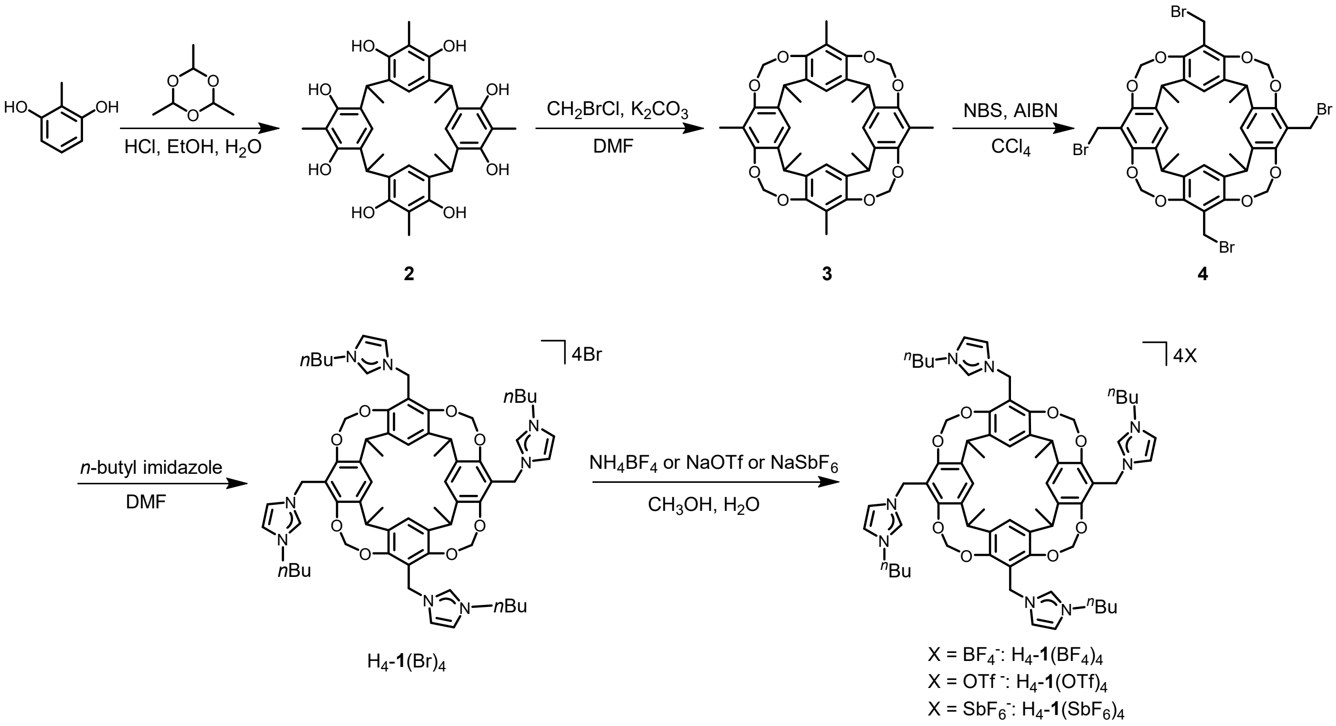

Scheme S1. General synthesis of tetrakisimidazolium salts H_4-**1**(X)$_4$ (X = BF_4^-, OTf$^-$, SbF_6^-).

2.1. Synthesis of compound 2

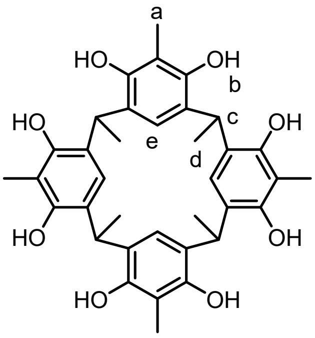

A sample of 2-methylresorcinol (10.00 g, 80.56 mmol) was dissolved in a mixture of ethanol (76 mL) and water (4 mL). To this was added paraldehyde (3.55 g, 26.86 mmol) and hydrochloric acid (37%, 18.3 mL). The reaction mixture was stirred at 70 °C for 16 h. Subsequently, the reaction mixture was cooled to ambient temperature and H_2O (200 mL) was added. The mixture was filtered and the yellow-brown solid obtained was dried *in vacuo*. Yield: 10.17 g (16.93 mmol, 84%). ^{1}H NMR (400 MHz, DMSO-d_6): δ = 8.66 (s, 8H, H_b), 7.38 (s, 4H, H_e), 4.43 (q, ^3J = 7.2 Hz, 4H, H_c), 1.94 (s, 12H, H_a), 1.70 ppm (d, ^3J = 7.2 Hz, 12H, H_d). The ^{1}H NMR spectrum of the synthesized compound **2** is consistent with the one previously reported for this compound.[1,3]

2.2. Synthesis of compound 3

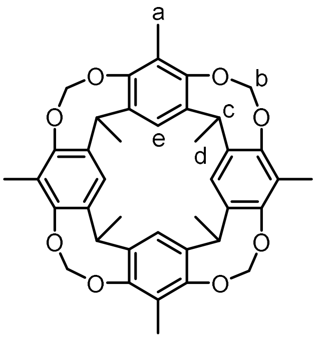

A sample of compound **2** (2.00 g, 3.33 mmol) was dissolved in *N,N*-dimethylformamide (30 mL). Subsequently, potassium carbonate (9.20 g, 66.57 mmol) was added. The mixture was stirred at ambient temperature for 1 h. Then bromochloromethane (7.18 g, 55.50 mmol) was added. The reaction mixture was stirred at 80 °C for 8 h under nitrogen. After cooling to ambient temperature, the mixture was poured into a 2% HCl solution (200 mL). The mixture was filtered and the isolated solid was dissolved in CH_2Cl_2. The mixture was again filtered and the filtrate obtained was brought to dryness. Purification by chromatography on silica gel using petroleum ether:ethyl acetate = 4:1 as eluent (500 mL) gave a colorless solid which was washed with methanol (30 mL) to give **3** as a colorless powder. Yield: 1.47 g (2.27 mmol, 68%). ^{1}H NMR (400 MHz, $CDCl_3$): δ= 7.11 (s, 4H, H_e), 5.89 (d, ^2J = 6.8 Hz, 4H, H_b), 4.99 (q, ^3J = 7.2 Hz, 4H, H_c), 4.27 (d, ^2J = 6.8 Hz, 4H, $H_{b'}$), 1.97 (s, 12H, H_a), 1.73 ppm (d, ^3J = 7.2 Hz, 12H, H_d). The ^{1}H NMR spectrum of the synthesized compound **3** was consistent with the one previously reported for this compound.[2,3]

2.3. Synthesis of compound 4

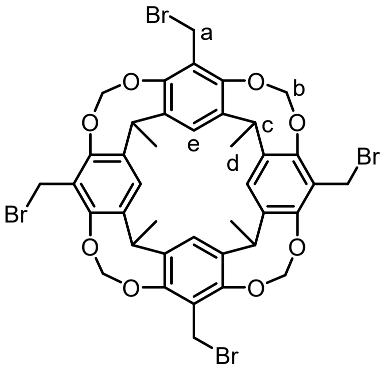

A sample of compound **3** (200.0 mg, 0.31 mmol) was dissolved in tetrachloromethane (30 mL). To this was added N-bromosuccinimide (383.0 mg, 2.15 mmol) and 2,2'-azobis(2-methylpropionitrile) (6.9 mg, 0.04 mmol) were added. The reaction mixture was stirred at 80 °C for 8 h. The reaction mixture was then cooled to ambient temperature and filtered. The filtrate was brought to dryness. Then methanol (15 mL) was added to the residue and the mixture was filtered to give **4** as a colorless powder. Yield: 213.0 mg (0.22 mmol, 71%). ^{1}H NMR (400 MHz, $CDCl_3$): δ= 7.23 (s, 4H, H_e), 6.04 (d, ^2J = 7.1 Hz, 4H, H_b), 5.02 (q, ^3J = 7.3 Hz, 4H, H_c), 4.58 (d, ^2J = 7.1 Hz, 4H, $H_{b'}$), 4.42 (s, 8H, H_a), 1.75 ppm (d, ^3J = 7.3 Hz, 12H, H_d). The ^{1}H NMR spectrum of the synthesized compound **4** is consistent with the one previously reported for this compound.[2,3]

2.4. Synthesis of compound H_4-1(Br)$_4$

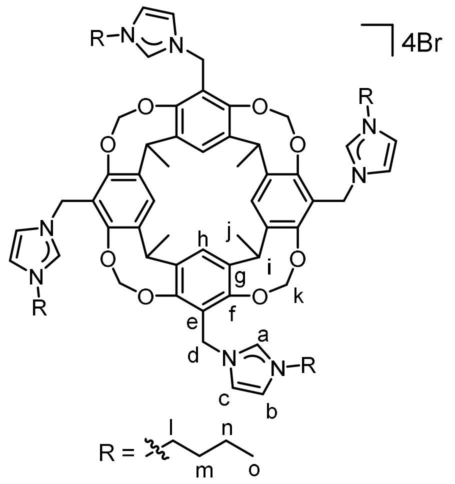

A mixture of compound **4** (200.0 mg, 0.21 mmol) and *n*-butyl imidazole (257.6 mg, 2.07 mmol) were dissolved in DMF (15 mL) in a Schlenk flask. The mixture was heated to 110 °C for 24 h. After cooling to ambient temperature, the solvent was removed *in vacuo* and the solid was washed with diethyl ether (25 mL) and dried *in vacuo* to give a white solid. Yield: 271.3 mg (0.19 mmol, 90.0%). ^{1}H NMR (400 MHz, DMSO-d_6): δ = 9.31 (s, 4H, H_a), 8.08 (s, 4H, H_h), 7.81 (s, br, 4H, H_b/H_c), 7.65 (s, br, 4H, H_b/H_c), 6.29 (d, ^2J = 7.8 Hz, 4H, H_k), 5.26 (s, 8H, H_d), 4.79 (q, ^3J = 7.0 Hz, 4H, H_i), 4.43 (d, ^2J = 7.8 Hz, 4H, $H_{k'}$), 4.20 (t, ^3J = 7.2 Hz, 8H, H_l), 1.90 (d, ^3J = 7.0 Hz, 12H, H_j), 1.80–1.72 (m, 8H, H_m), 1.30–1.21 (m, 8H, H_n), 0.90 ppm (t, ^3J = 7.4 Hz, 12H, H_o). ^{13}C{^{1}H} NMR (100 MHz, DMSO-d_6): δ = 152.3 (C_a), 139.9, 136.2, 122.7, 122.6, 120.5, 108.2 (C_b, C_c, C_e, C_f, C_g, C_h), 99.5 (C_k), 48.6 (C_l), 42.4 (C_d), 32.0 (C_i), 31.4 (C_m), 18.8 (C_n), 16.8 (C_j), 13.3 ppm (C_o). ESI-TOF MS: *m/z* = 407.1840 (calcd for [H_4-**1**(Br)]$^{3+}$ 407.1855), 285.1586 (calcd for [H_4-**1**]$^{4+}$ 285.1598).

2.5. Synthesis of compound H_4-1(BF_4)$_4$

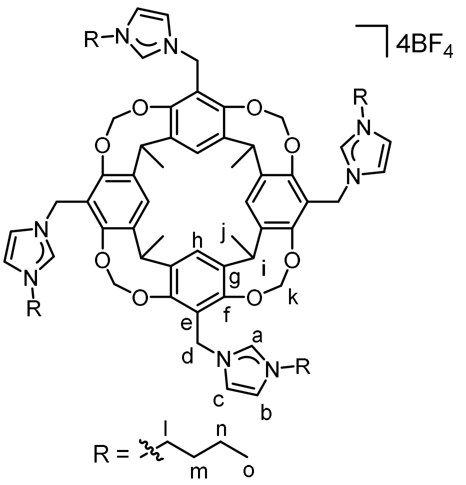

A sample of H_4-**1**(Br)$_4$ (100 mg, 0.068 mmol) was dissolved in methanol (10 mL). To this was added NH_4BF_4 (57.4 mg, 0.55 mmol) in water (15 mL). The mixture was stirred at ambient temperature for 12 h. The formed solid was collected by filtration and washed with water and diethyl ether to give compound H_4-**1**(BF_4)$_4$ as a colorless solid. Yield: 96.4 mg (0.065 mmol, 95%). ^{1}H NMR (400 MHz, CD_3CN): δ = 8.59 (s, 4H, H_a), 7.71 (s, 4H, H_h), 7.40 (d, ^3J = 1.7 Hz, 4H, H_b/H_c), 7.39 (d, ^3J = 1.7 Hz, 4H, H_b/H_c), 6.26 (d, ^2J = 7.8 Hz, 4H, H_k), 5.19 (s, 8H, H_d), 4.96 (q, ^3J = 7.7 Hz, 4H, H_i), 4.58 (d, ^2J = 7.8 Hz, 4H, $H_{k'}$), 4.14 (t, ^3J = 7.3 Hz, 8H, H_l), 1.87–1.79 (m, 20H, H_m+H_j), 1.39–1.32 (m, 8H, H_n), 0.97 ppm (t, ^3J = 7.4 Hz, 12H, H_o). ^{13}C{^{1}H} NMR (100 MHz, CD_3CN): δ = 153.8 (C_a), 141.0, 136.5, 123.8, 123.4, 123.4, 121.9 (C_b, C_c, C_e, C_f, C_g, C_h), 100.6 (C_k), 50.4 (C_l), 43.8 (C_d), 32.6 (C_i), 32.4 (C_m), 19.9 (C_n), 16.0 (C_j), 13.6 ppm (C_o). ESI-TOF MS: *m/z* = 1401.6418 (calcd for

[H_4-**1**$(BF_4)_3]^+$ 1401.6523), 657.3125 (calcd for [H_4-**1**$(BF_4)_2]^{2+}$ 657.3240), 409.2080 (calcd for [H_4-**1**$(BF_4)]^{3+}$ 409.2145).

2.6. Synthesis of compound H4-1(OTf)4

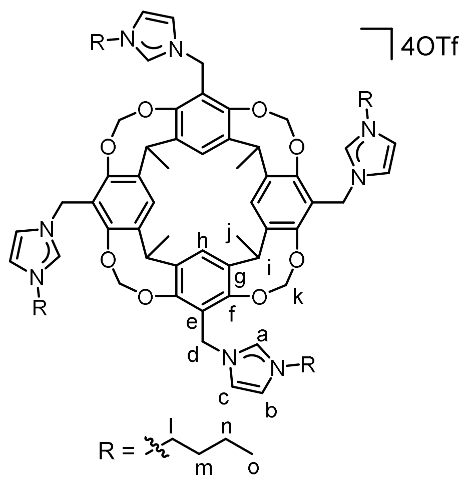

A sample of H_4-**1**$(Br)_4$ (100.0 mg, 0.068 mmol) was dissolved in methanol (10 mL). To this was added NaOTf (94.0 mg, 0.55 mmol) in water (15 mL). After stirring at ambient temperature for 12 h the formed solid was collected by filtration and washed with water and diethyl ether to give compound H_4-**1**$(OTf)_4$ as a colorless solid. Yield: 105.6 mg (0.061 mmol, 90%). ^{1}H NMR (400 MHz, CD_3CN): δ = 8.74 (s, 4H, H_a), 7.75 (s, 4H, H_h), 7.38–7.35 (m, 8H, H_b+H_c), 6.27 (d, ^2J = 7.7 Hz, 4H, H_k), 5.18 (s, 8H, H_d), 4.92 (q, ^3J = 7.5 Hz, 4H, H_i), 4.56 (d, ^2J = 7.7 Hz, 4H, $H_{k'}$), 4.13 (t, ^3J = 7.3 Hz, 8H, H_l), 1.89–1.77 (m, 20H, H_m+H_j), 1.34–1.29 (m, 8H, H_n), 0.94 ppm (t, ^3J = 7.4 Hz, 12H, H_o). $^{13}C\{^1H\}$ NMR (100 MHz, CD_3CN): δ = 153.7 (C_a), 140.9, 136.7, 124.2, 123.4, 123.3, 121.7, 120.2 (C_b, C_c, C_e, C_f, C_g, C_h, $C_{triflat}$), 100.6 (C_k), 50.3 (C_l), 43.8 (C_d), 32.7 (C_i), 32.4 (C_m), 19.8 (C_n), 16.3 (C_j), 13.5 ppm (C_o). ESI-TOF MS: *m/z* = 429.8789 (calcd for [H_4-**1**$(OTf)]^{3+}$ 429.8639), 285.1659 (calcd for [H_4-**1**$]^{4+}$ 285.1598).

2.7. Synthesis of compound H4-1(SbF6)4

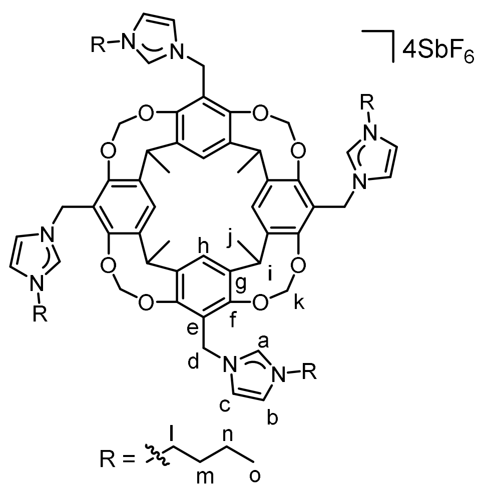

A sample of H_4-**1**$(Br)_4$ (100.0 mg, 0.068 mmol) was dissolved in methanol (10 mL). To this was added $NaSbF_6$ (141.2 mg, 0.55 mmol) in water (15 mL) was added. After stirring of the mixture at a,mbient temperature for 12 h the formed solid was collected by filtration and washed with water and diethyl ether to give compound H_4-**1**$(SbF_6)_4$ as a colorless solid. Yield: 127.4 mg (0.061 mmol, 90%). ^{1}H NMR (400 MHz, CD_3CN): δ= 8.52 (s, 4H, H_a), 7.66 (s, 4H, H_h), 7.37 (d, ^3J = 1.8 Hz, 4H, H_b/H_c), 7.35 (d, ^3J = 1.8 Hz, 4H, H_b/H_c), 6.20 (d, ^2J = 7.6 Hz, 4H, H_k), 5.12 (s, 8H, H_d), 4.91 (q, ^3J = 7.5 Hz, 4H, H_i), 4.56 (d, ^2J = 7.6 Hz, 4H, $H_{k'}$), 4.11 (t, ^3J = 7.3

Hz, 8H, H_l), 1.86–1.76 (m, 20H, H_m+H_j), 1.36–1.27 (m, 8H, H_n), 0.94 ppm (t, ^3J = 7.4 Hz, 12H, H_o). $^{13}C\{^1H\}$ NMR (100 MHz, CD_3CN): δ = 153.6 (C_a), 140.6, 136.3, 123.7, 123.3, 123.2, 121.6 (C_b, C_c, C_e, C_f, C_g, C_h), 100.3 (C_k), 50.3 (C_l), 43.8 (C_d), 32.5 (C_i), 32.4 (C_m), 19.8 (C_n), 16.0 (C_j), 13.5 ppm (C_o). ESI-TOF MS: *m/z* = 806.2027 (calcd for $[H_4\text{-}\mathbf{1}(SbF_6)_2]^{2+}$ 806.2150), 458.5025 (calcd for $[H_4\text{-}\mathbf{1}(SbF_6)]^{3+}$ 458.5113).

3. 3. Synthesis of assemblies $[\{Ag(CH_3CN)_4(BF_4)_8\}\subset Ag_{12}(\mathbf{1})_6](BF_4)_5$, $[(OTf)_8\subset Ag_{12}(\mathbf{1})_6](OTf)_4$ and $[(SbF_6)\subset Ag_4(\mathbf{1})_2](SbF_6)_3$

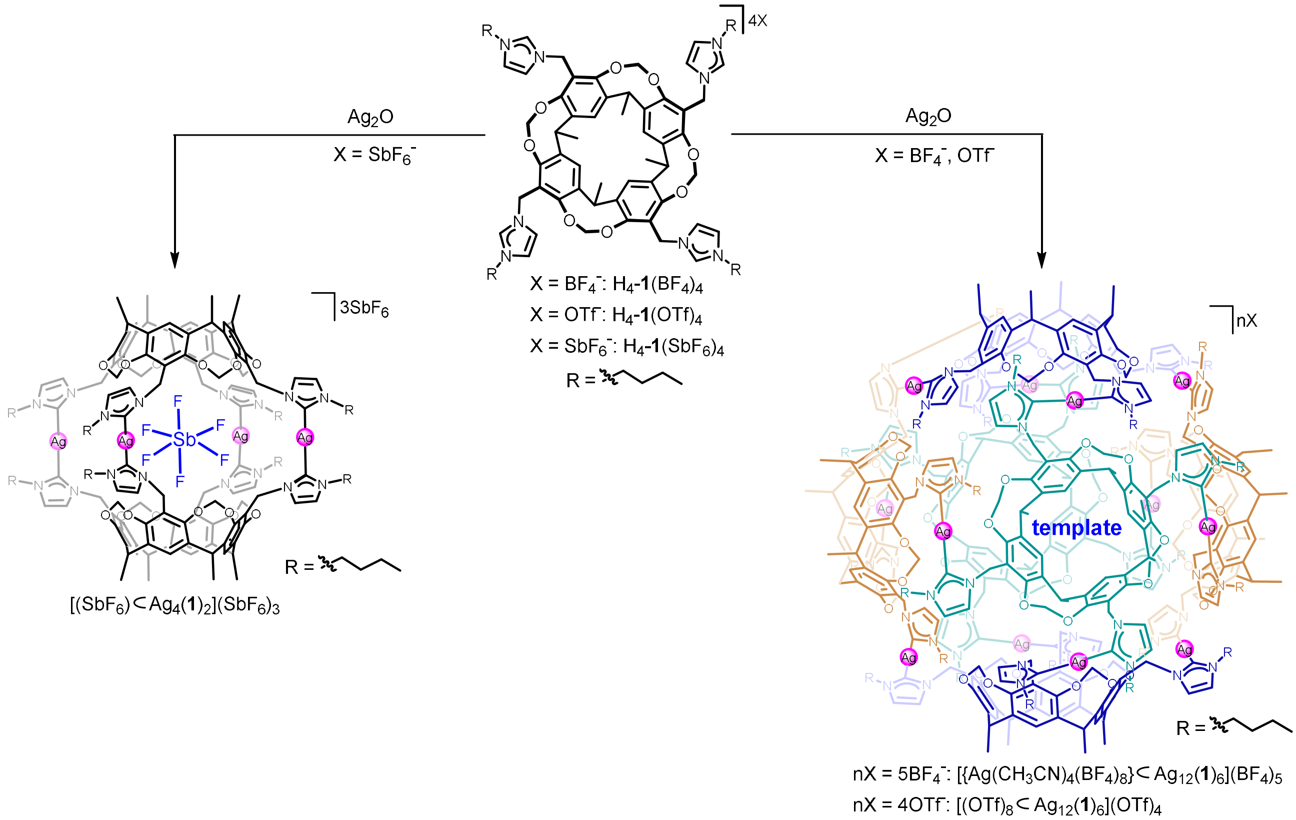

Scheme S2. General synthesis of complexes $[\{Ag(CH_3CN)_4(BF_4)_8\}\subset Ag_{12}(\mathbf{1})_6](BF_4)_5$, $[(OTf)_8\subset Ag_{12}(\mathbf{1})_6](OTf)_4$ and $[(SbF_6)\subset Ag_4(\mathbf{1})_2](SbF_6)_3$.

A sample (0.03–0.05 mmol) of one of the tetra-NHC precursors $H_4\text{-}\mathbf{1}(X)_4$ (X = BF_4^-, OTf^- or SbF_6^- was dissolved in CH_3CN (20 mL). To this solution was added Ag_2O (2 equiv.) under a nitrogen atmosphere. The reaction mixture was then heated for 18 h to 80 °C under exclusion of light. After cooling of the reaction mixture to ambient temperature, the obtained suspension was slowly filtered through a pad of Celite to obtain a clear filtrate. The filtrate was concentrated to 2 mL and diethyl

ether (30 mL) was added. The gray precipitate formed was isolated by filtration, washed with diethyl ether and dried *in vacuo* to afford the complexes $[\{Ag(CH_3CN)_4(BF_4)_8\}\subset Ag_{12}(\mathbf{1})_6](BF_4)_5$, $[(OTf)_8\subset Ag_{12}(\mathbf{1})_6](OTf)_4$ and $[(SbF_6)\subset Ag_4(\mathbf{1})_2](SbF_6)_3$.

3.1. Synthesis of assembly $[\{Ag(CH_3CN)_4(BF_4)_8\}\subset Ag_{12}(\mathbf{1})_6](BF_4)_5$

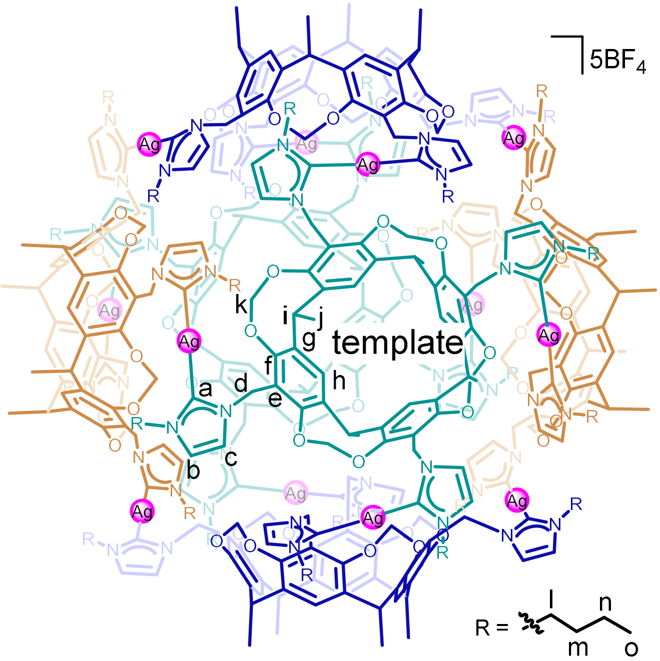

Compound $[\{Ag(CH_3CN)_4(BF_4)_8\}\subset Ag_{12}(\mathbf{1})_6]$-$(BF_4)_5$ was prepared from H_4-$\mathbf{1}(BF_4)_4$ (50.0 mg, 0.034 mmol) and Ag_2O (15.6 mg, 0.067 mmol). Yield: 46.1 mg (0.005 mmol, 88% for the solvent-free compound). ^1H NMR (400 MHz, CD_3CN): $\delta = 7.69$ (s, 24H, H_h), 7.19–7.13 (m, 24H, H_b/H_c), 6.98–6.88 (m, 24H, H_b/H_c), 6.22–6.00 (m, 24H, H_k), 5.11 (s, 48H, H_d), 4.95 (q, ^3J = 7.1 Hz, 24H, H_i), 4.52–4.39 (m, 24H, $H_{k'}$), 4.20–4.13 (m, 48H, H_l), 1.90–1.74 (m, 120H, H_m+H_j), 1.41–1.33 (m, 48H, H_n), 0.97 ppm (t, ^3J = 7.1 Hz, 72H, H_o). Note that due to the very limited solubility of assembly $[\{Ag(CH_3CN)_4(BF_4)_8\}\subset Ag_{12}(\mathbf{1})_6](BF_4)_5$, an informative ^{13}C NMR spectrum could not be obtained. However, all peaks in the range of 4.0 and 8.0 ppm displayed a single diffusion constant in the diffusion-ordered ^1H NMR (DOSY) spectrum, indicating the formation of the single species (Figure S17). ^{19}F NMR (376 MHz, CD_3CN): $\delta = -151.58$ (encapsulated tetrafluoroborate), –151.63 (free tetrafluoroborate). ESI-TOF MS: m/z = 1745.3153 (calcd for $[[Ag_{12}(\mathbf{1})_6](BF_4)_7]^{5+}$ 1745.3127). The mass spectrum featured only peaks for Ag_{12} species.

3.2. Synthesis of assembly [(OTf)$_8$⊂Ag$_{12}$(1)$_6$](OTf)$_4$

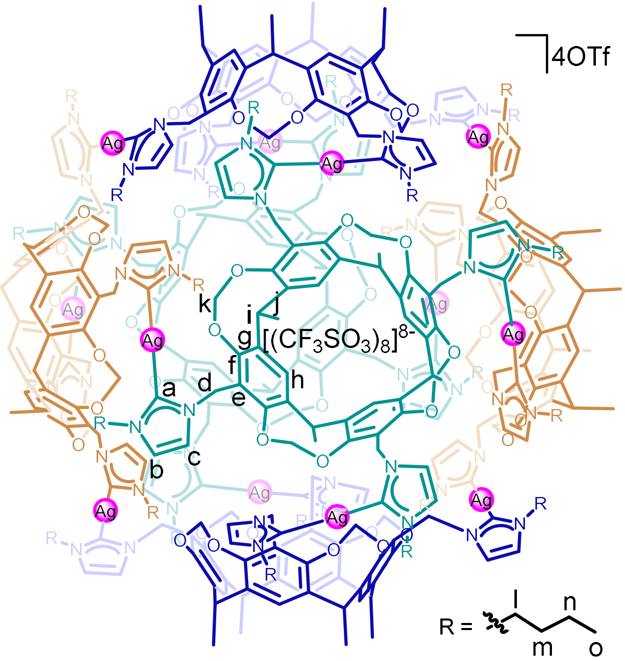

Compound [(OTf)$_8$⊂Ag$_{12}$(**1**)$_6$](OTf)$_4$ was prepared from H$_4$-**1**(OTf)$_4$ (50.0 mg, 0.029 mmol) and Ag$_2$O (16.1 mg, 0.069 mmol). Yield: 42.9 mg (0.004 mmol, 83%). ^{1}H NMR (600 MHz, CD$_3$CN): δ = 7.62 (s, 24H, H$_h$), 7.15 (d, ^3J = 1.8 Hz, 24H, H$_b$), 6.95 (d, ^3J = 1.8 Hz, 24H, H$_c$), 6.04 (d, ^2J = 6.8 Hz, 24H, H$_k$), 5.04 (s, 48H, H$_d$), 4.88 (q, ^3J = 7.5 Hz, 24H, H$_i$), 4.55 (d, ^2J = 6.8 Hz, 24H, H$_{k'}$), 4.18–4.15 (m, 48H, H$_l$), 1.88–1.83 (m, 48H, H$_m$), 1.81 (d, ^3J = 7.5 Hz, 72H, H$_j$), 1.42–1.35 (m, 48H, H$_n$), 0.97 ppm (t, ^3J = 7.4 Hz, 72H, H$_o$). ^{13}C{^{1}H} NMR (150 MHz, CD$_3$CN): δ = 180.3 (from HMBC, C$_a$), 153.8 (C$_f$), 153.4 (C$_f$), 140.3 (C$_g$), 140.2 (C$_g$), 123.3 (C$_h$), 123.1 (C$_b$), 122.5 (C$_e$), 121.9 (C$_c$), 121.0 ($CF_3SO_3^-$), 99.7 (C$_k$), 52.4 (C$_l$), 46.5 (C$_d$), 34.4 (C$_m$), 32.4 (C$_i$), 20.6 (C$_n$), 16.2 (C$_j$), 13.9 ppm (C$_o$). ^{19}F NMR (376 MHz, CD$_3$CN): δ = –79.25 (encapsulated OTf $^-$), –79.92 (free OTf $^-$). ESI-TOF MS: m/z = 2327.7861 (calcd for [[Ag$_{12}$(**1**)$_6$](OTf)$_8$]$^{4+}$ 2327.7859), 1832.6437 (calcd for [[Ag$_{12}$(**1**)$_6$](OTf)$_7$]$^{5+}$ 1832.6445).

3.3. Synthesis of assembly [(SbF$_6$)⊂Ag$_4$(1)$_2$](SbF$_6$)$_3$

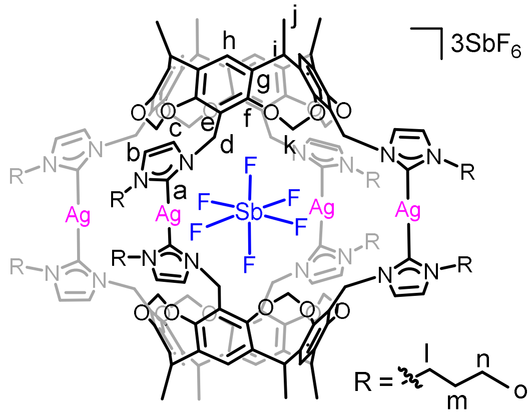

Compound [(SbF$_6$)⊂Ag$_4$(**1**)$_2$](SbF$_6$)$_3$ was prepared from H$_4$-**1**(SbF$_6$)$_4$ (50.0 mg, 0.024 mmol) and Ag$_2$O (11.1 mg, 0.048 mmol). Yield: 39.7 mg (0.011 mmol, 92%). ^{1}H NMR (600 MHz, CD$_3$CN): δ = 7.61 (s, 8H, H$_h$), 7.15 (s, br, 8H, H$_b$), 6.95 (s, br, 8H, H$_c$), 6.06 (d, ^2J = 6.6 Hz, 8H, H$_k$), 5.09–5.02 (m, 16H, H$_d$), 4.89–4.87 (m, 8H, H$_i$), 4.65–4.45 (m, 8H, H$_{k'}$), 4.18–4.16 (m, 16H, H$_l$), 1.88–1.83 (m, 16H, H$_m$), 1.81 (d, J = 7.3 Hz, 24H, H$_j$), 1.41–1.35 (m, 16H, H$_n$), 0.97 ppm (t, ^3J = 7.3 Hz, 24H, H$_o$). ^{13}C{^{1}H} NMR (150 MHz, CD$_3$CN): δ = 180.1 (d, $^1J_{CAg}$) = 212.4 Hz, from HMBC, C$_a$), 153.8, 153.78, 153.75, 153.70, 153.4, 153.35, 153.30, 153.26 (C$_f$), 140.5, 140.36, 140.31, 140.26, 140.22, 140.1 (C$_g$), 123.3 (C$_b$), 123.1 (C$_h$), 122.9 (C$_{b'}$), 122.7 (C$_e$),

122.4 ($C_{e'}$), 121.93 (C_c), 121.89 ($C_{c'}$), 99.8 (C_k), 99.7 ($C_{k'}$), 52.4 (C_l), 46.5 (C_d), 46.4 ($C_{d'}$), 34.4 (C_m), 32.4 (C_i), 20.6 (C_n), 16.16 (C_j), 16.13 ($C_{j'}$), 13.9 ppm (C_o). ESI-TOF MS: *m/z* = 980.5311 (calcd for $[[Ag_4(\mathbf{1})_2](SbF_6)]^{3+}$ 980.5788), 676.6748 (calcd for $[Ag_4(\mathbf{1})_2]^{4+}$ 676.7109). The ^{19}F NMR spectrum (Figure S29) features multiple peaks in the range of $\delta = -110$ to -138 ppm due to coupling with ^{121}Sb (I = 5/2) and ^{123}Sb (I = 7/2). The spectrum is not informative regarding the encapsulation of SbF_6^- anions.

4. Synthesis of assemblies $[Au_4(1)_2](X)_4$ (X = BF_4^-, OTf^-) and of $[(SbF_6)\subset Au_4(1)_2](SbF_6)_3$

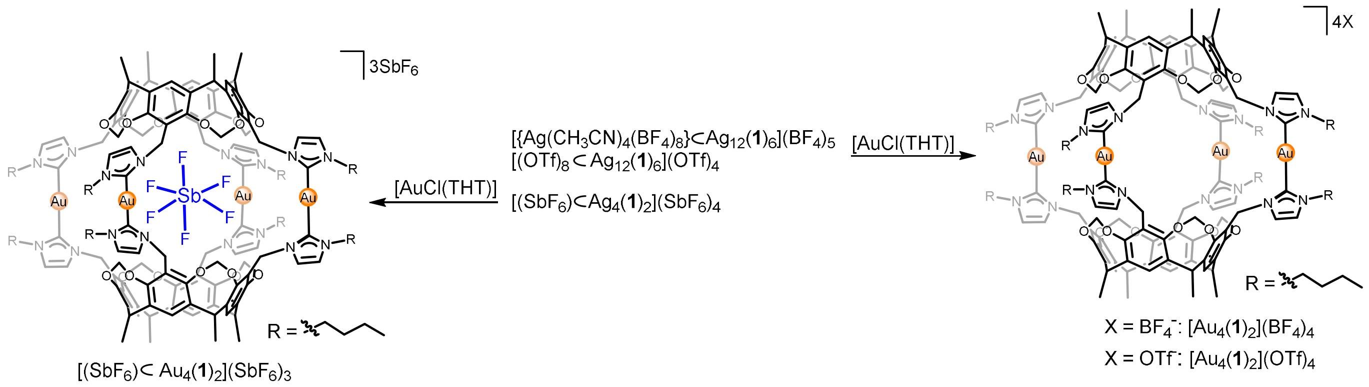

Scheme S3. General synthesis of assemblies $[Au_4(\mathbf{1})_2](X)_4$ (X = BF_4^-, OTf^-) and of $[(SbF_6)\subset Au_4(\mathbf{1})_2](SbF_6)_3$.

To a solution of $[\{Ag(CH_3CN)_4(BF_4)_8\}\subset Ag_{12}(\mathbf{1})_6](BF_4)_5$ or $[(OTf)_8\subset Ag_{12}(\mathbf{1})_6](OTf)_4$ or $[(SbF_6)\subset Ag_4(\mathbf{1})_2](SbF_6)_3$ in CH_3CN (20 mL) was added [AuCl(THT)] (THT = tetrahydrothiophene) under a nitrogen atmosphere. The reaction mixture was stirred at ambient temperature for 24 h and then slowly filtered through a pad of Celite until a clear filtrate was obtained. The filtrate was concentrated to 2 mL, and diethyl ether (20 mL) was added. An off-white precipitate formed, which was isolated by filtration, washed with diethyl ether and dried *in vacuo* to afford the tetranuclear assemblies $[Au_4(\mathbf{1})_2](X)_4$ (X = BF_4^-, OTf^-) and $[(SbF_6)\subset Au_4(\mathbf{1})_2](SbF_6)_3$ (Scheme S3).

4.1. Synthesis of assembly [Au_4(**1**)$_2$](BF_4)$_4$

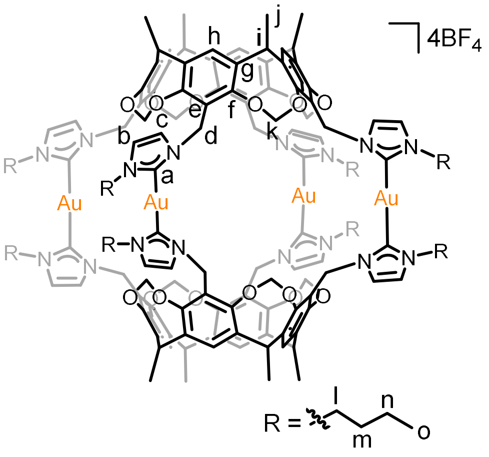

Compound [Au_4(**1**)$_2$](BF_4)$_4$ was prepared from [{Ag(CH_3CN)$_4$(BF_4)$_8$}⊂Ag_{12}(**1**)$_6$](BF_4)$_5$ (46.1 mg, 0.005 mmol) and [AuCl(THT)] (21.4 mg, 0.067 mmol). Yield: 45.4 mg (0.013 mmol, 87%). ^{1}H NMR (600 MHz, CD_3CN): δ = 7.73 (s, 8H, H_h), 7.21 (d, ^3J = 2.0 Hz, 8H, H_b), 6.94 (d, ^3J = 2.0 Hz, 8H, H_c), 6.28 (d, ^2J = 6.8 Hz, 8H, H_k), 5.32 (d, ^2J = 12.7 Hz, 8H, H_d), 5.09 (d, ^2J = 12.7 Hz, 8H, $H_{d'}$), 4.97 (q, ^3J = 7.4 Hz, 8H, H_i), 4.30–4.24 (m, 24H, $H_{k'}$+H_l), 1.92–1.88 (m, 16H, H_m), 1.85 (d, ^3J = 7.4 Hz, 24H, H_j), 1.42–1.36 (m, 16H, H_n), 0.98 ppm (t, ^3J = 7.3 Hz, 24H, H_o). ^{13}C{^{1}H} NMR (150 MHz, CD_3CN): δ = 184.1 (C_a), 153.8 (C_f), 153.4 (C_f), 142.0 (C_g), 141.8 (C_g), 123.9 (C_h), 123.7 (C_b), 122.5 (C_e), 121.9 (C_c), 99.9 (C_k), 51.9 (C_l), 44.8 (C_d), 34.2 (C_m), 32.7 (C_i), 20.6 (C_n), 15.7 (C_j), 13.9 ppm (C_o). ^{19}F NMR (376 MHz, CD_3CN): δ = –151.61 ppm. ESI-TOF MS: m/z = 1049.7183 (calcd for [[Au_4(**1**)$_2$](BF_4)]$^{3+}$ 1049.6972), 765.5321 (calcd for [Au_4(**1**)$_2$]$^{4+}$ 765.5217).

4.2. Synthesis of assembly [Au_4(**1**)$_2$](OTf)$_4$

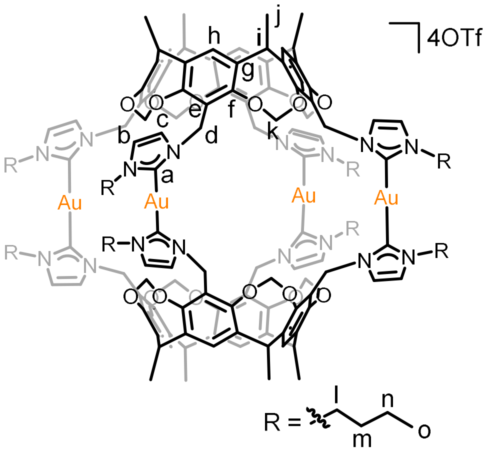

Compound [Au_4(**1**)$_2$](OTf)$_4$ was prepared from [(OTf)$_8$⊂Ag_{12}(**1**)$_6$](OTf)$_4$ (42.9 mg, 0.004 mmol) and [AuCl(THT)] (18.4 mg, 0.057 mmol). Yield: 42.4 mg (0.0115 mmol, 96%). ^{1}H NMR (600 MHz, CD_3CN): δ = 7.61 (s, 8H, H_h), 7.16 (d, ^3J = 1.7 Hz, 8H, H_b), 6.94 (d, ^3J = 1.7 Hz, 8H, H_c), 6.11 (d, ^2J = 6.7 Hz, 8H, H_k), 5.32 (d, ^2J = 12.9 Hz, 8H, H_d), 4.97 (d, ^2J = 12.9 Hz, 8H, $H_{d'}$), 4.89 (q, ^3J = 7.3 Hz, 8H, H_i), 4.55 (d, ^2J = 6.7 Hz, 8H, $H_{k'}$), 4.25 (t, ^3J = 7.1 Hz, 16H, H_l), 1.92–1.87 (m, 16H, H_m), 1.81 (d, ^3J = 7.3 Hz, 24H, H_j), 1.42–1.36 (m, 16H, H_n), 0.98 ppm (t, ^3J = 7.3 Hz, 24H, H_o). ^{13}C{^{1}H} NMR (150 MHz, CD_3CN): δ = 184.0 (C_a), 153.8 (C_f), 153.4 (C_f), 140.4 (C_g), 140.2 (C_g), 123.3 (C_h), 123.1 (C_b), 122.3 (C_e), 122.1 (C_c), 121.0 ($CF_3SO_3^-$), 99.7 (C_k),

51.8 (C_l), 45.4 (C_d), 34.3 (C_m), 32.4 (C_i), 20.6 (C_n), 16.1 (C_j), 14.0 ppm (C_o). ^{19}F NMR (376 MHz, CD_3CN): δ = –79.78 ppm. ESI-TOF MS: m/z = 1680.0478 (calcd for $[[Au_4(\mathbf{1})_2](OTf)_2]^{2+}$ 1679.9961), 1070.4192 (calcd for $[[Au_4(\mathbf{1})_2](OTf)]^{3+}$ 1070.3465).

4.3. Synthesis of assembly $[(SbF_6)\subset Au_4(1)_2](SbF_6)_3$

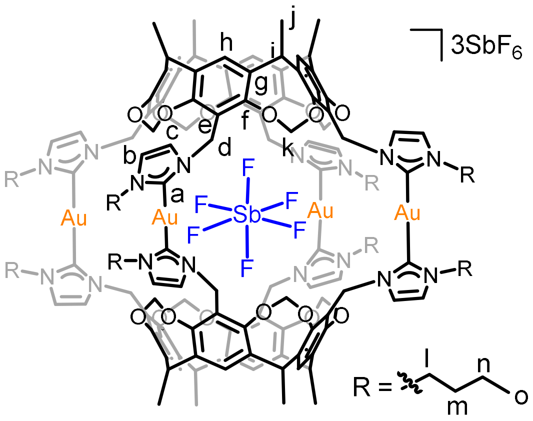

Compound $[(SbF_6)\subset Au_4(\mathbf{1})_2](SbF_6)_3$ was prepared from $[(SbF_6)\subset Ag_4(1)_2](SbF_6)_3$ (39.7 mg, 0.011 mmol) and [AuCl(THT)] (15.4 mg, 0.048 mmol). Yield: 39.3 mg (0.010 mmol, 91%). ^{1}H NMR (600 MHz, CD_3CN): δ = 7.61 (s, 8H, H_h), 7.16 (s, 8H, H_b), 6.94 (s, 8H, H_c), 6.12 (d, ^2J = 6.7 Hz, 8H, H_k), 5.32 (d, ^2J = 12.7 Hz, 8H, H_d), 4.97 (d, ^2J = 12.7 Hz, 8H, $H_{d'}$), 4.89 (q, ^3J = 7.3 Hz, 8H, H_i), 4.55 (d, ^2J = 6.7 Hz, 8H, $H_{k'}$), 4.25 (t, ^3J = 7.1 Hz, 16H, H_l), 1.92–1.87 (m, 16H, H_m), 1.81 (d, ^3J = 7.3 Hz, 24H, H_j), 1.42–1.36 (m, 16H, H_n), 0.98 ppm (t, ^3J = 7.3 Hz, 24H, H_o). ^{13}C{^{1}H} NMR (150 MHz, CD_3CN): δ = 184.0 (C_a), 153.9 (C_f), 153.4 (C_f), 140.4 (C_g), 140.3 (C_g), 123.3 (C_h), 123.1 (C_b), 122.5 (C_e), 122.2 (C_c), 99.8 (C_k), 51.8 (C_l), 45.4 (C_d), 34.3 (C_m), 32.4 (C_i), 20.6 (C_n), 16.1 (C_j), 14.0 ppm (C_o). ESI-TOF MS: m/z = 765.5467 (calcd for $[Au_4(\mathbf{1})_2]^{4+}$ 765.5217). The ^{19}F NMR spectrum (Figure S53) features multiple peaks in the range of δ = −105 to –130 ppm due to coupling with ^{121}Sb (I = 5/2) and ^{123}Sb (I = 7/2). The spectrum is not informative regarding the encapsulation of SbF_6^- anions.

6. Selected NMR and mass spectra of all new compounds

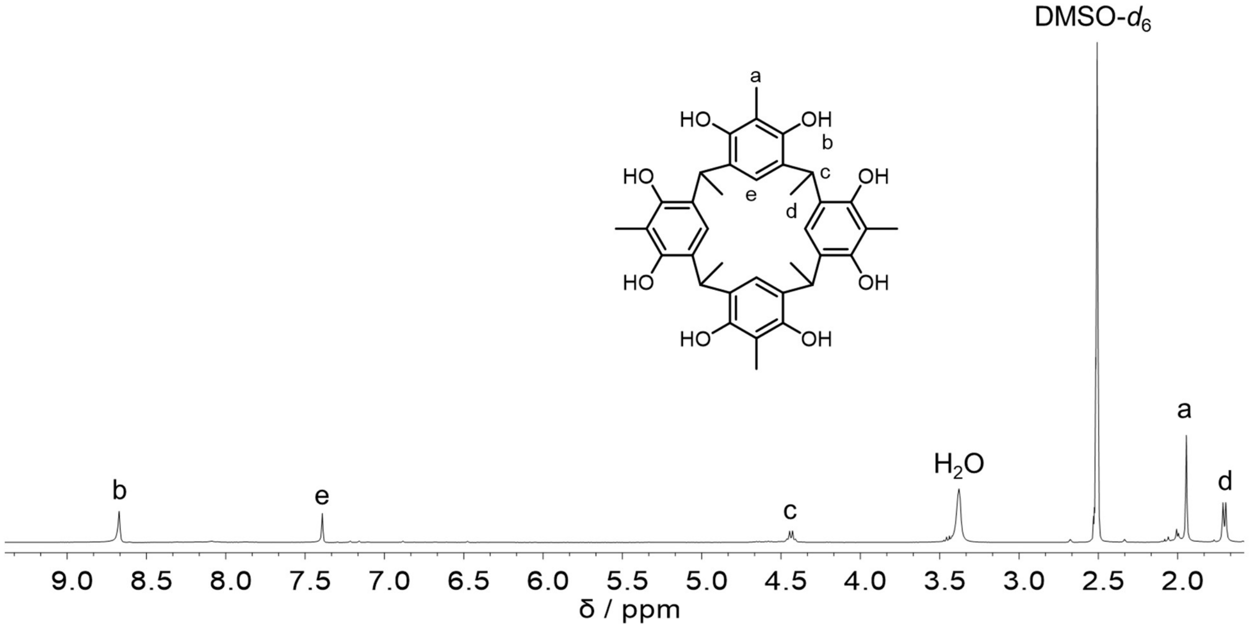

Figure S1. ^{1}H NMR spectrum (400 MHz, DMSO-d_6) of compound **2**.

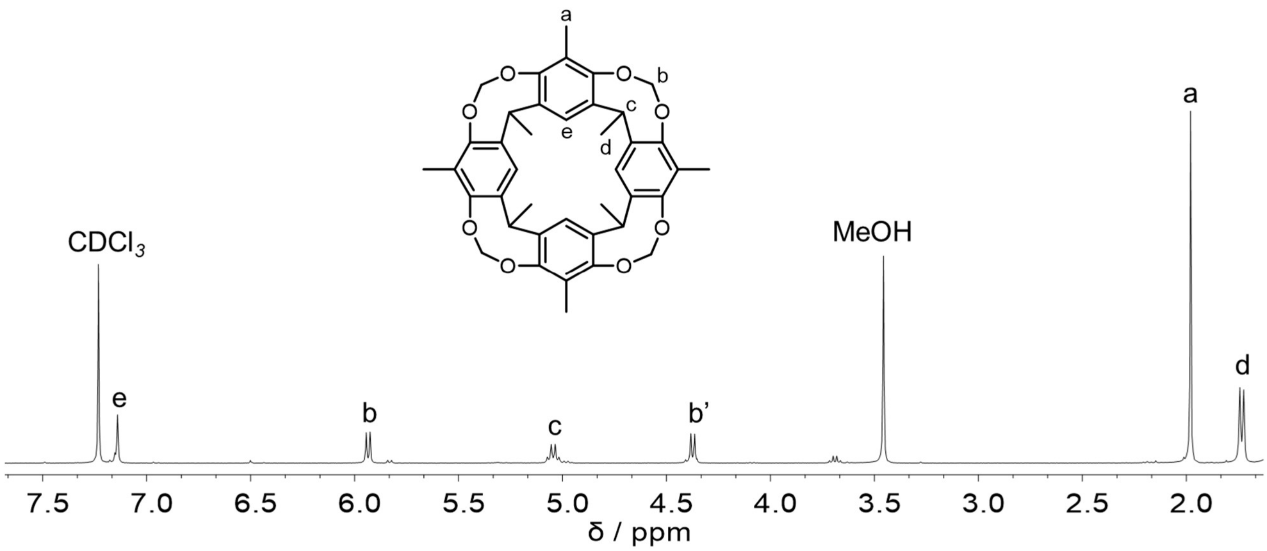

Figure S2. ^{1}H NMR spectrum (400 MHz, $CDCl_3$) of compound **3**.

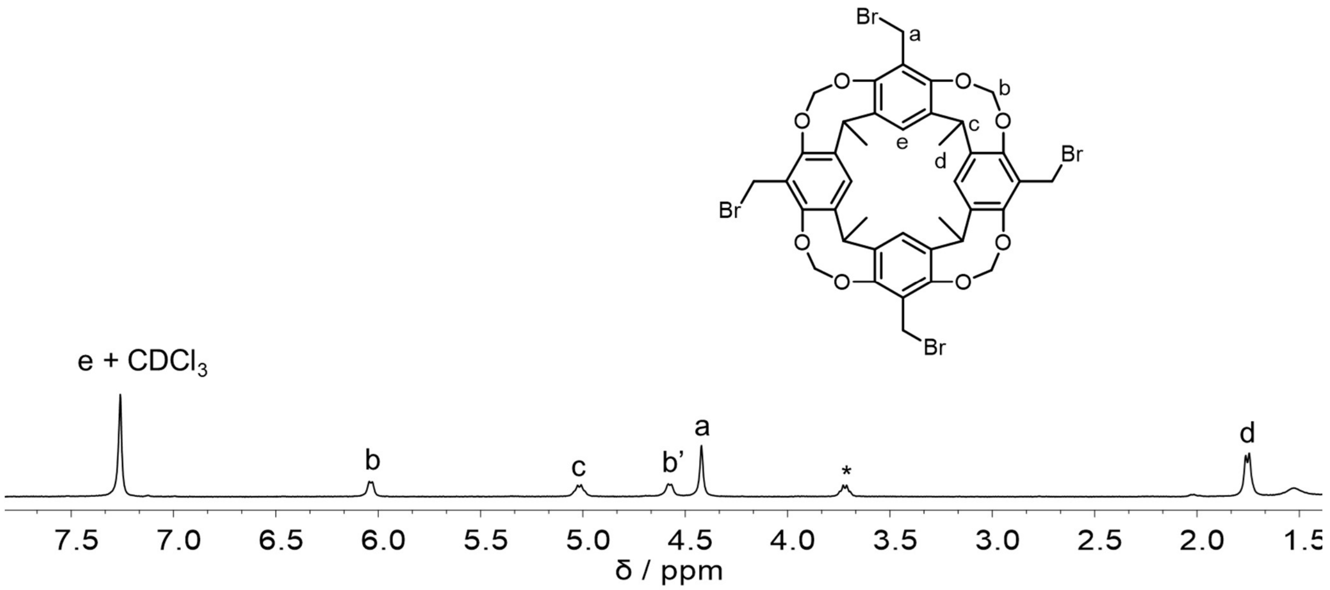

Figure S3. ^{1}H NMR spectrum (400 MHz, $CDCl_3$) of compound **4** (* = MeOH).

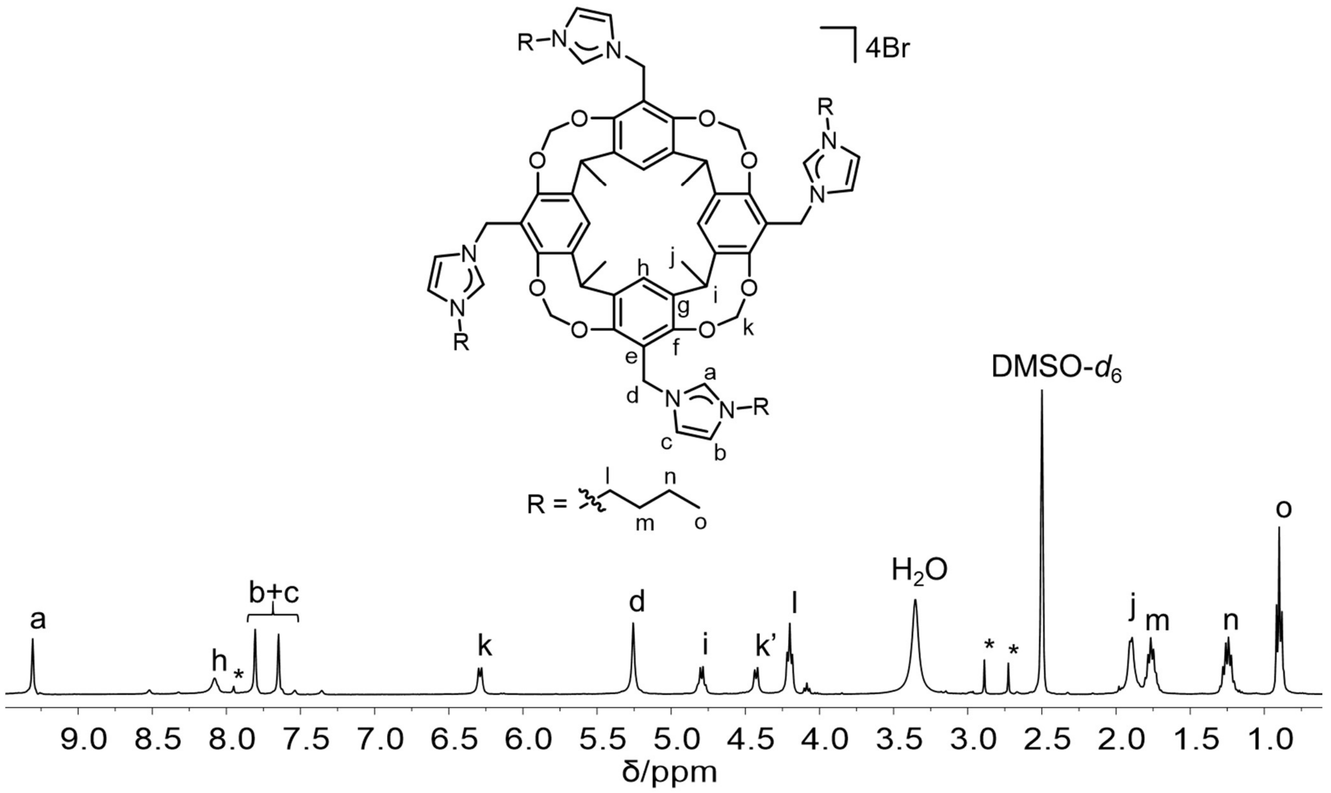

Figure S4. ^{1}H NMR spectrum (400 MHz, DMSO-d_6) of compound H_4-**1**$(Br)_4$ (* = DMF).

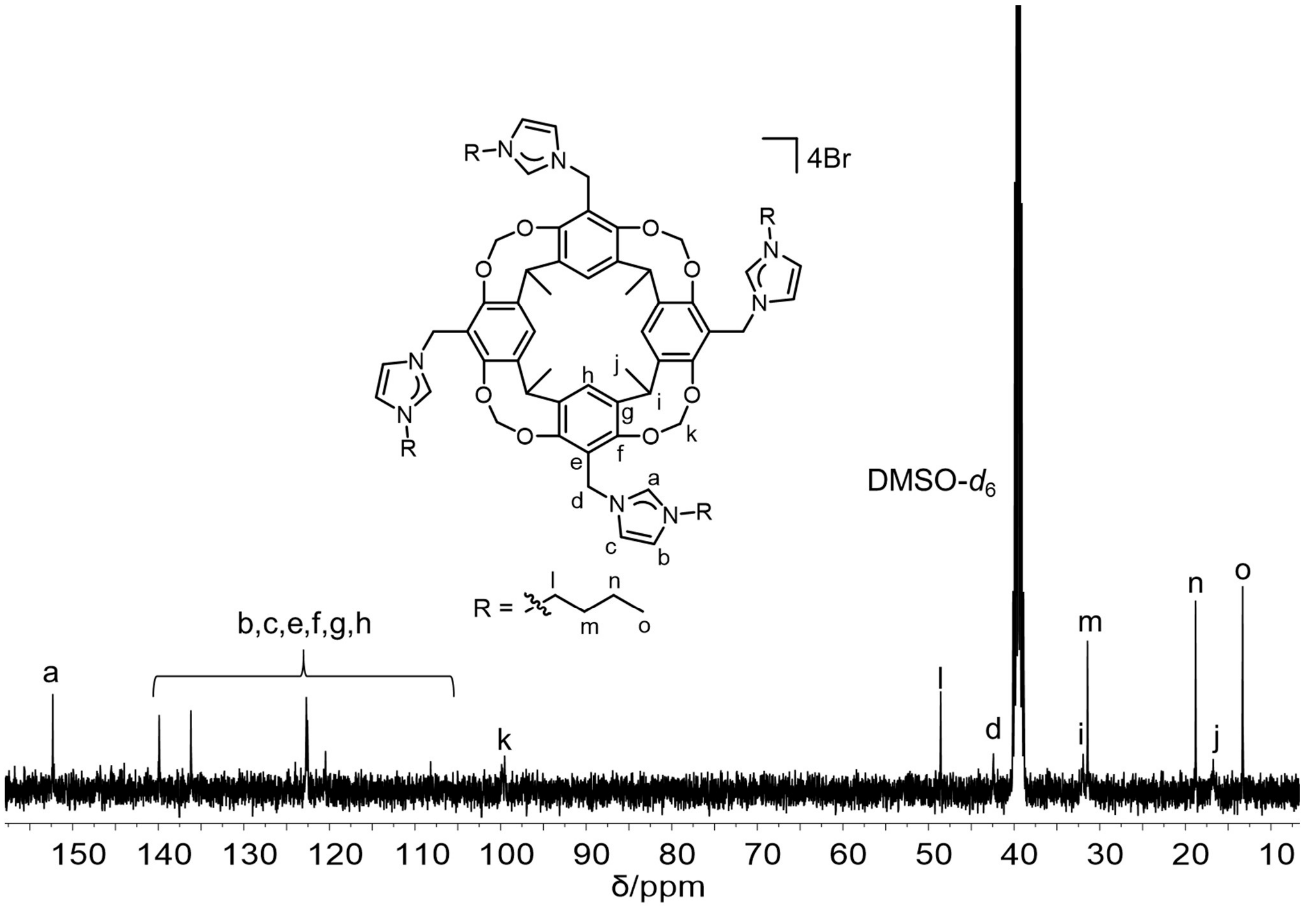

Figure S5. $^{13}C\{^{1}H\}$ NMR spectrum (400 MHz, DMSO-d_6) of compound H_4-**1**$(Br)_4$.

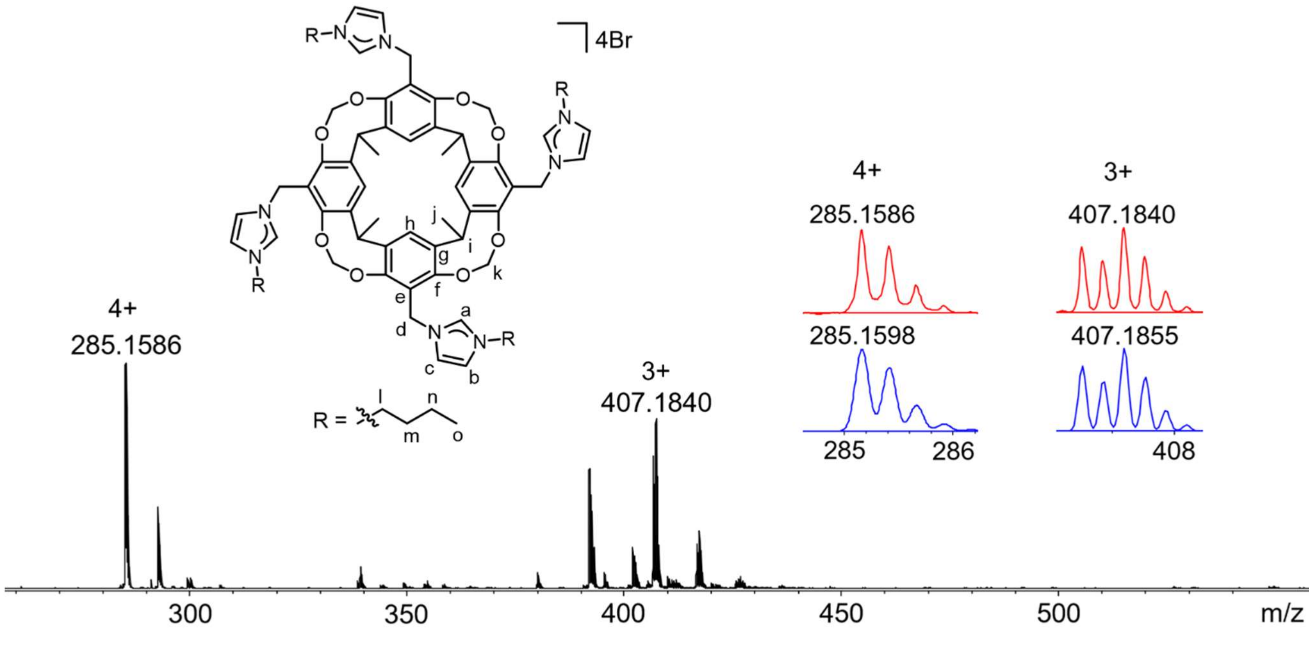

Figure S6. ESI-TOF mass spectrum (positive ions) of compound H_4-**1**$(Br)_4$.

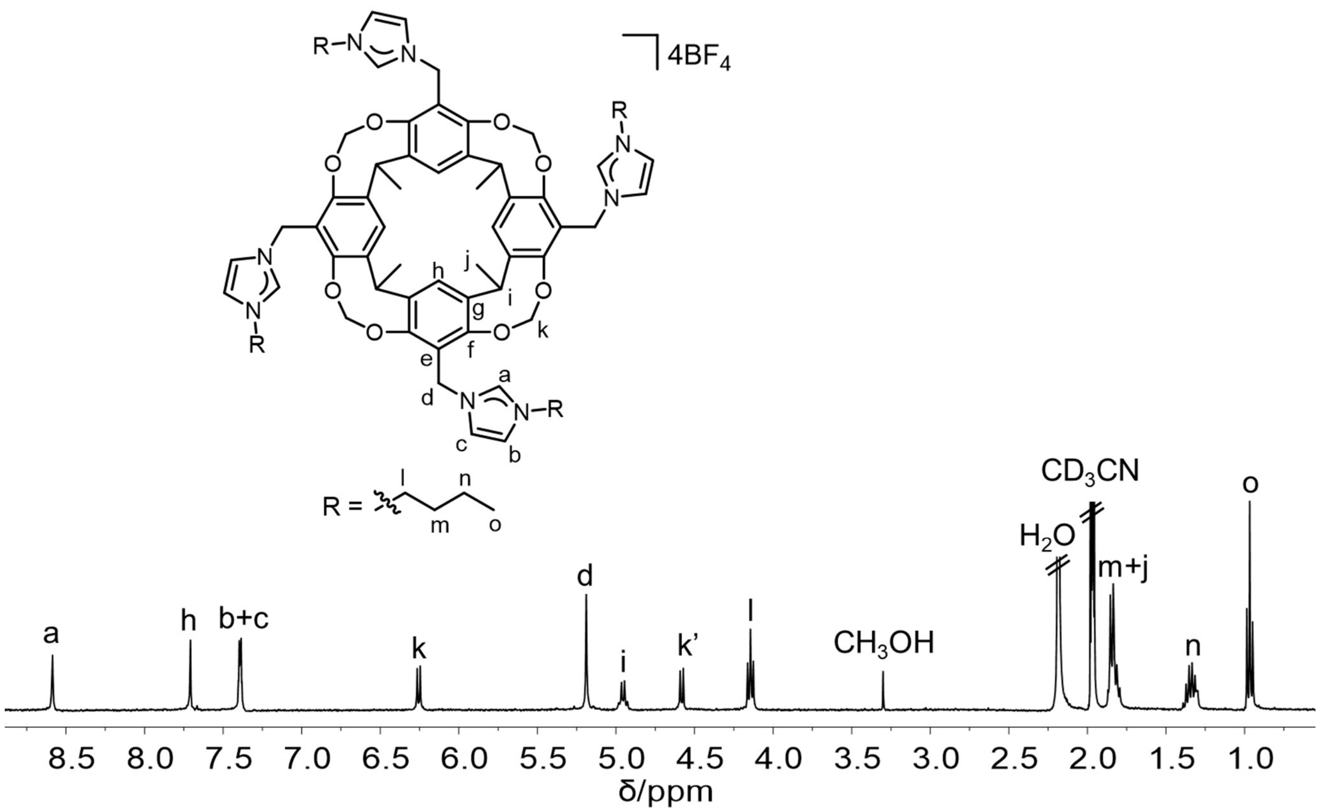

Figure S7. ^{1}H NMR spectrum (400 MHz, CD_3CN) of compound H_4-**1**$(BF_4)_4$.

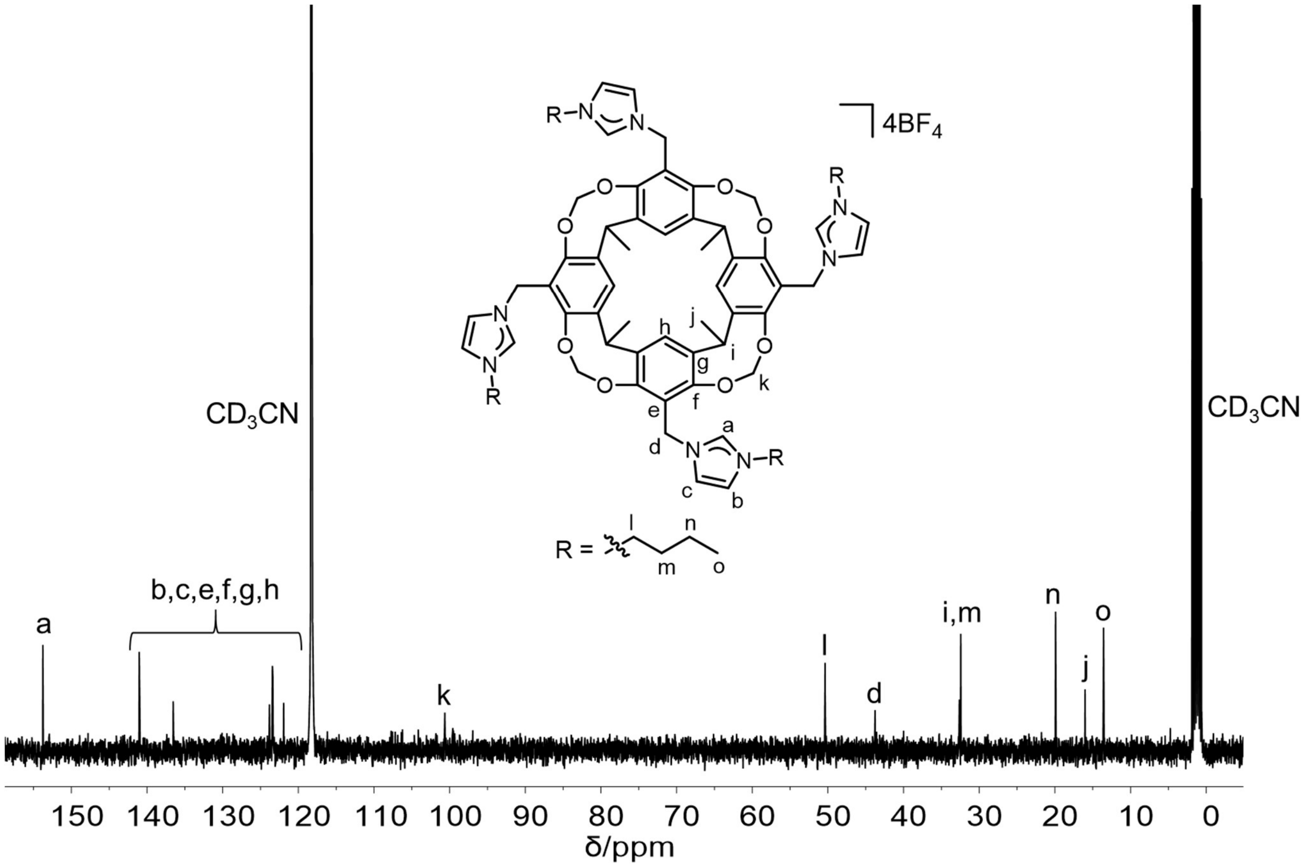

Figure S8. $^{13}C\{^{1}H\}$ NMR spectrum (100 MHz, DMSO-d_6) of compound H_4-**1**$(BF_4)_4$.

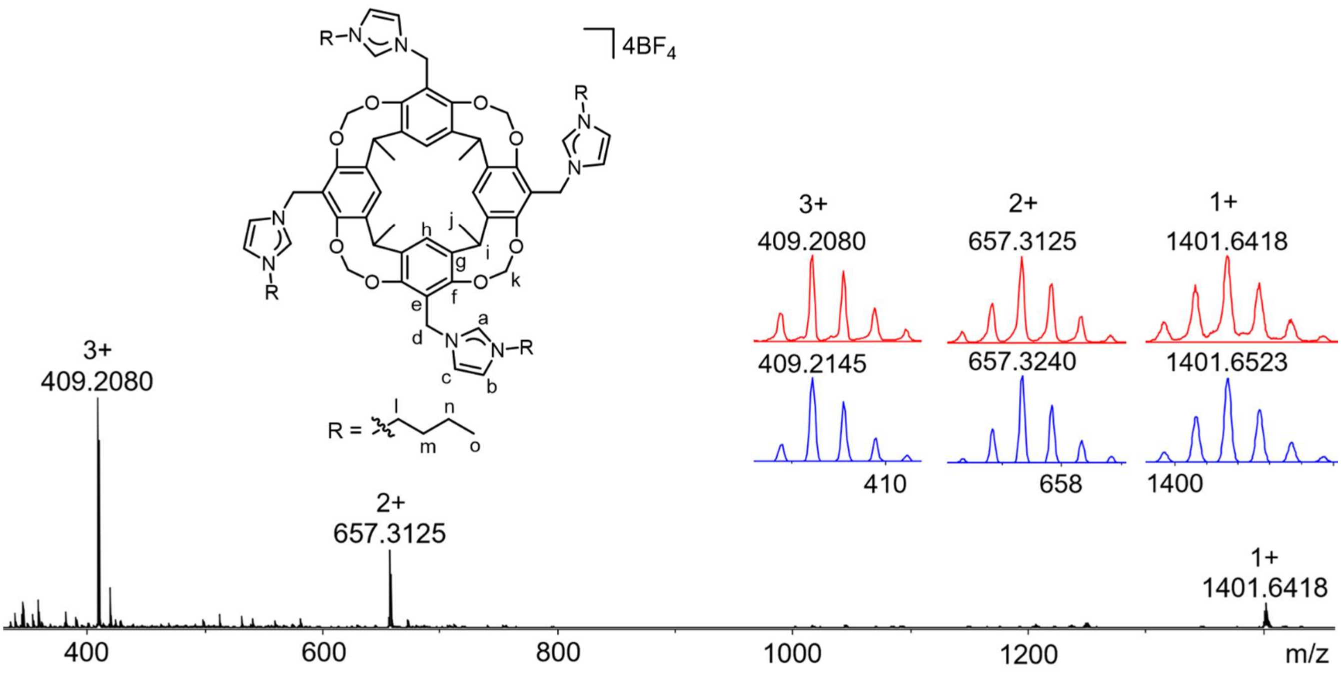

Figure S9. ESI-TOF mass spectrum (positive ions) of compound H_4-**1**$(BF_4)_4$.

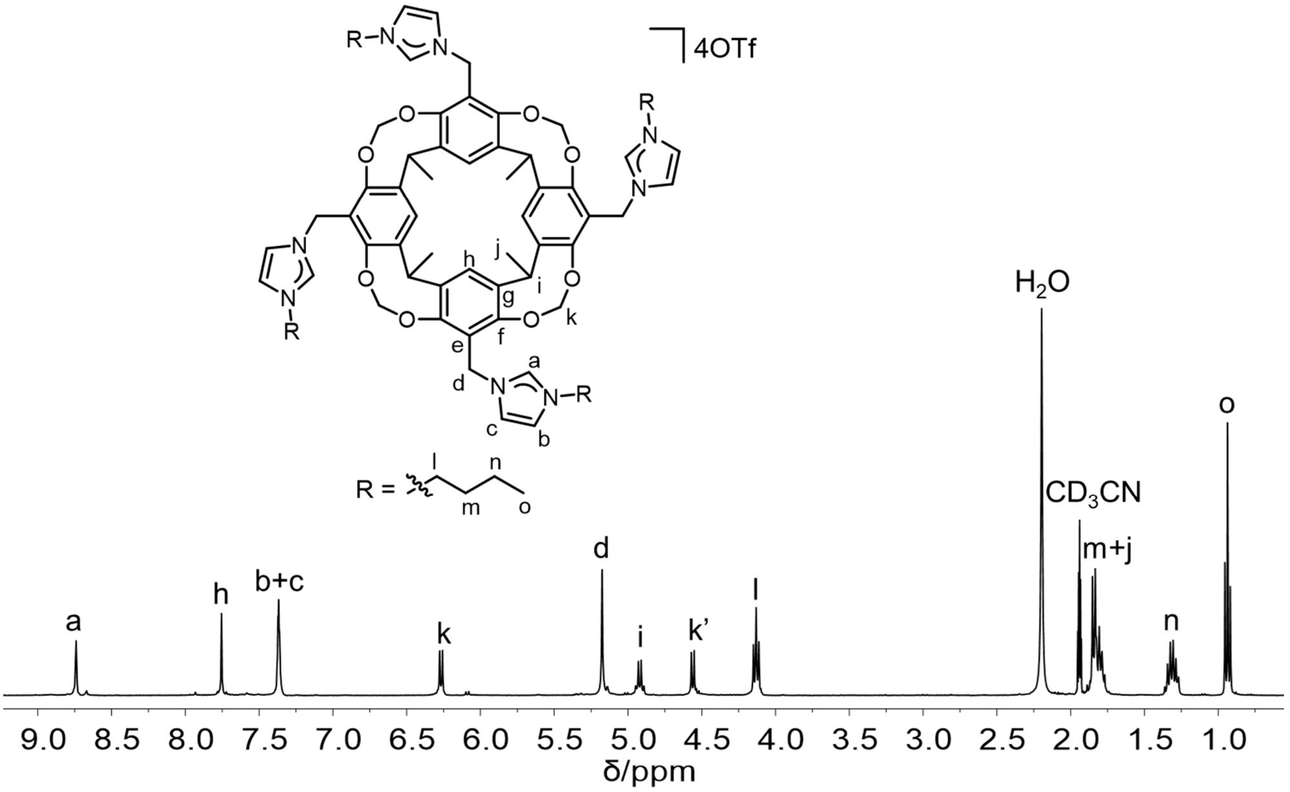

Figure S10. ^{1}H NMR spectrum (400 MHz, CD_3CN) of compound H_4-**1**$(OTf)_4$.

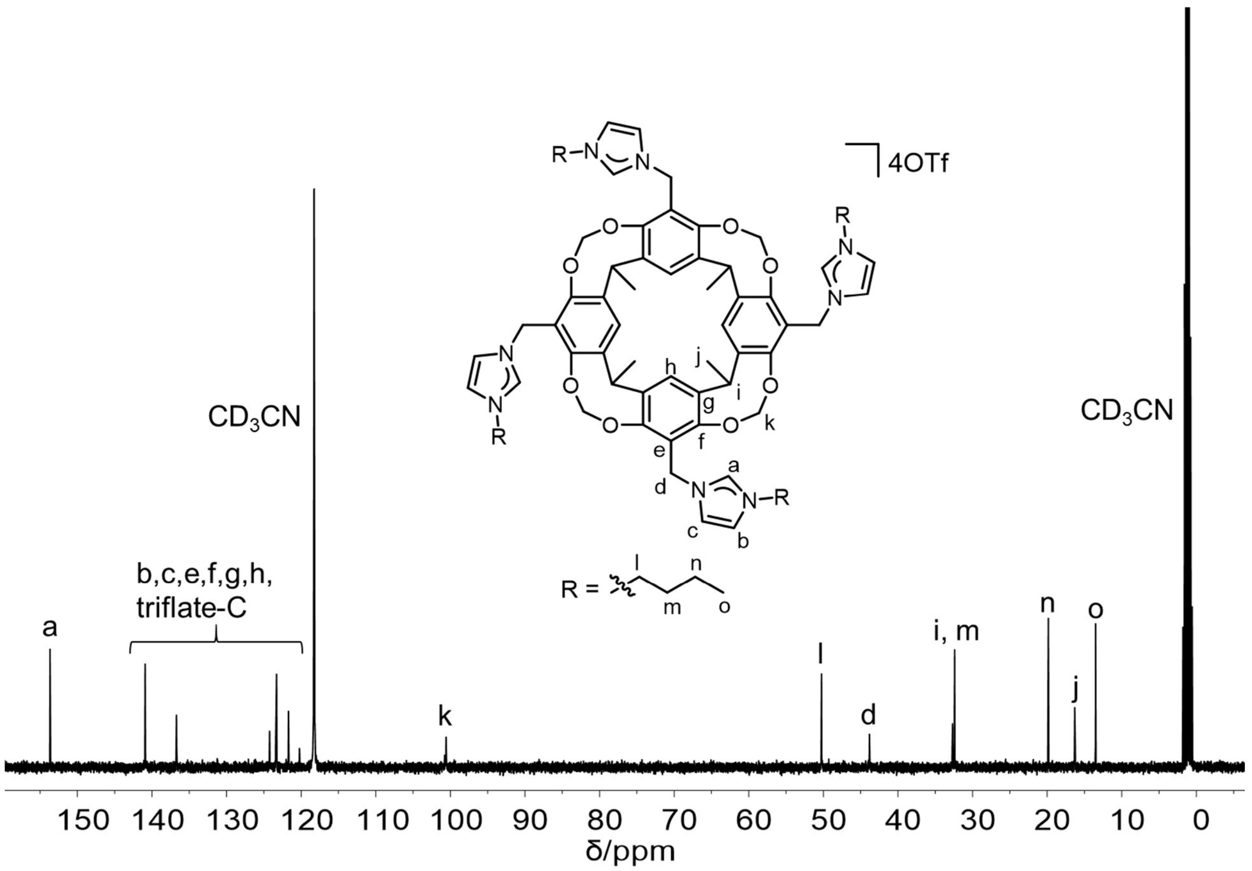

Figure S11. ${}^{13}C\{{}^{1}H\}$ NMR spectrum (100 MHz, CD_3CN) of compound H_4-**1**$(OTf)_4$.

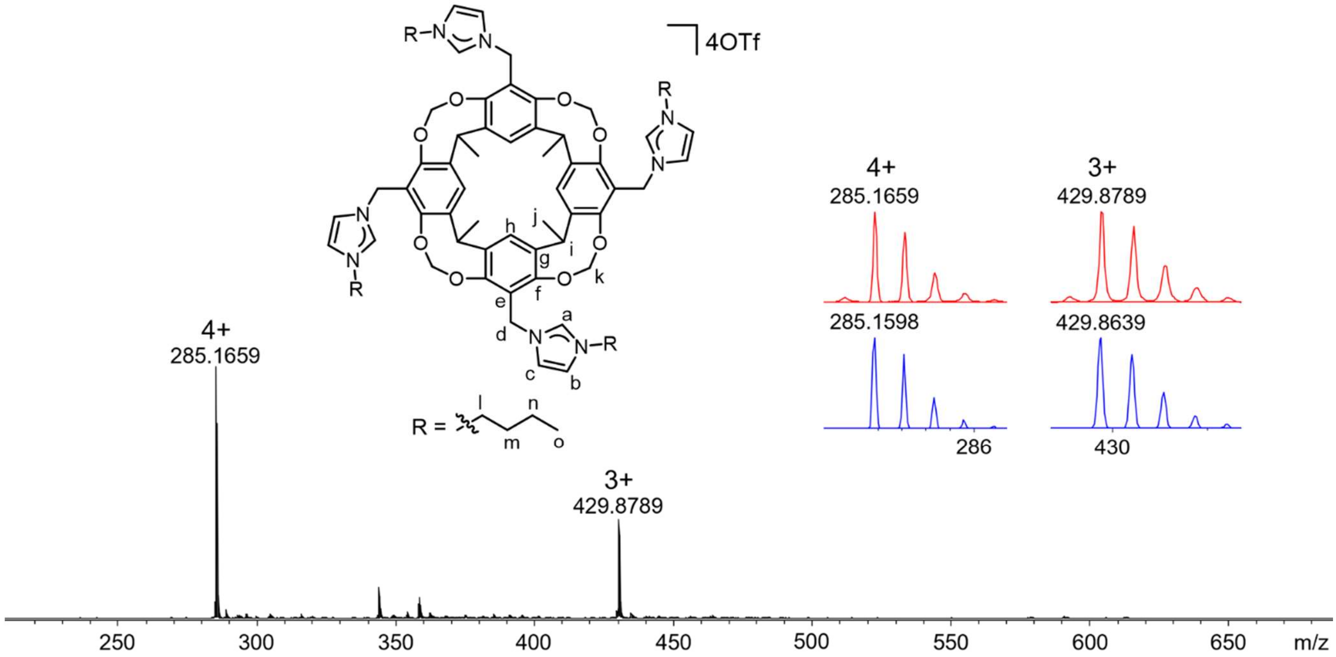

Figure S12. ESI-TOF mass spectrum of H_4-**1**$(OTf)_4$ with isotope distribution for two cation peaks (experimental in red, calculated in blue).

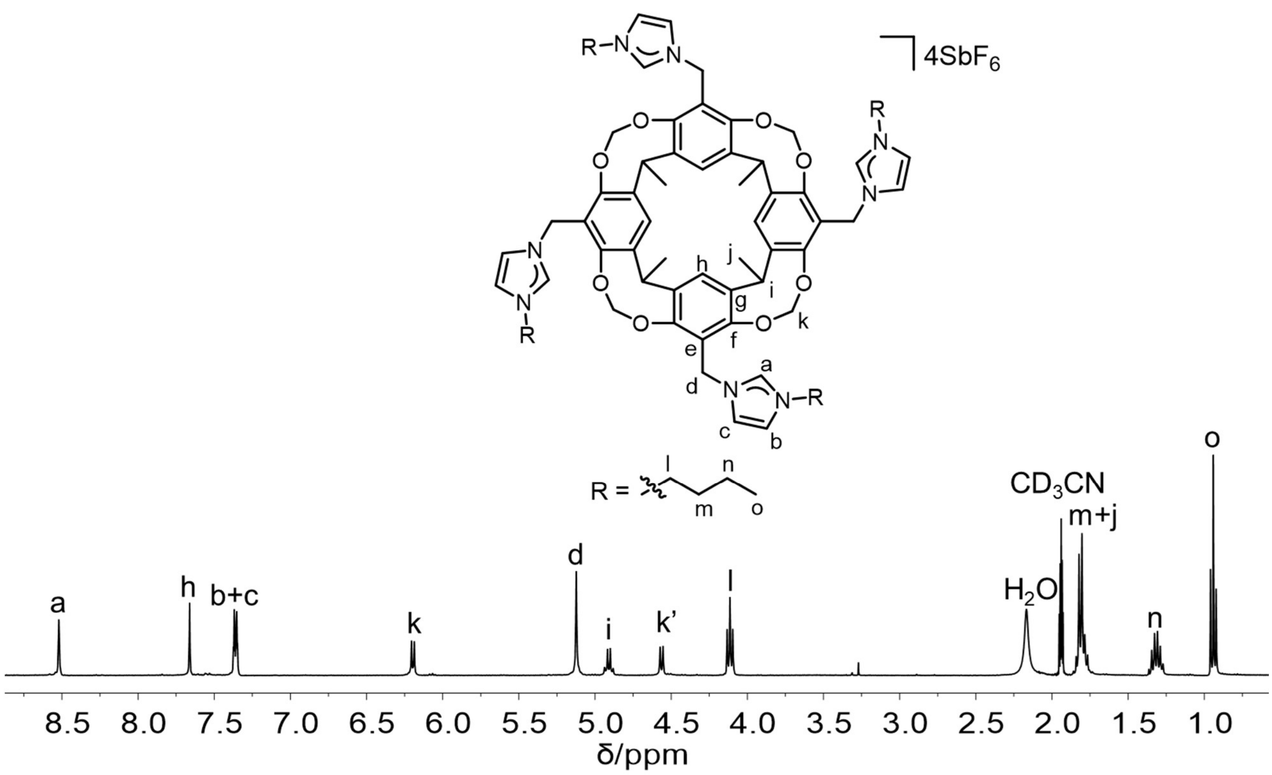

Figure S13. ^{1}H NMR spectrum (400 MHz, CD_3CN) of compound H_4-**1**$(SbF_6)_4$.

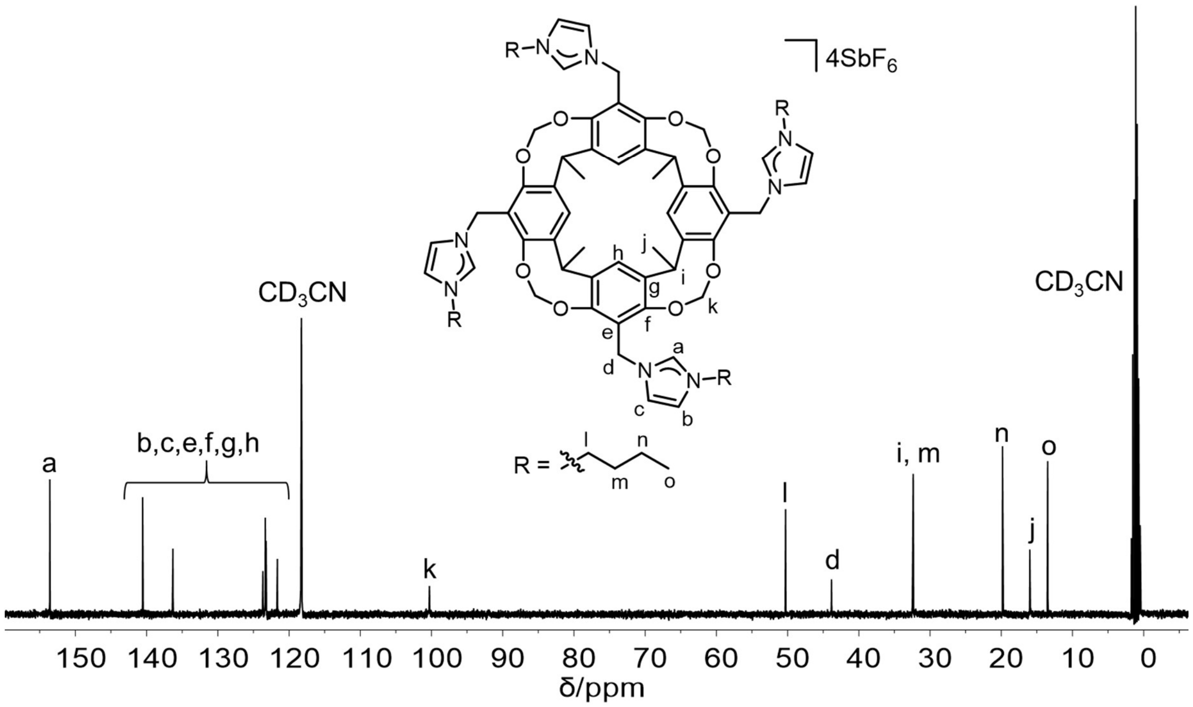

Figure S14. $^{13}C\{^{1}H\}$ NMR spectrum (100 MHz, CD_3CN) of compound H_4-**1**$(SbF_6)_4$.

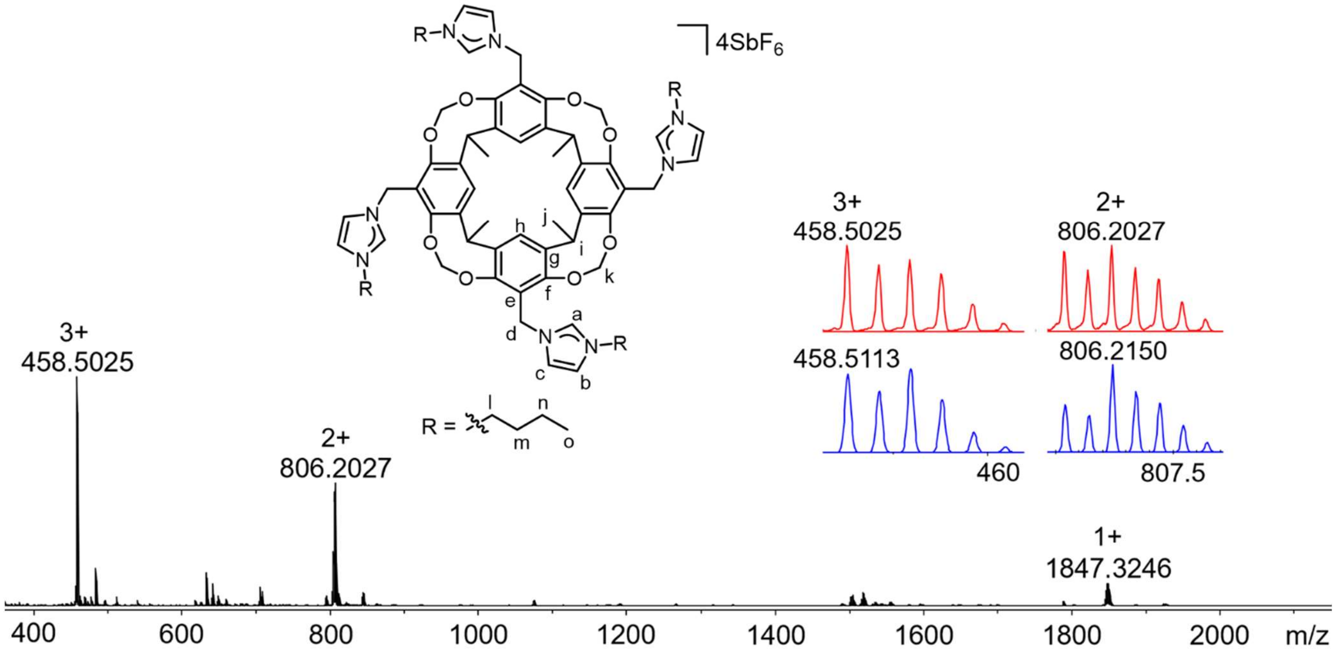

Figure S15. ESI-TOF mass spectrum of H_4-**1**$(SbF_6)_4$ with isotope distribution for two cation peaks (experimental in red, calculated in blue).

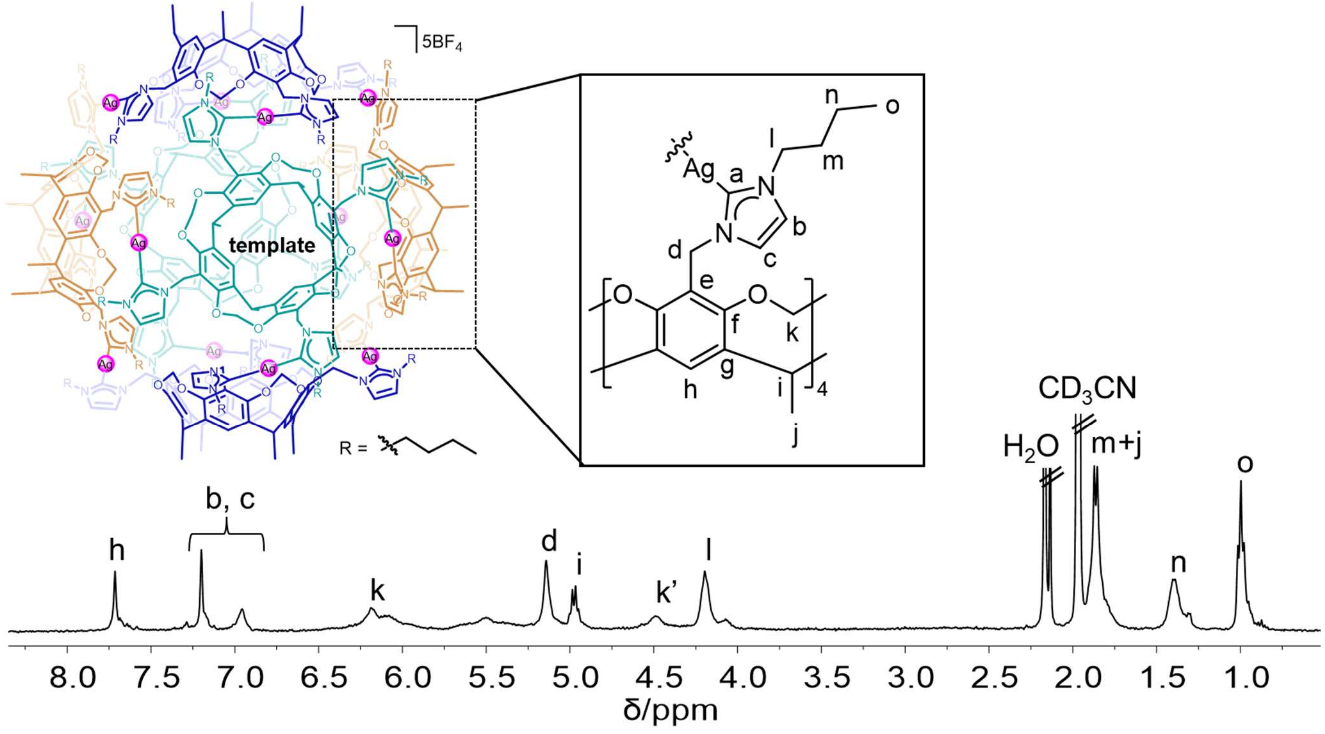

Figure S16. ^{1}H NMR spectrum (400 MHz, CD_3CN) of $[\{Ag(CH_3CN)_4(BF_4)_8\}\subset Ag_{12}(\mathbf{1})_6](BF_4)_5$.

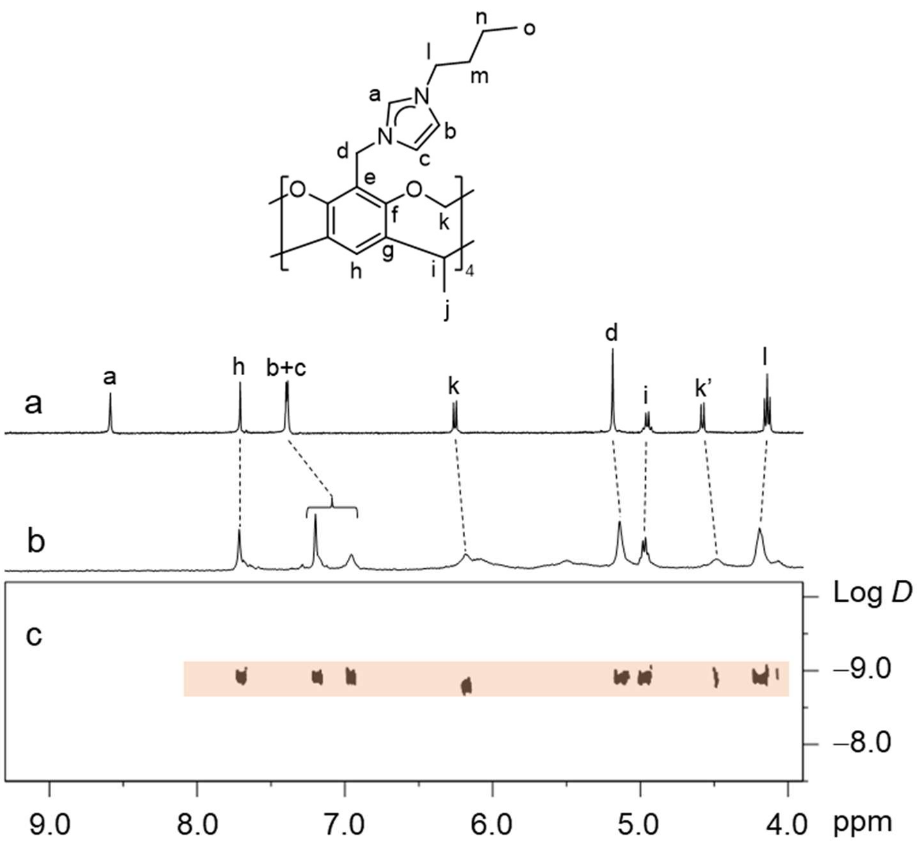

Figure S17. Partial ^{1}H NMR spectra (400 MHz, CD_3CN) of a) H_4-**1**$(BF_4)_4$, b) $[\{Ag(CH_3CN)_4(BF_4)_8\}\subset Ag_{12}(\mathbf{1})_6](BF_4)_5$ and c) ^{1}H DOSY spectrum (400 MHz, CD_3CN) of $[\{Ag(CH_3CN)_4(BF_4)_8\}\subset Ag_{12}(\mathbf{1})_6](BF_4)_5$.

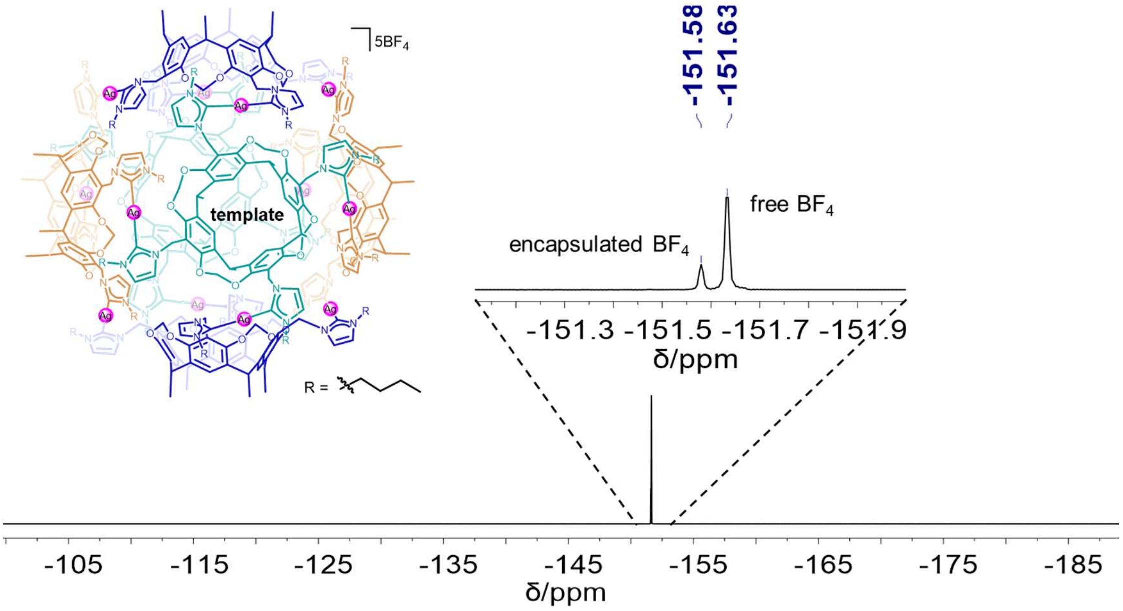

Figure S18. ^{19}F NMR spectrum (376 MHz in CD_3CN) of $[\{Ag(CH_3CN)_4(BF_4)_8\}\subset Ag_{12}(\mathbf{1})_6](BF_4)_5$.

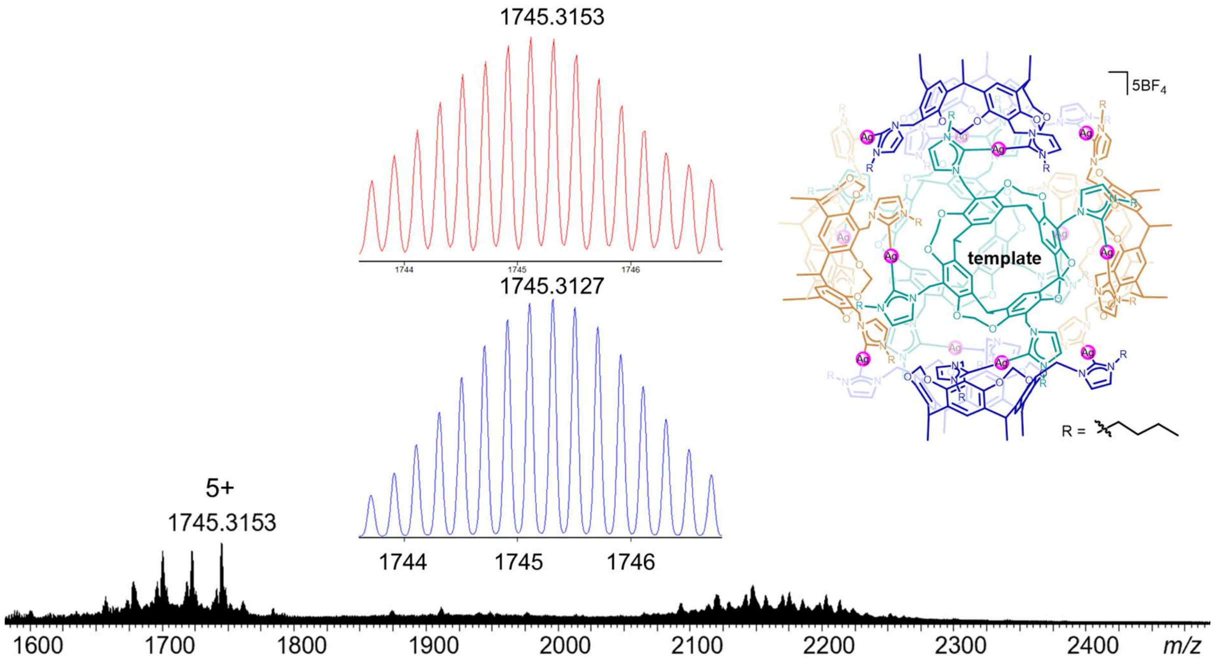

Figure S19. ESI-TOF mass spectrum of [{Ag(CH$_3$CN)$_4$(BF$_4$)$_8$}⊂Ag$_{12}$(**1**)$_6$](BF$_4$)$_5$ with isotope distribution for the 5+ cation peak (experimental in red, calculated in blue).

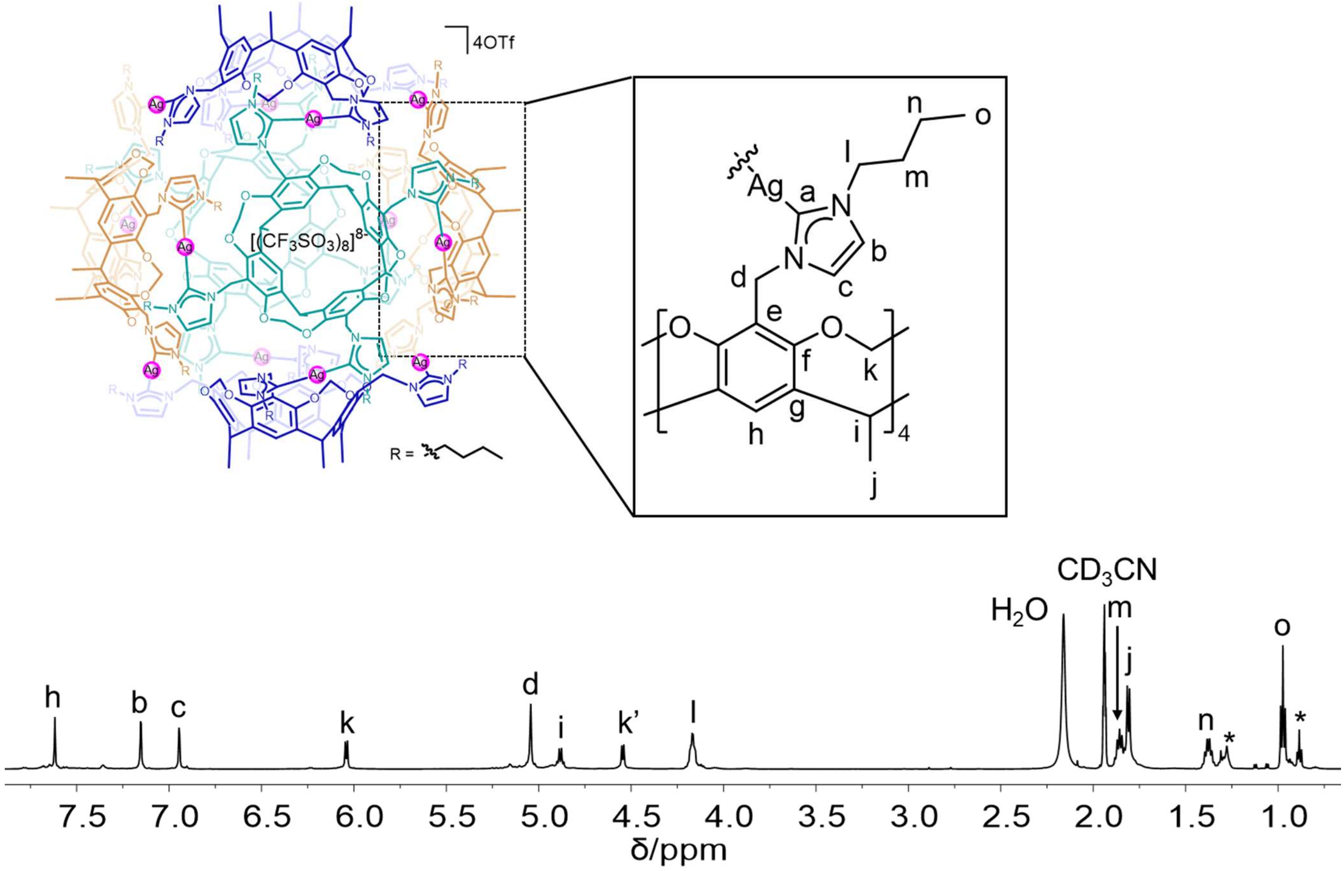

Figure S20. ^{1}H NMR spectrum (600 MHz, CD$_3$CN) of [(OTf)$_8$⊂Ag$_{12}$(**1**)$_6$](OTf)$_4$ (* = *n*-hexane).

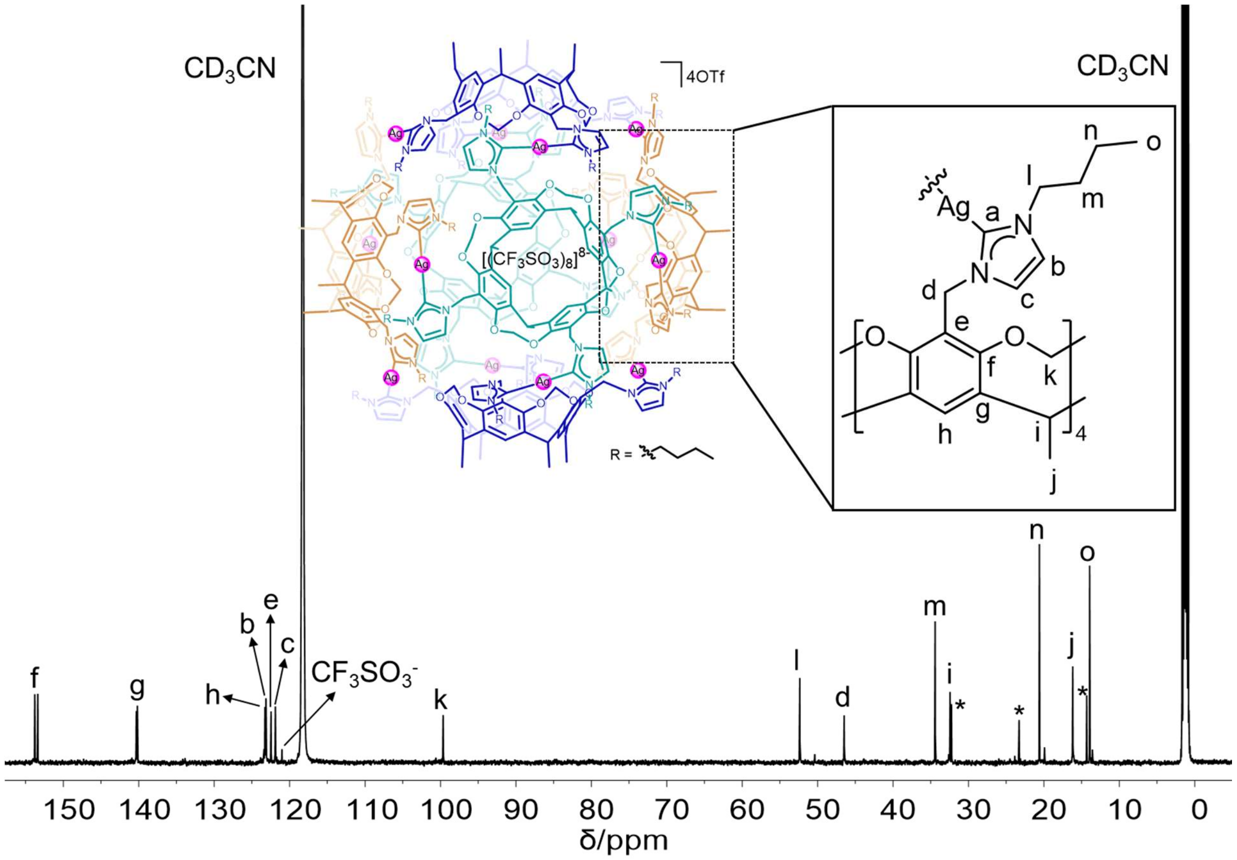

Figure S21. $^{13}C\{^1H\}$ NMR spectrum (150 MHz, CD_3CN) of $[(OTf)_8\subset Ag_{12}(\mathbf{1})_6](OTf)_4$ (* = *n*-hexane).

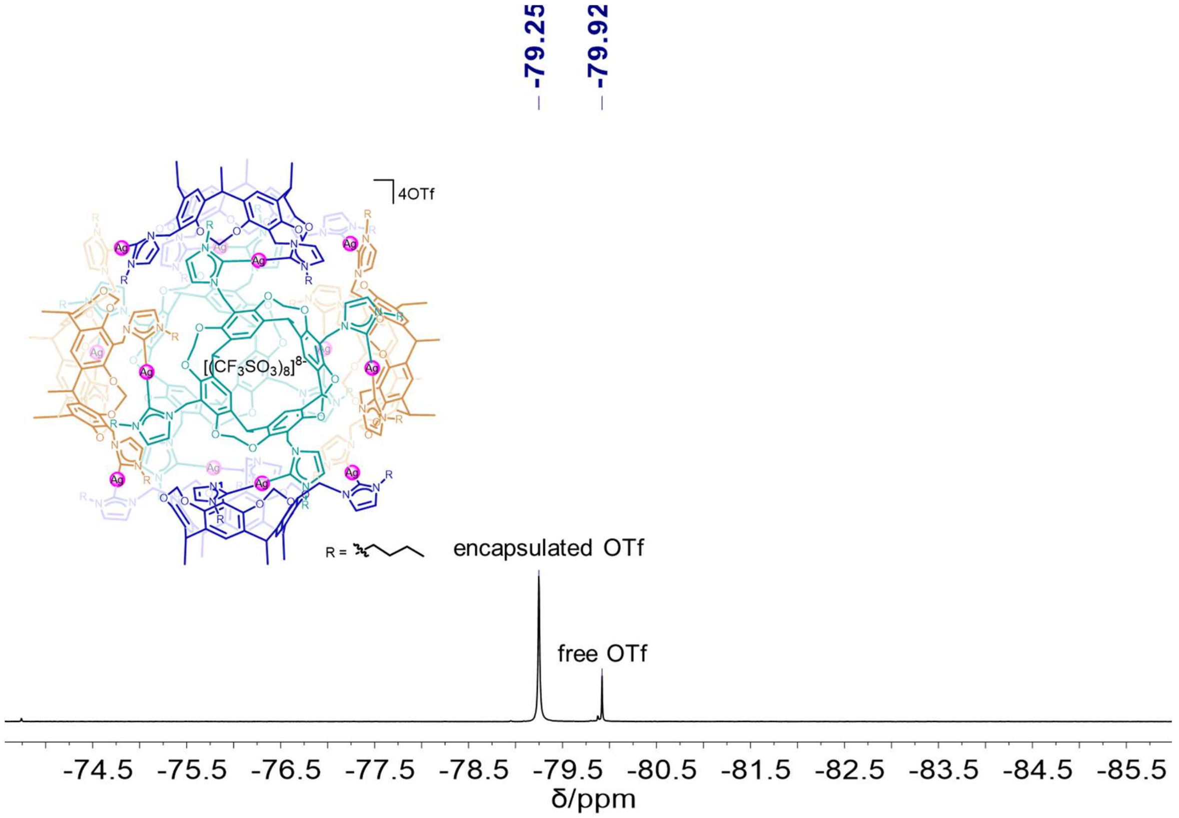

Figure S22. ^{19}F NMR spectrum (376 MHz, CD_3CN) of $[(OTf)_8\subset Ag_{12}(\mathbf{1})_6](OTf)_4$.

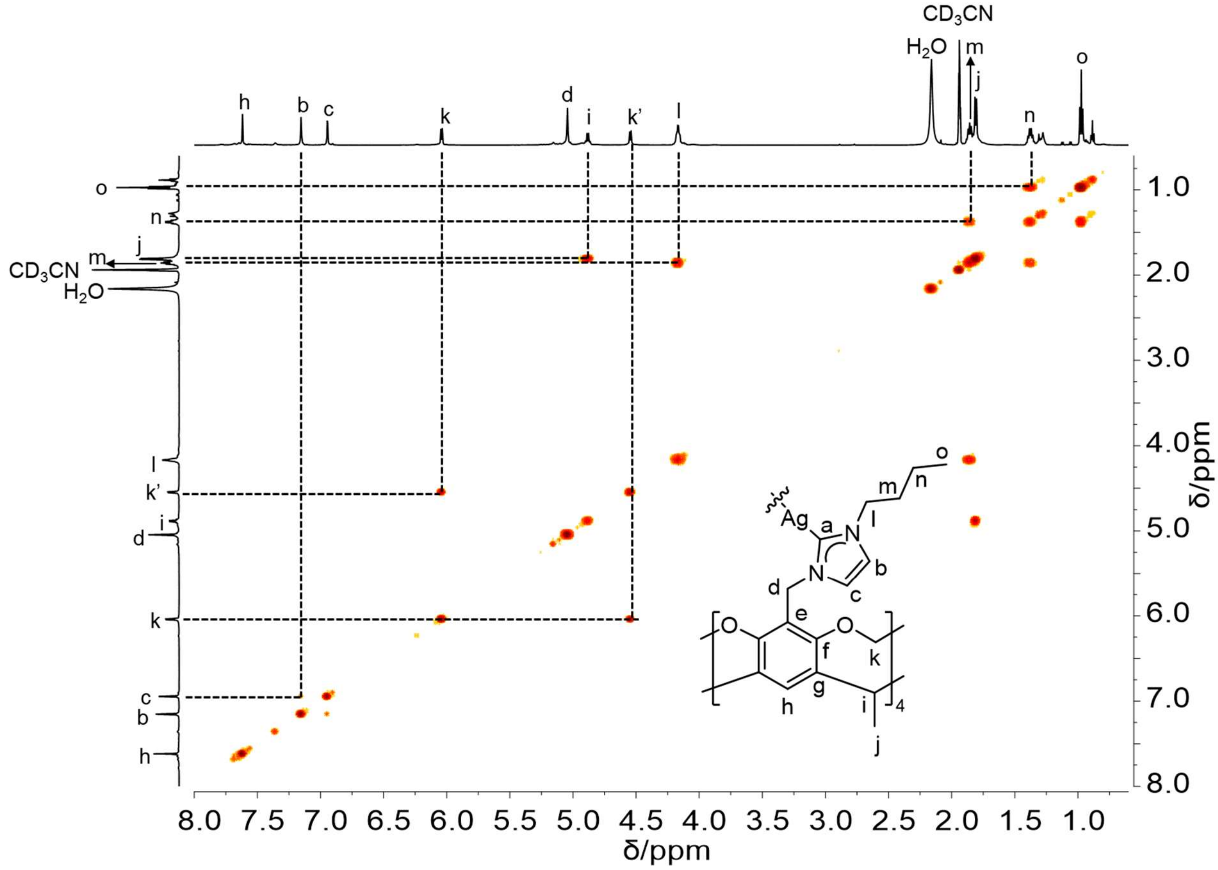

Figure S23. ^{1}H-^{1}H COSY spectrum (600 MHz, CD_3CN) of $[(OTf)_8 \subset Ag_{12}(\mathbf{1})_6](OTf)_4$.

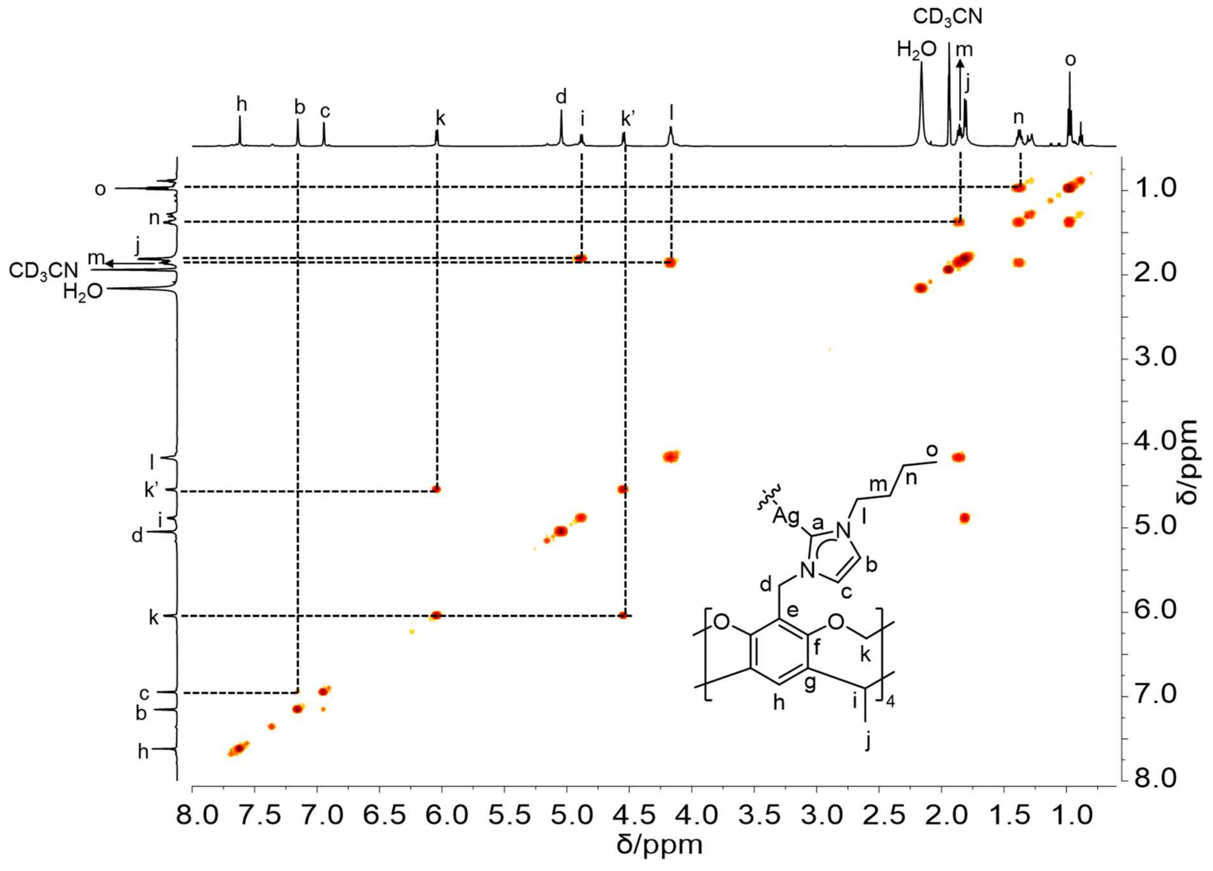

Figure S24. ^{1}H-^{13}C HSQC spectrum (600 MHz, CD_3CN) of $[(OTf)_8 \subset Ag_{12}(\mathbf{1})_6](OTf)_4$ (* = *n*-hexane).

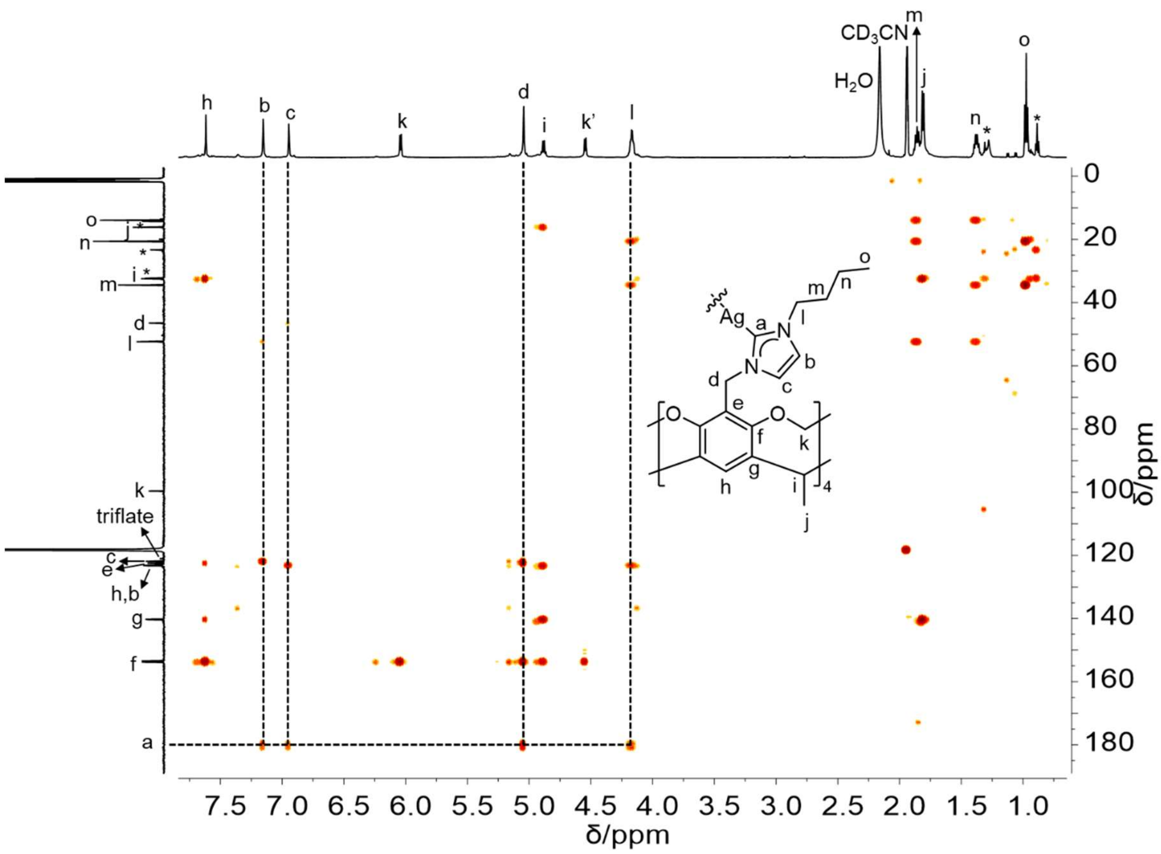

Figure S25. ^{1}H-^{13}C HMBC spectrum (600 MHz, CD_3CN) of $[(OTf)_8{\subset}Ag_{12}(\mathbf{1})_6](OTf)_4$ (* = *n*-hexane).

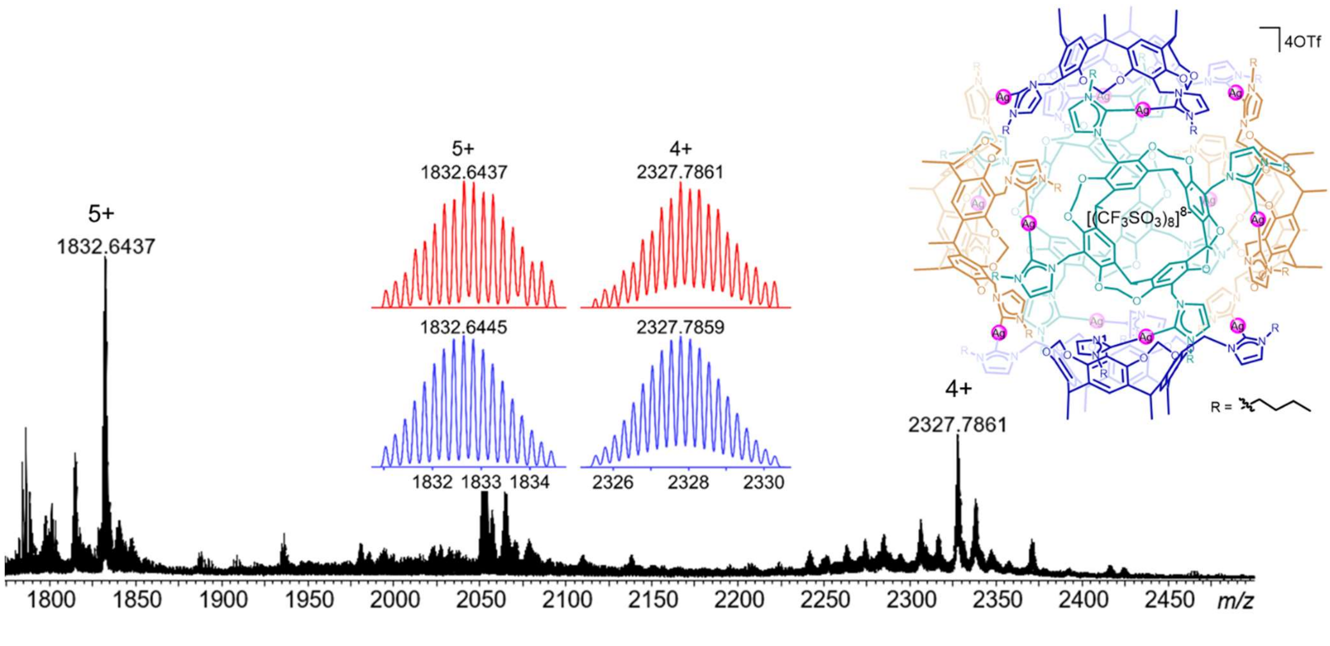

Figure S26. ESI-TOF mass spectrum of $[(OTf)_8{\subset}Ag_{12}(\mathbf{1})_6](OTf)_4$ with isotope distribution for two cation peaks (experimental in red, calculated in blue).

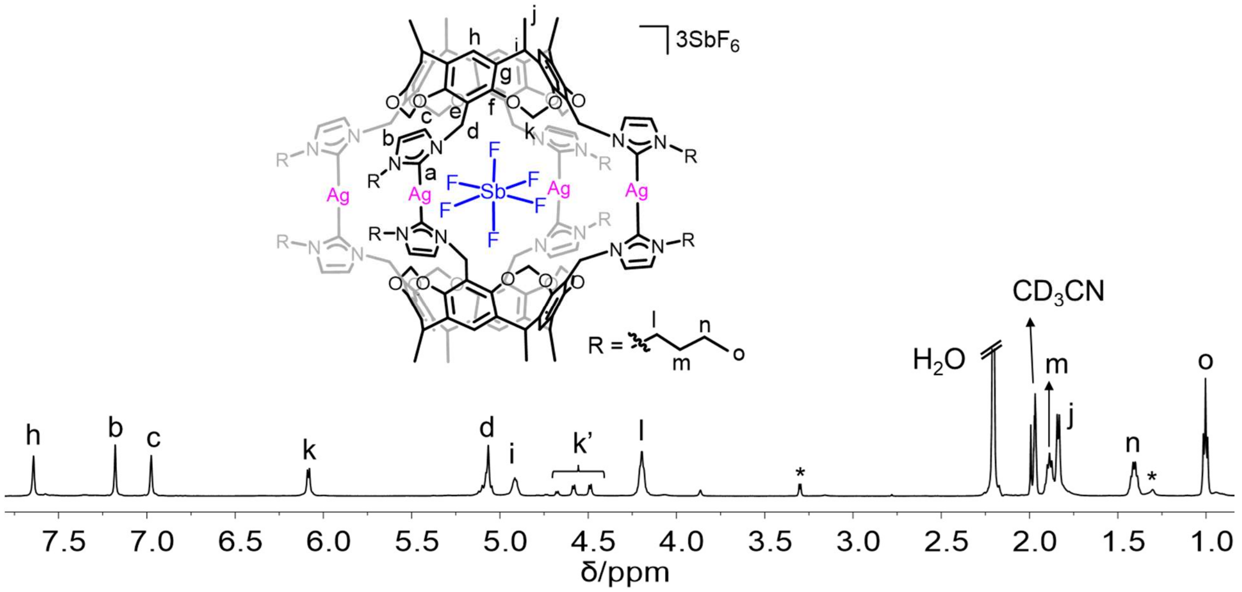

Figure S27. ^{1}H NMR spectrum (600 MHz, CD_3CN) of $[(SbF_6)\subset Ag_4(\mathbf{1})_2](SbF_6)_3$ (* = diethyl ether).

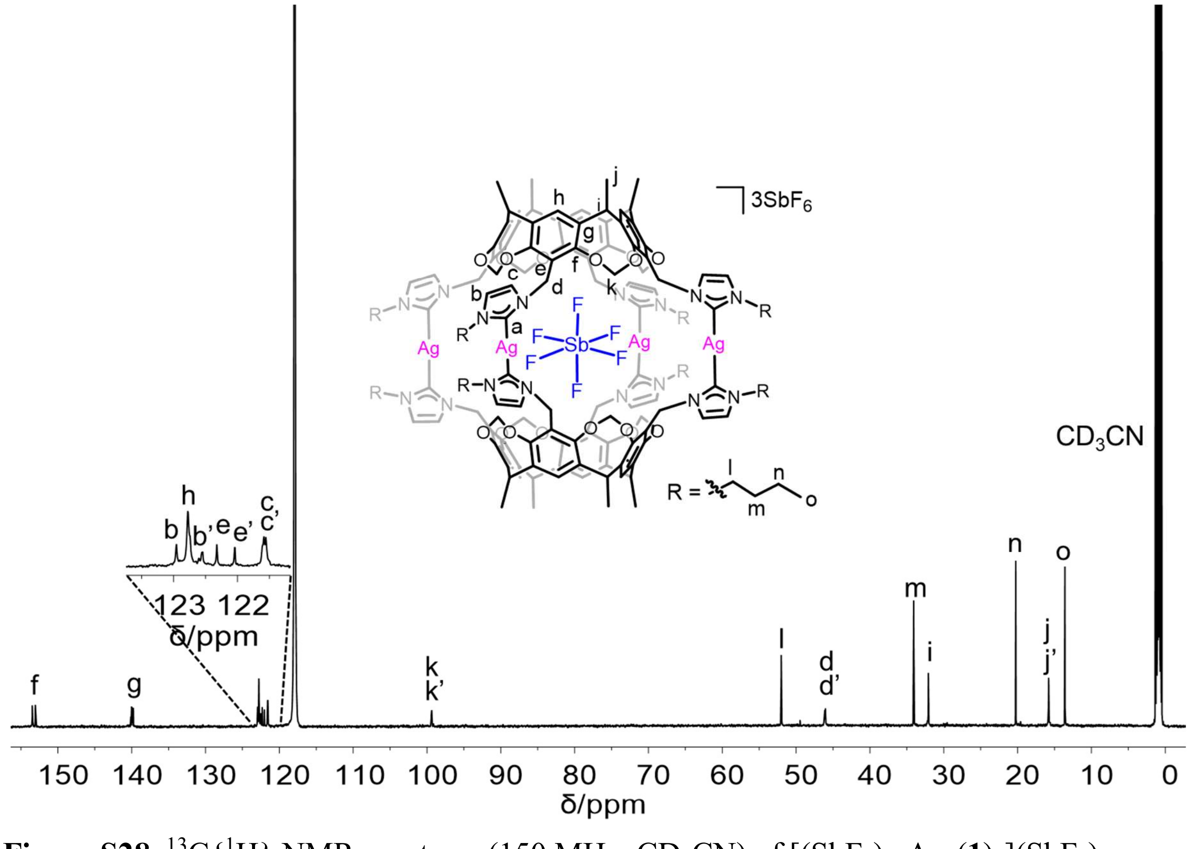

Figure S28. ^{13}C{^{1}H} NMR spectrum (150 MHz, CD_3CN) of $[(SbF_6)\subset Ag_4(\mathbf{1})_2](SbF_6)_3$.

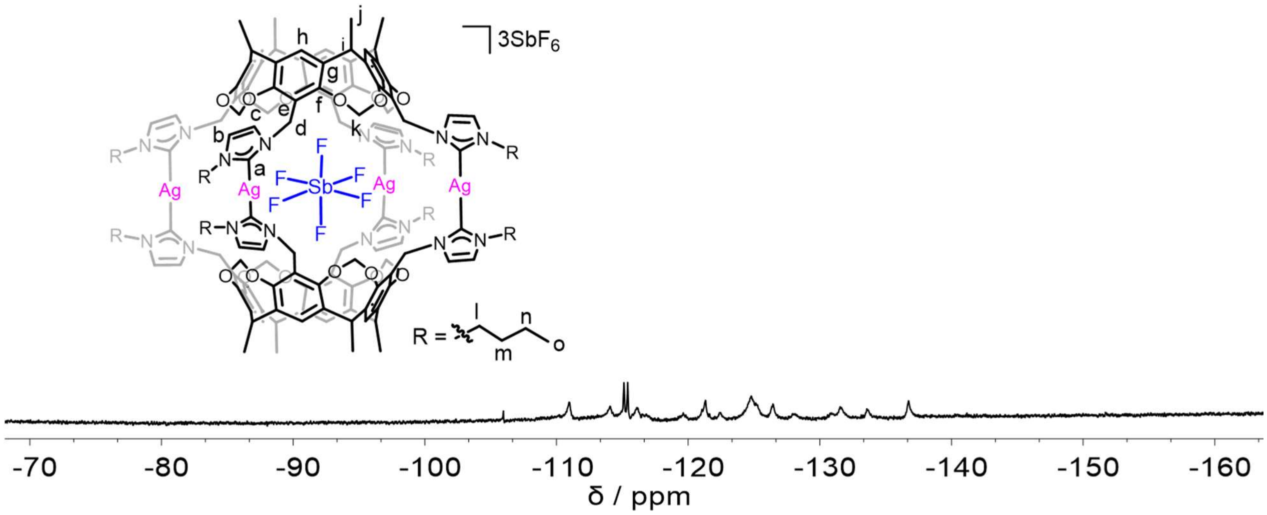

Figure S29. ^{19}F NMR spectrum (376 MHz, CD_3CN) of $[(SbF_6)\subset Ag_4(\mathbf{1})_2](SbF_6)_3$.

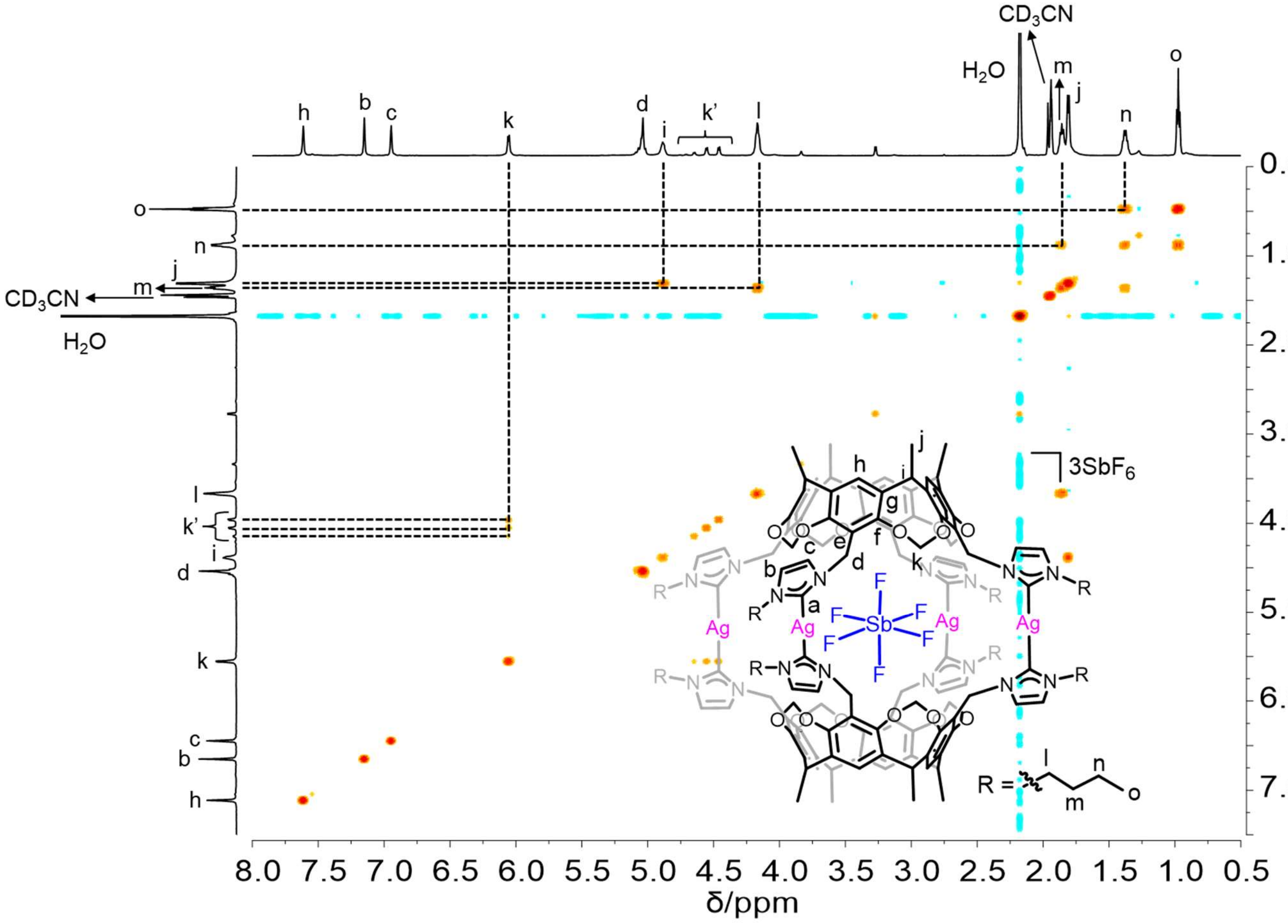

Figure S30. ^{1}H-^{1}H COSY spectrum (600 MHz, CD_3CN) of $[(SbF_6)\subset Ag_4(\mathbf{1})_2](SbF_6)_3$.

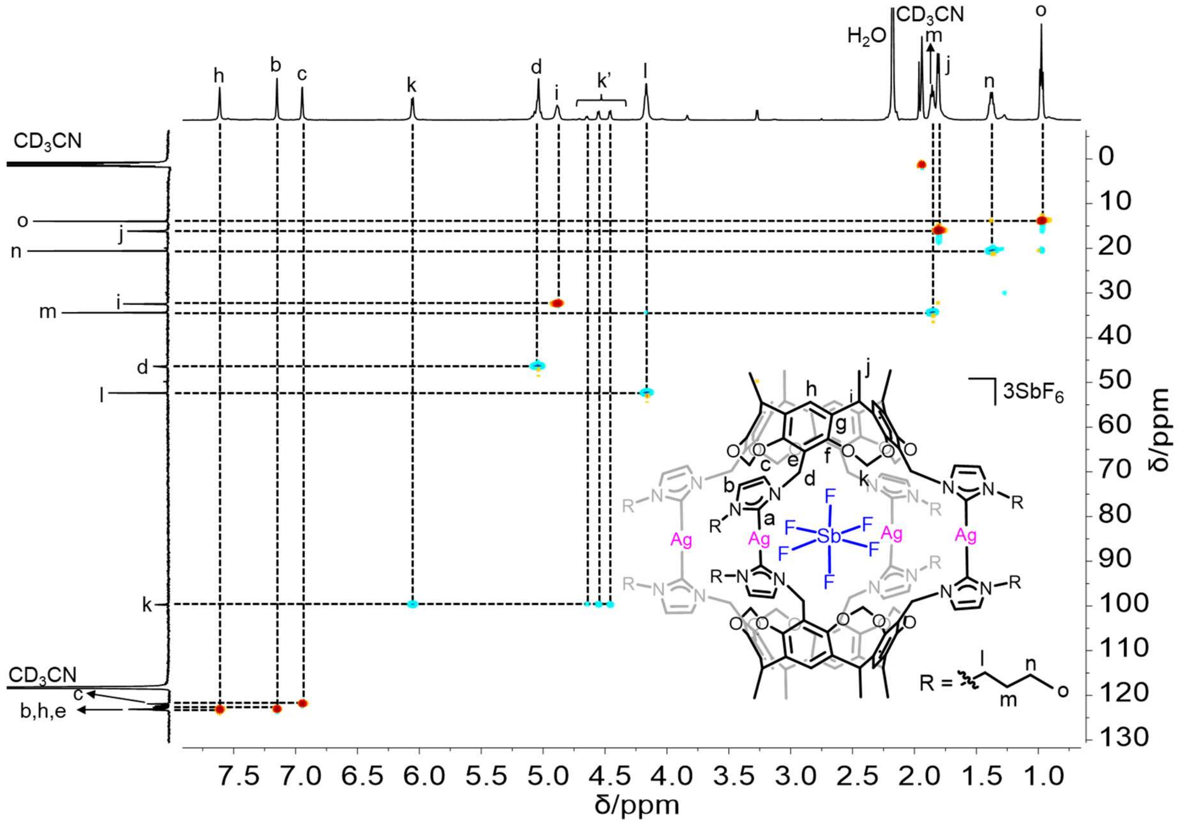

Figure S31. ^{1}H-^{13}C HSQC spectrum (600 MHz, CD_3CN) of $[(SbF_6)\subset Ag_4(\mathbf{1})_2](SbF_6)_3$.

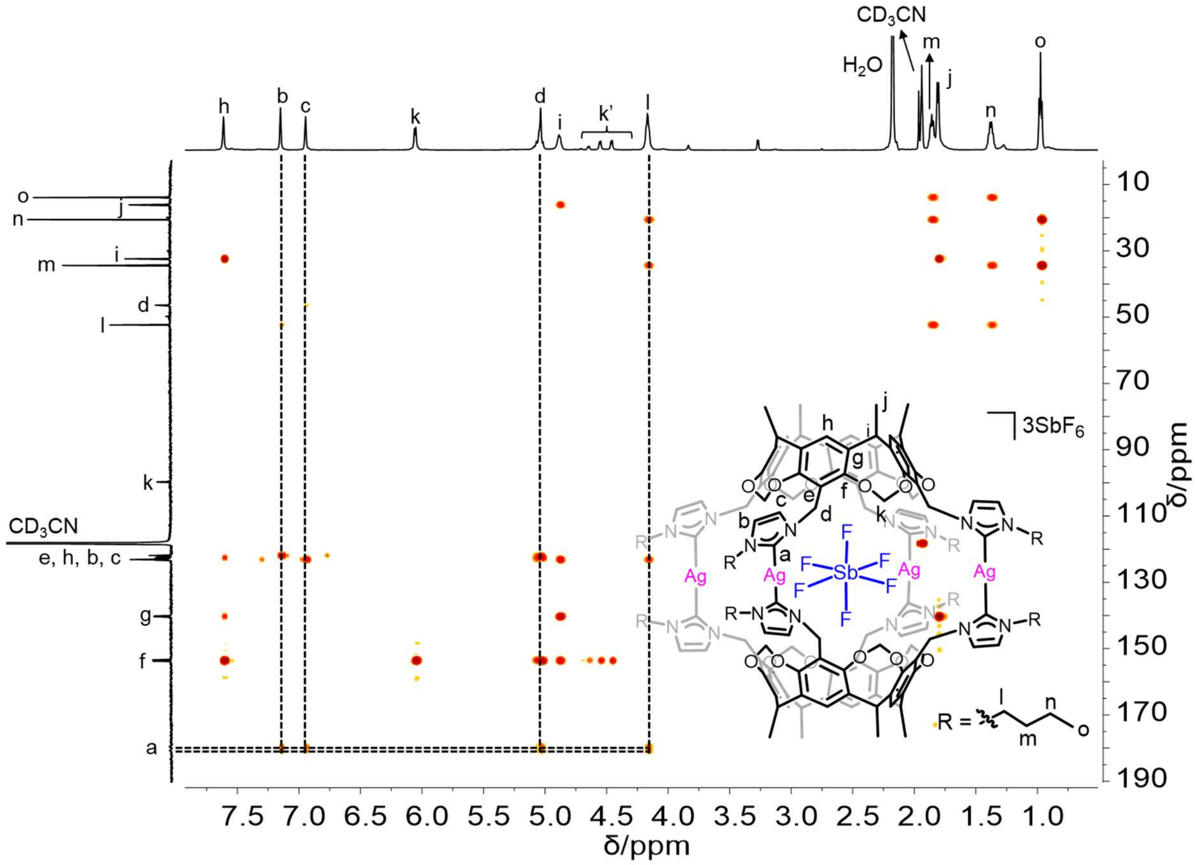

Figure S32. ^{1}H-^{13}C HMBC spectrum (600 MHz, CD_3CN) of complex $[(SbF_6)\subset Ag_4(\mathbf{1})_2](SbF_6)_3$.

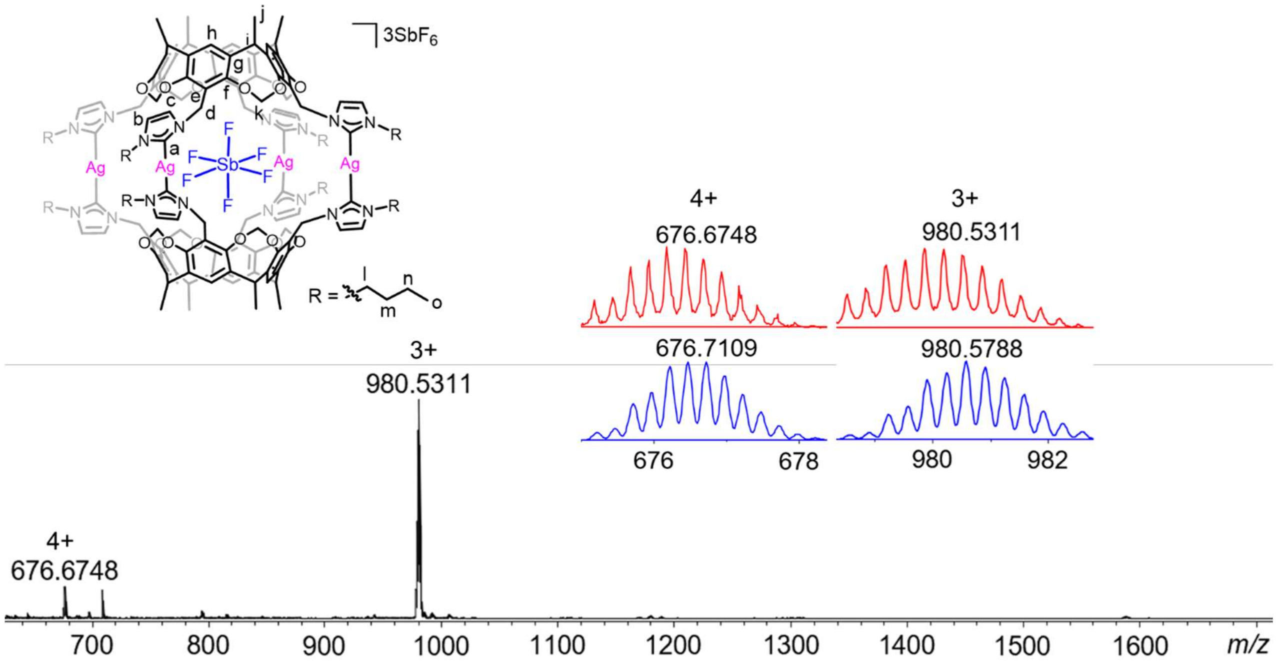

Figure S33. ESI-TOF mass spectrum of $[(SbF_6)\subset Ag_4(\mathbf{1})_2](SbF_6)_3$ with isotope distribution for two cation peaks (experimental in red, calculated in blue).

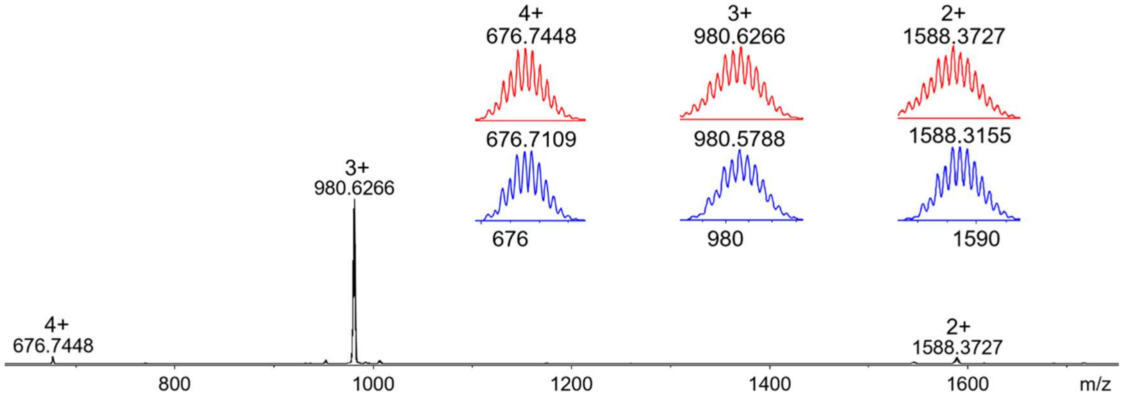

Figure S34. ESI-TOF mass spectrum of $[(SbF_6)\subset Ag_4(\mathbf{1})_2](SbF_6)_3$ after addition of $AgBF_4$. Insets showing three cationic peaks for $[(SbF_6)\subset Ag_4(\mathbf{1})_2](SbF_6)_3$ (experimental in red, calculated in blue). No peaks were found for any species other than$[(SbF_6)\subset Ag_4(\mathbf{1})_2](SbF_6)_3$, indicating that this species is stable and does not rearrange in the presence of BF_4^- anions.

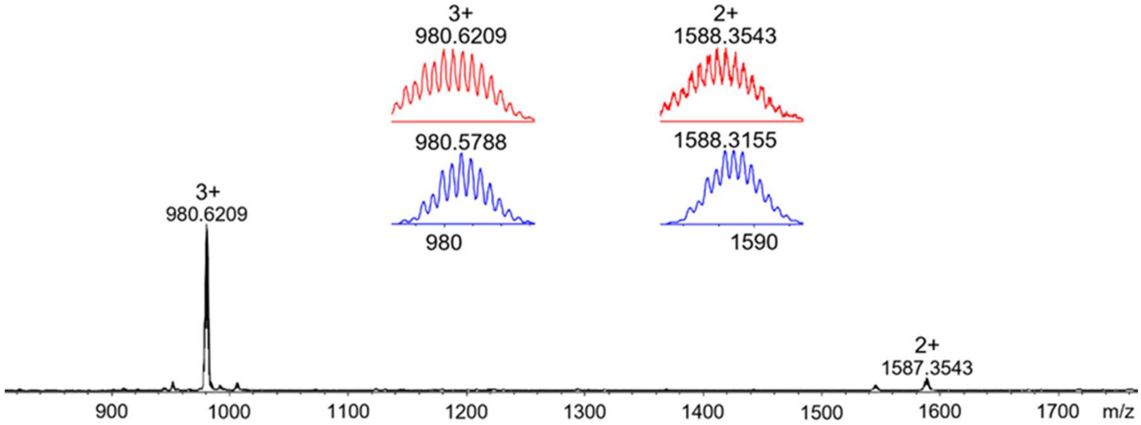

Figure S35. ESI-TOF mass spectrum of $[(OTf)_8\subset Ag_{12}(\mathbf{1})_6](OTf)_4$ after addition of $AgSbF_6$. Insets showing three cationic peaks for $[(SbF_6)\subset Ag_4(\mathbf{1})_2](SbF_6)_3$ (experimental in red, calculated in blue). No peaks for $[(OTf)_8)\subset Ag_{12}(\mathbf{1})_6](OTf)_4$ were found anymore. Apparently, transformation of $[(OTf)_8)\subset Ag_{12}(\mathbf{1})_6](OTf)_4$ into $[(SbF_6)\subset Ag_4(\mathbf{1})_2](SbF_6)_3$ happened upon addition of $AgSbF_6$.

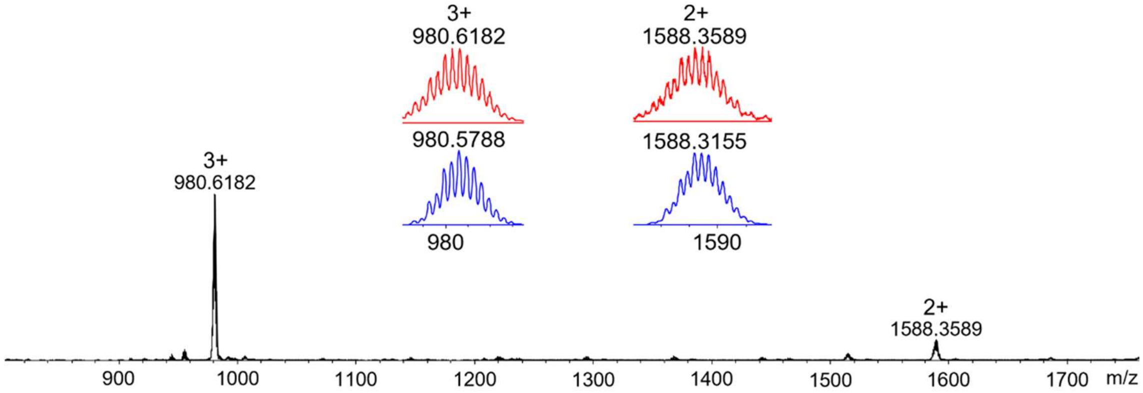

Figure S36. ESI-TOF mass spectrum of $[\{Ag(CH_3CN)_4(BF_4)_8\}\subset Ag_{12}(\mathbf{1})_6](BF_4)_5$ after addition of $AgSbF_6$. Insets showing three cationic peaks for $[(SbF_6)\subset Ag_4(\mathbf{1})_2](SbF_6)_3$ (experimental in red, calculated in blue). No peaks for $[\{Ag(CH_3CN)_4(BF_4)_8\}\subset Ag_{12}(\mathbf{1})_6](BF_4)_5$ were found anymore. Apparently, transformation of $[\{Ag(CH_3CN)_4(BF_4)_8\}\subset Ag_{12}(\mathbf{1})_6](BF_4)_5$ into $[(SbF_6)\subset Ag_4(\mathbf{1})_2](SbF_6)_3$ happened upon addition of $AgSbF_6$.

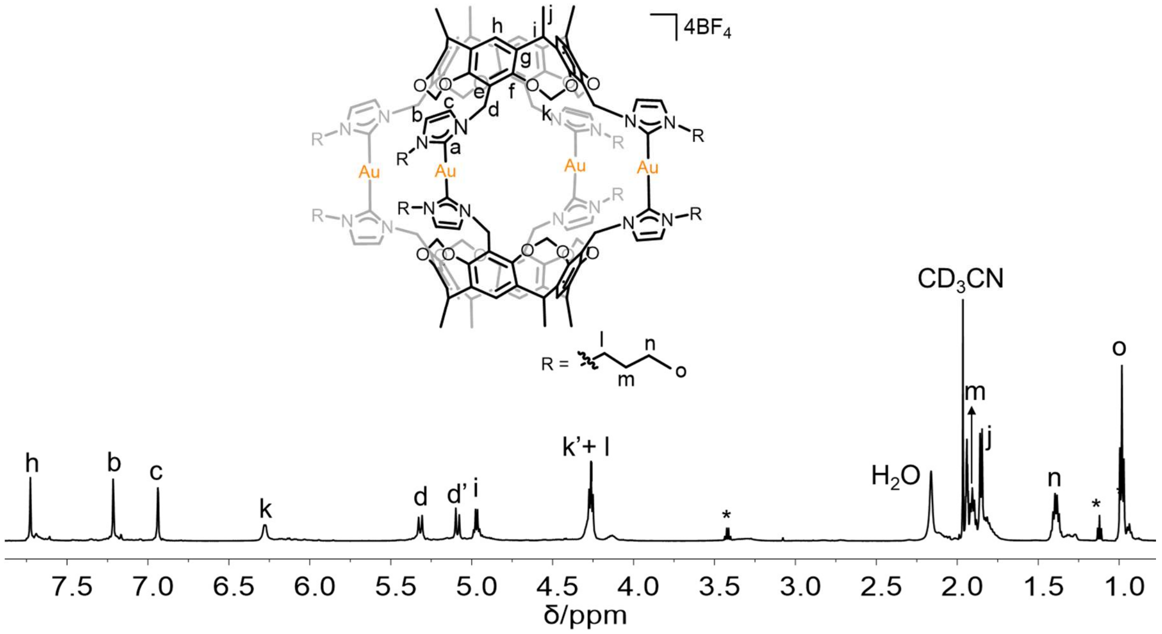

Figure S37. ^{1}H NMR spectrum (600 MHz, CD_3CN) of $[Au_4(\mathbf{1})_2](BF_4)_4$ (* = diethyl ether).

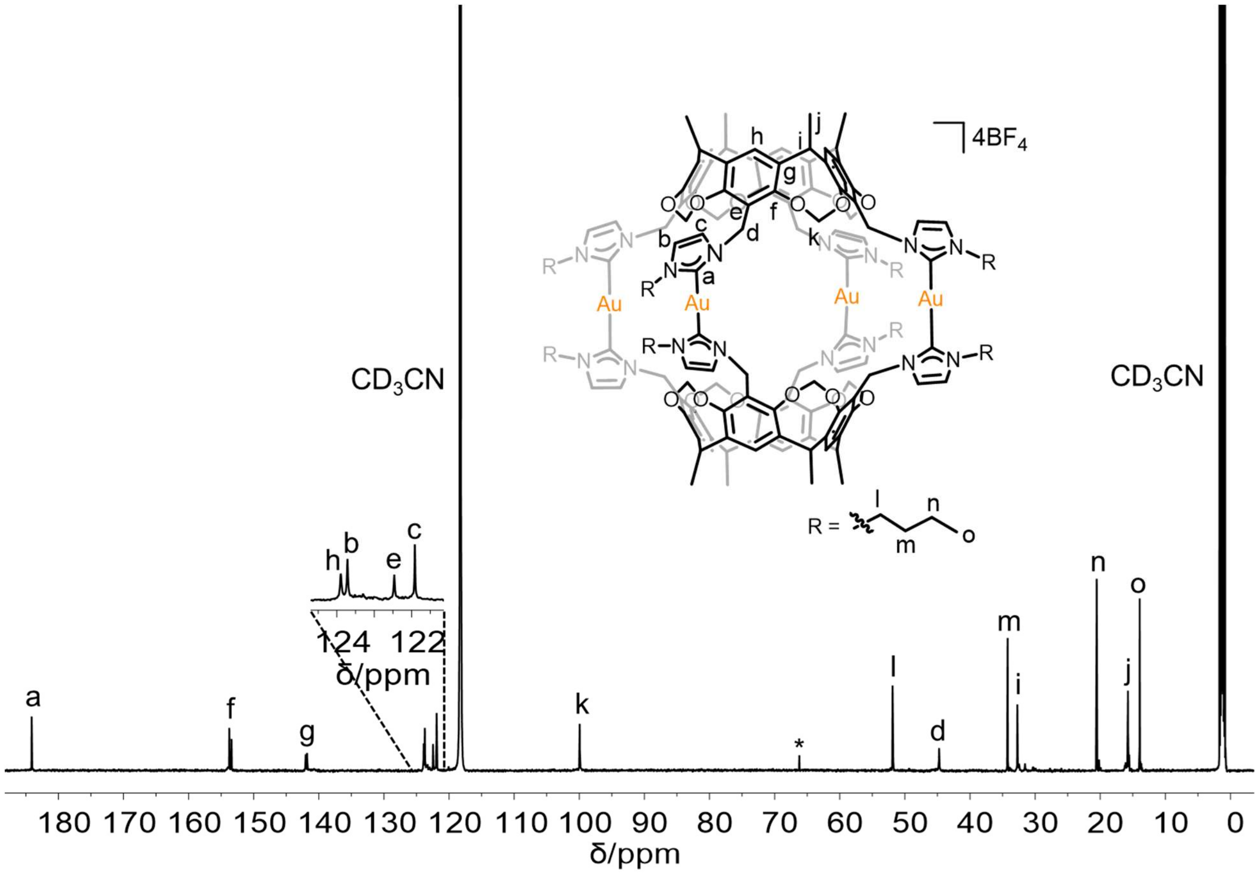

Figure S38. $^{13}C\{^{1}H\}$ NMR spectrum (150 MHz, CD_3CN) of $[Au_4(\mathbf{1})_2](BF_4)_4$ (* = diethyl ether).

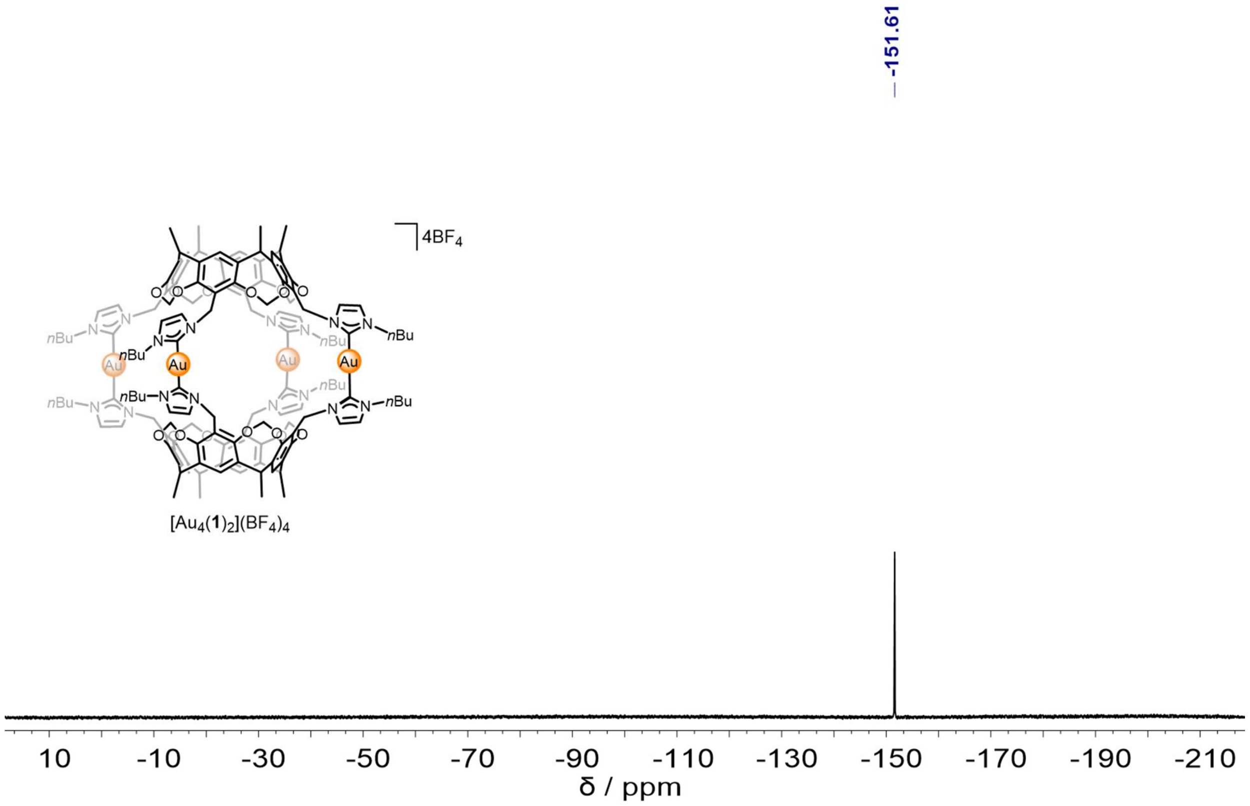

Figure S39. ^{19}F NMR spectrum (376 MHz, CD_3CN) of $[Au_4(\mathbf{1})_2](BF_4)_4$.

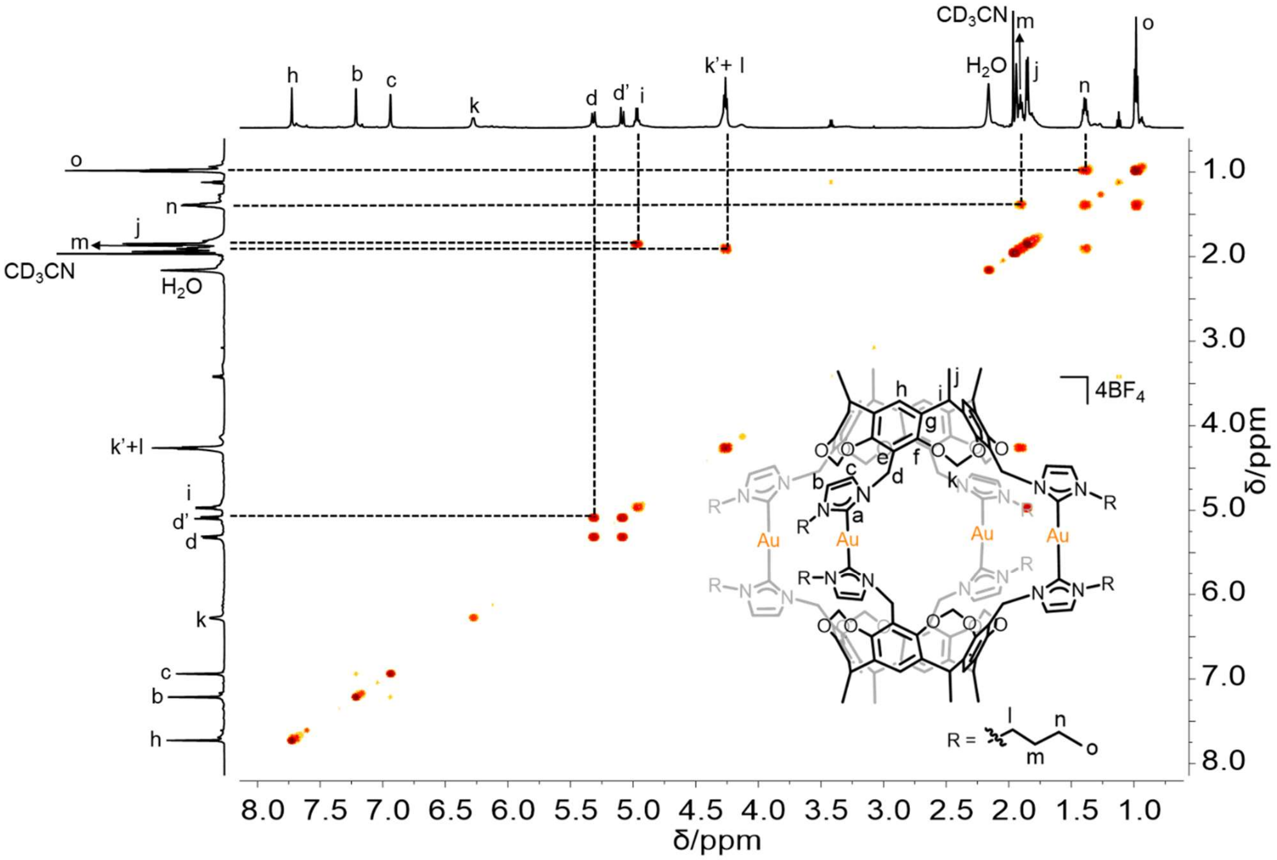

Figure S40. ^{1}H-^{1}H COSY spectrum (600 MHz, CD_3CN) of $[Au_4(\mathbf{1})_2](BF_4)_4$.

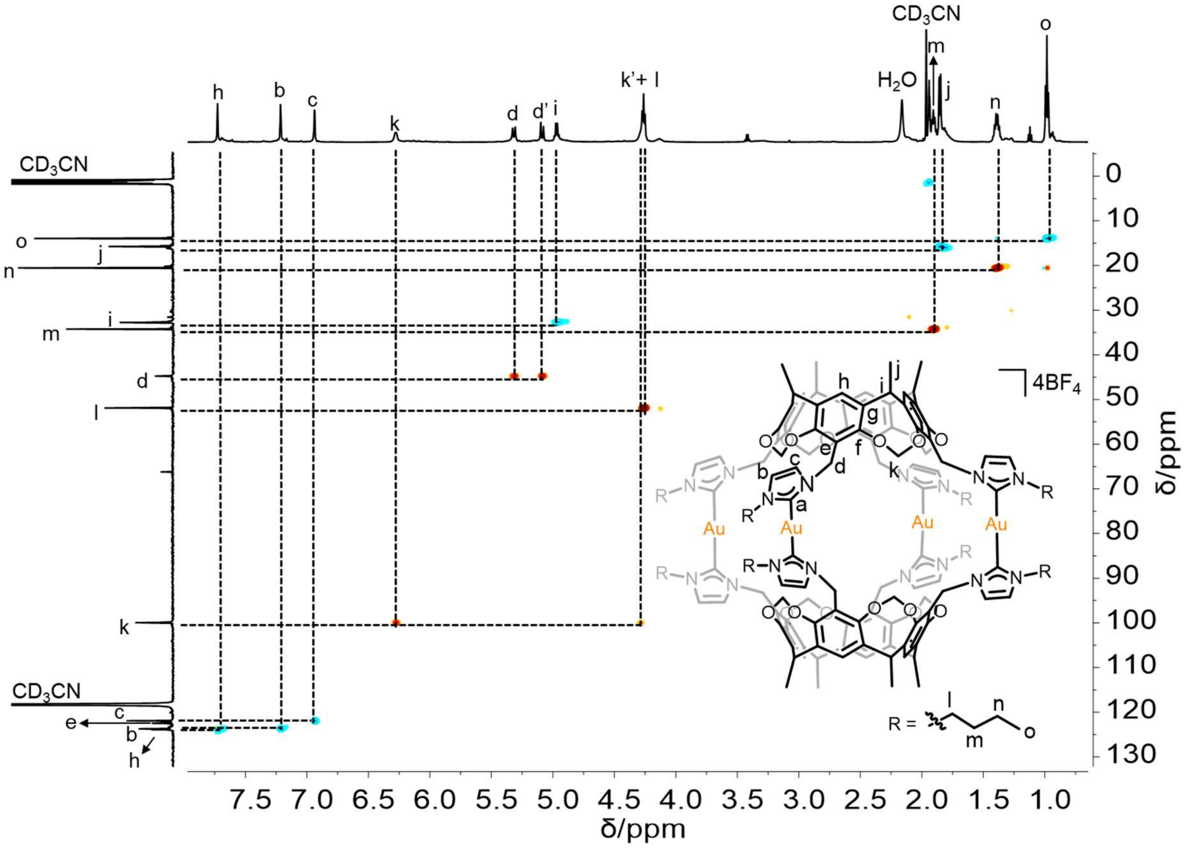

Figure S41. ^{1}H-^{13}C HSQC spectrum (600 MHz, CD_3CN) of $[Au_4(\mathbf{1})_2](BF_4)_4$.

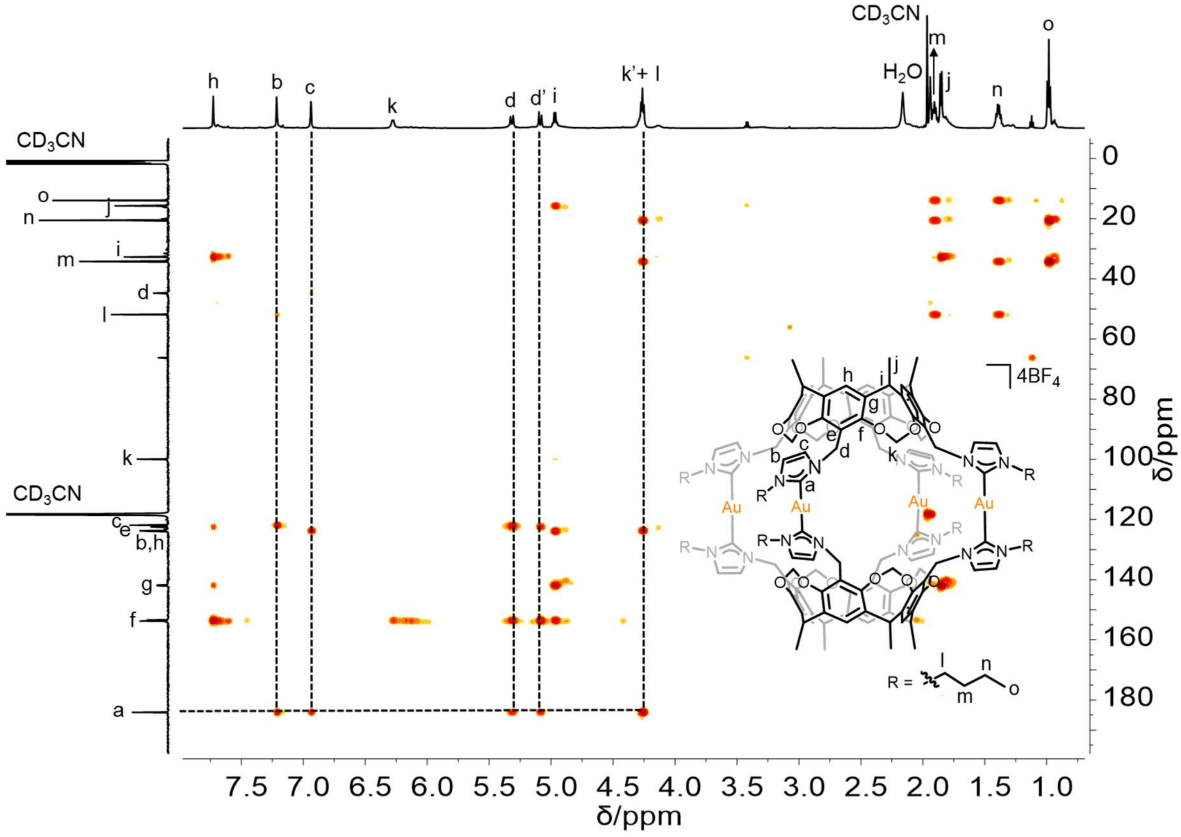

Figure S42. ^{1}H-^{13}C HMBC spectrum (600 MHz, CD_3CN) of $[Au_4(\mathbf{1})_2](BF_4)_4$.

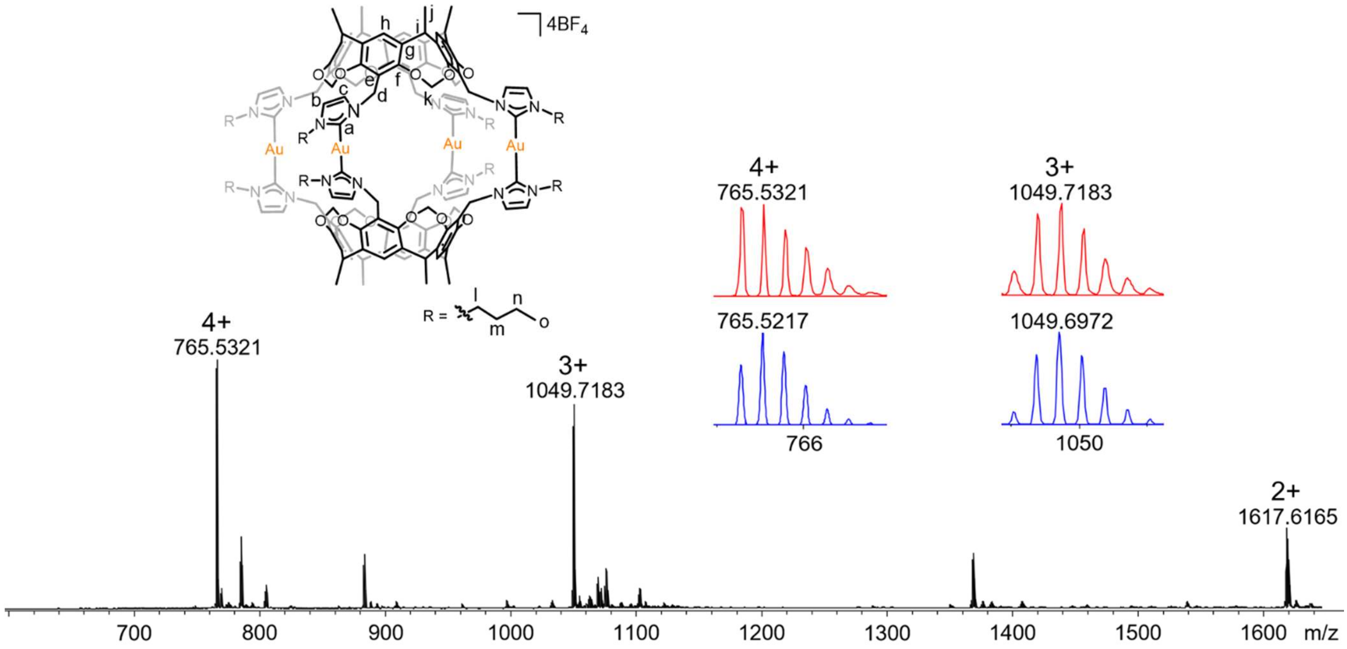

Figure S43. ESI-TOF mass spectrum of $[Au_4(\mathbf{1})_2](BF_4)_4$ with isotope distribution for two cation peaks (experimental in red, calculated in blue).

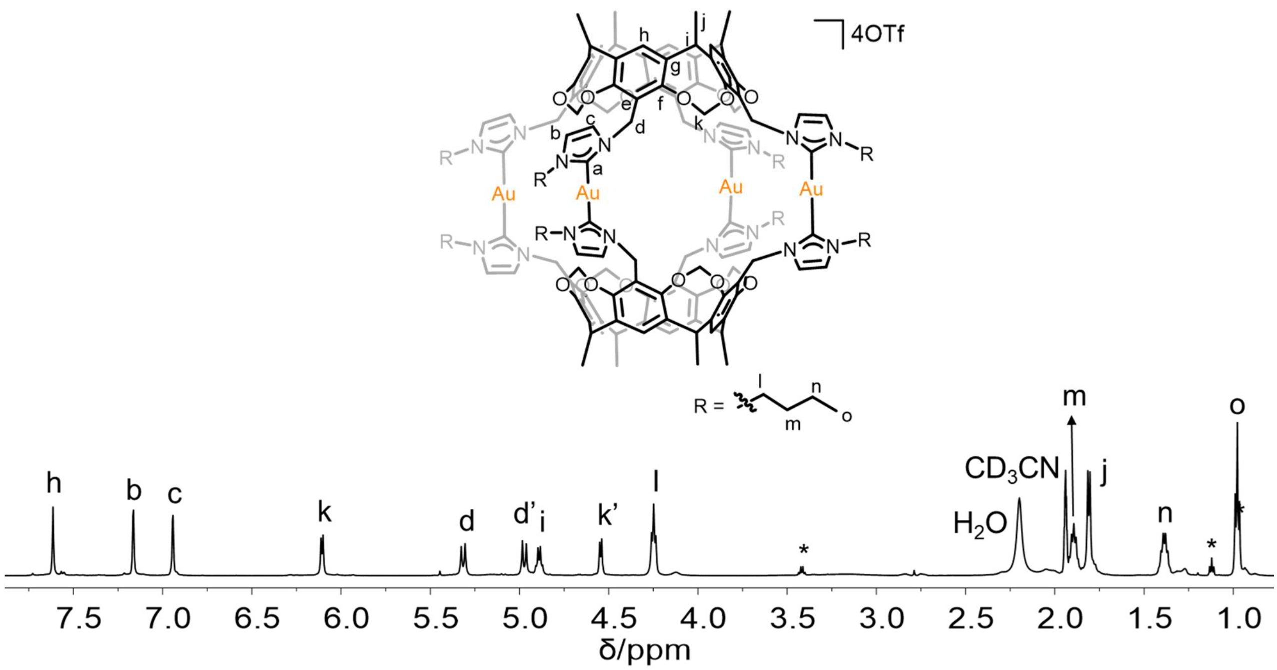

Figure S44. ^{1}H NMR spectrum (600 MHz, CD_3CN) of $[Au_4(\mathbf{1})_2](OTf)_4$ (* = diethyl ether).

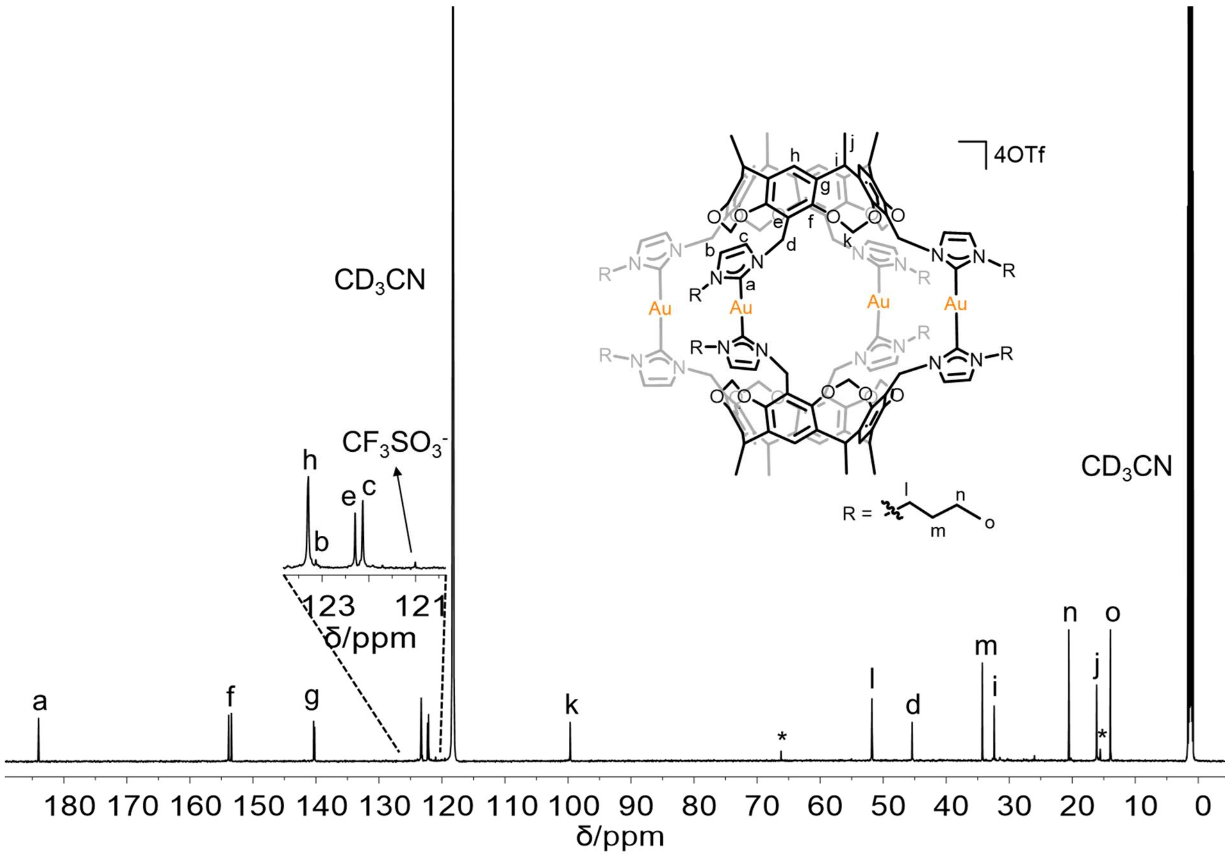

Figure S45. $^{13}C\{^{1}H\}$ NMR spectrum (150 MHz, CD_3CN) of $[Au_4(\mathbf{1})_2](OTf)_4$ (* = diethyl ether).

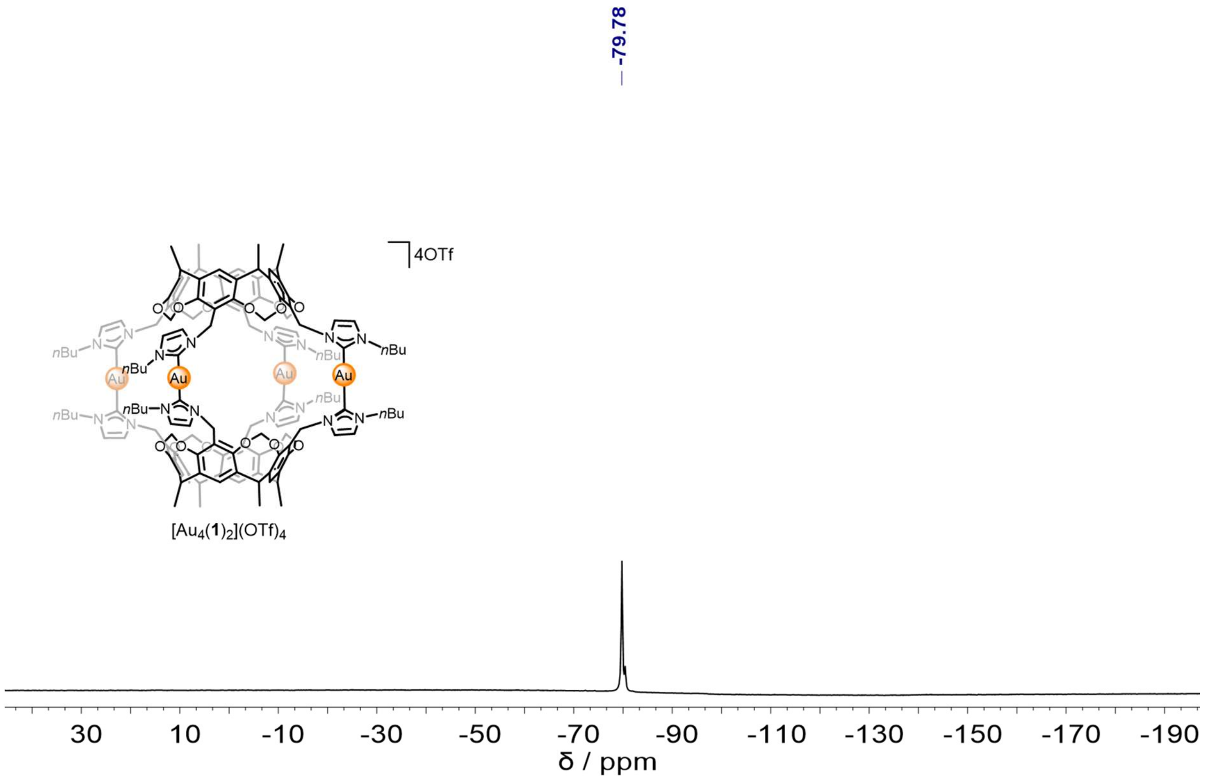

Figure S46. ^{19}F NMR spectrum (376 MHz, CD_3CN) of $[Au_4(\mathbf{1})_2](OTf)_4$.

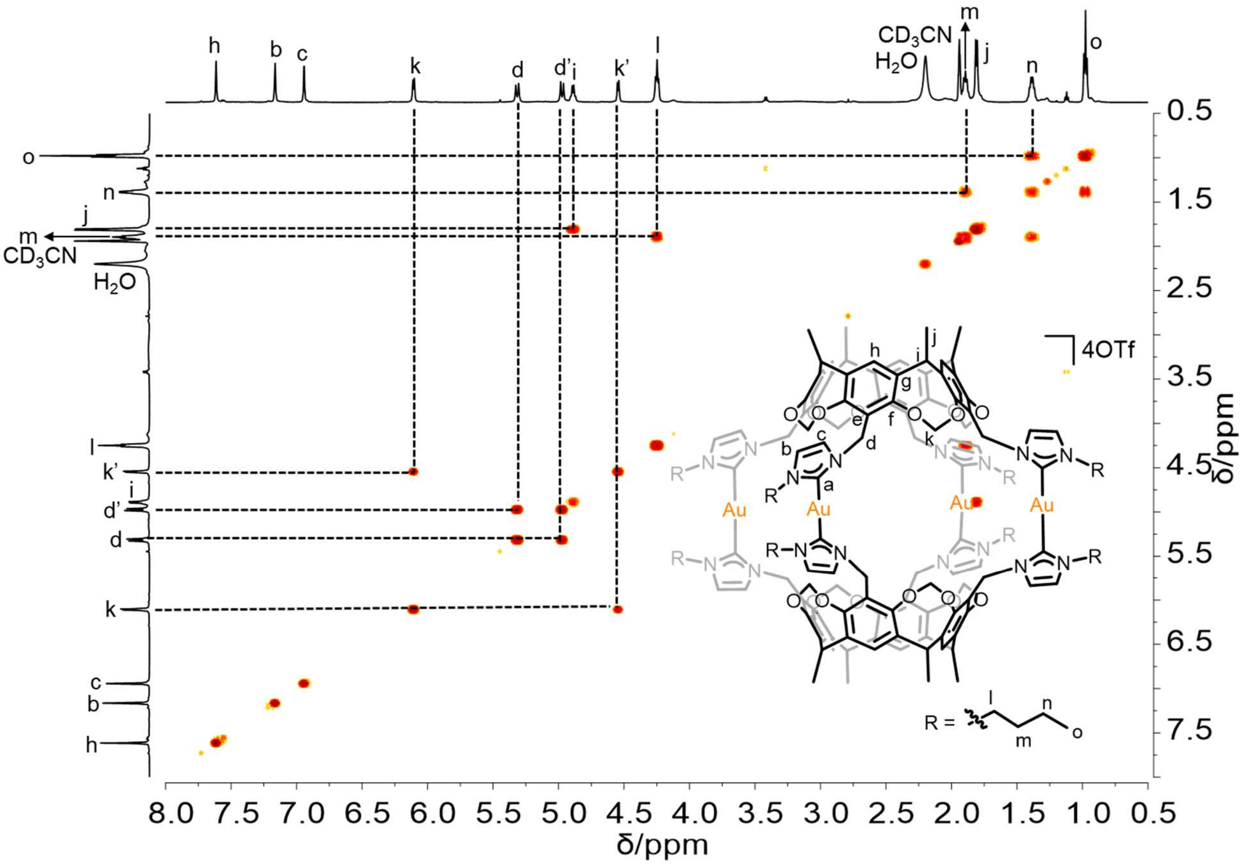

Figure S47. ^{1}H-^{1}H COSY spectrum (600 MHz, CD_3CN) of [Au_4(**1**)$_2$](OTf)$_4$.

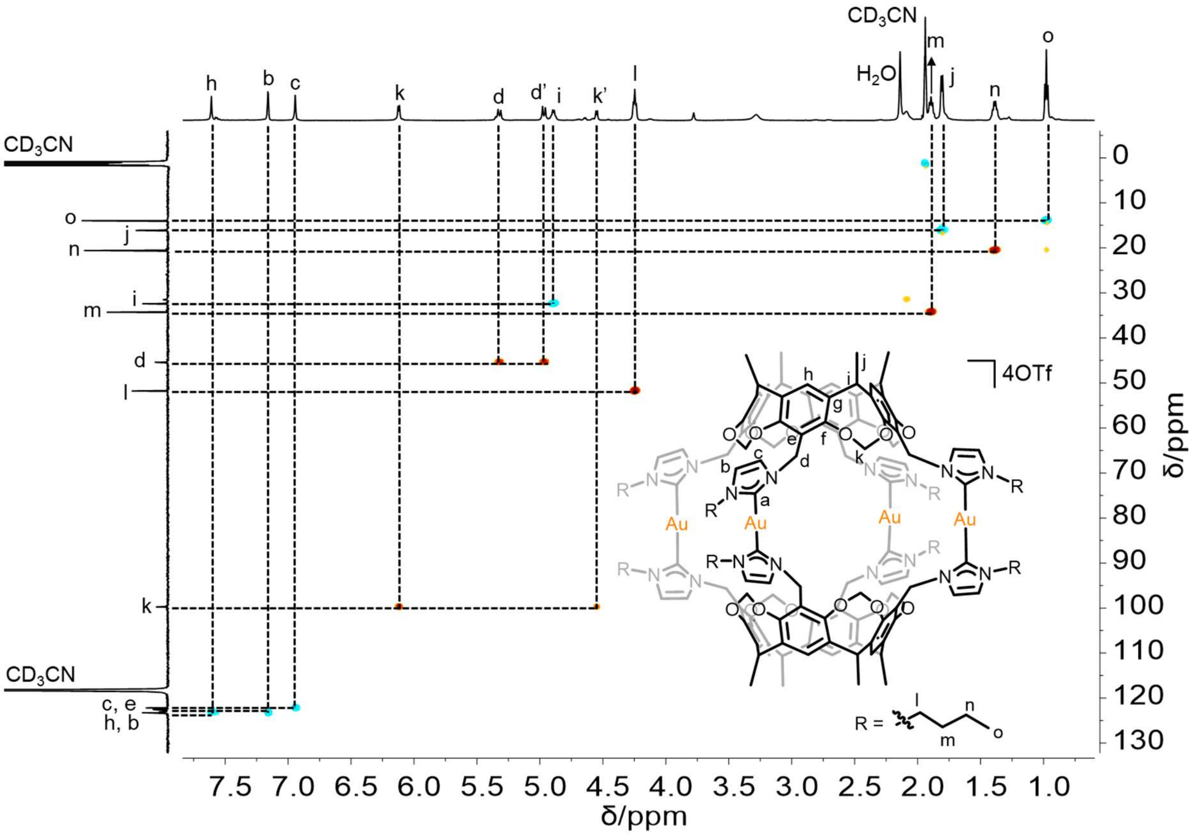

Figure S48. ^{1}H-^{13}C HSQC spectrum (600 MHz, CD_3CN) of [Au_4(**1**)$_2$](OTf)$_4$.

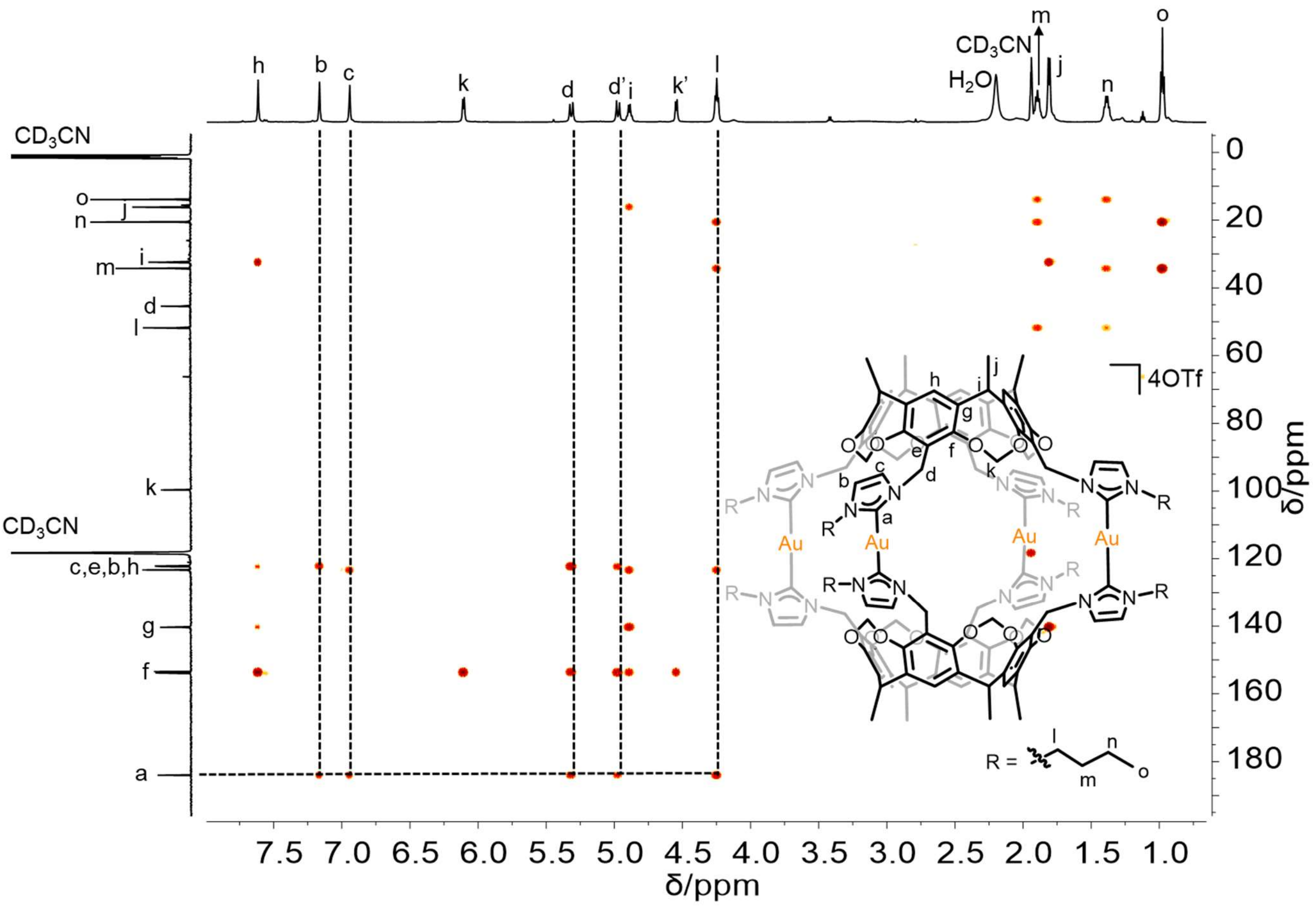

Figure S49. ^{1}H-^{13}C HMBC spectrum (600 MHz, CD_3CN) of $[Au_4(\mathbf{1})_2](OTf)_4$.

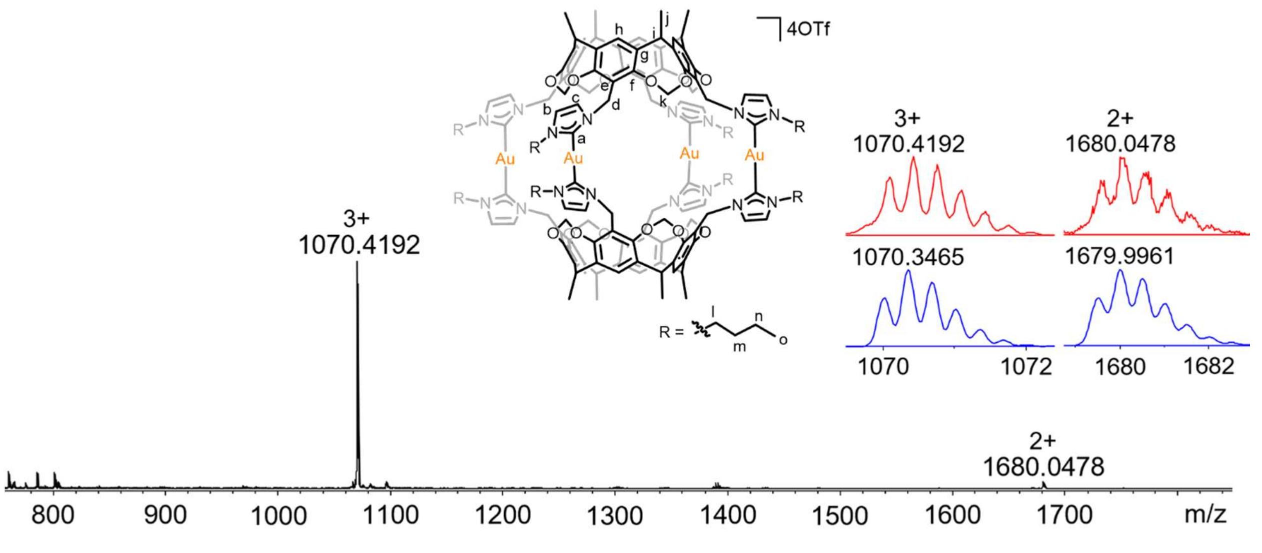

Figure S50. ESI-TOF mass spectrum of $[Au_4(\mathbf{1})_2](OTf)_4$ with isotope distribution for two cation peaks (experimental in red, calculated in blue).

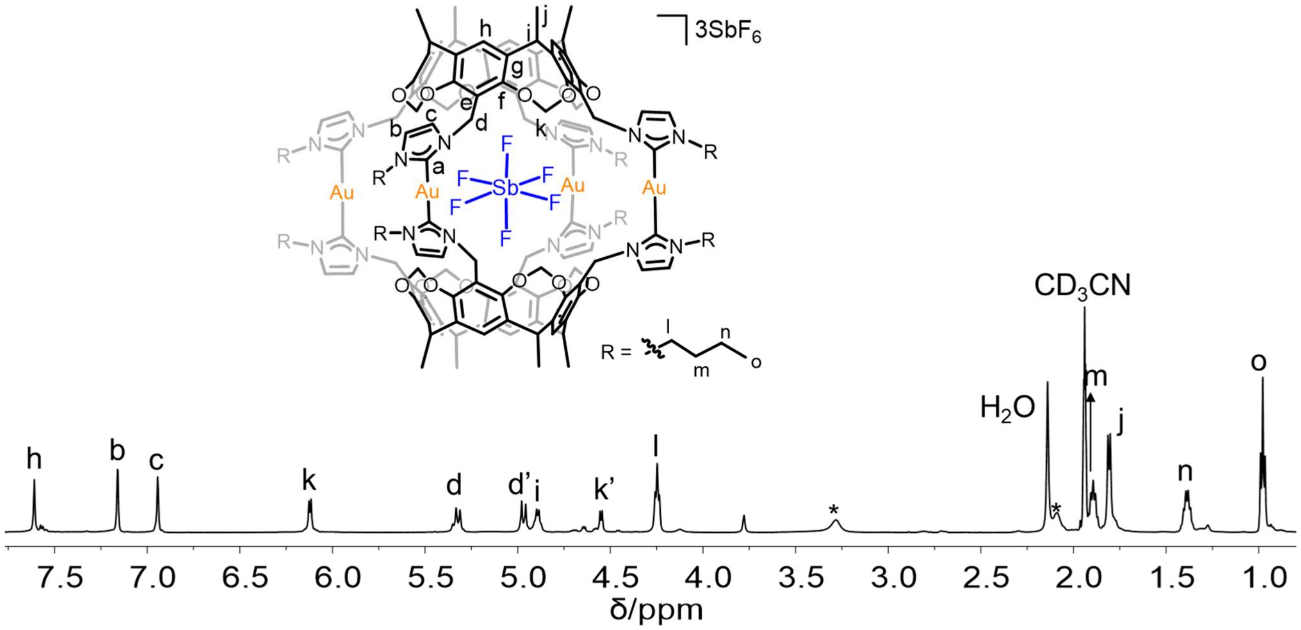

Figure S51. ^{1}H NMR spectrum (600 MHz, CD_3CN) of $[(SbF_6)\subset Au_4(\mathbf{1})_2](SbF_6)_3$ (* = residual THT).

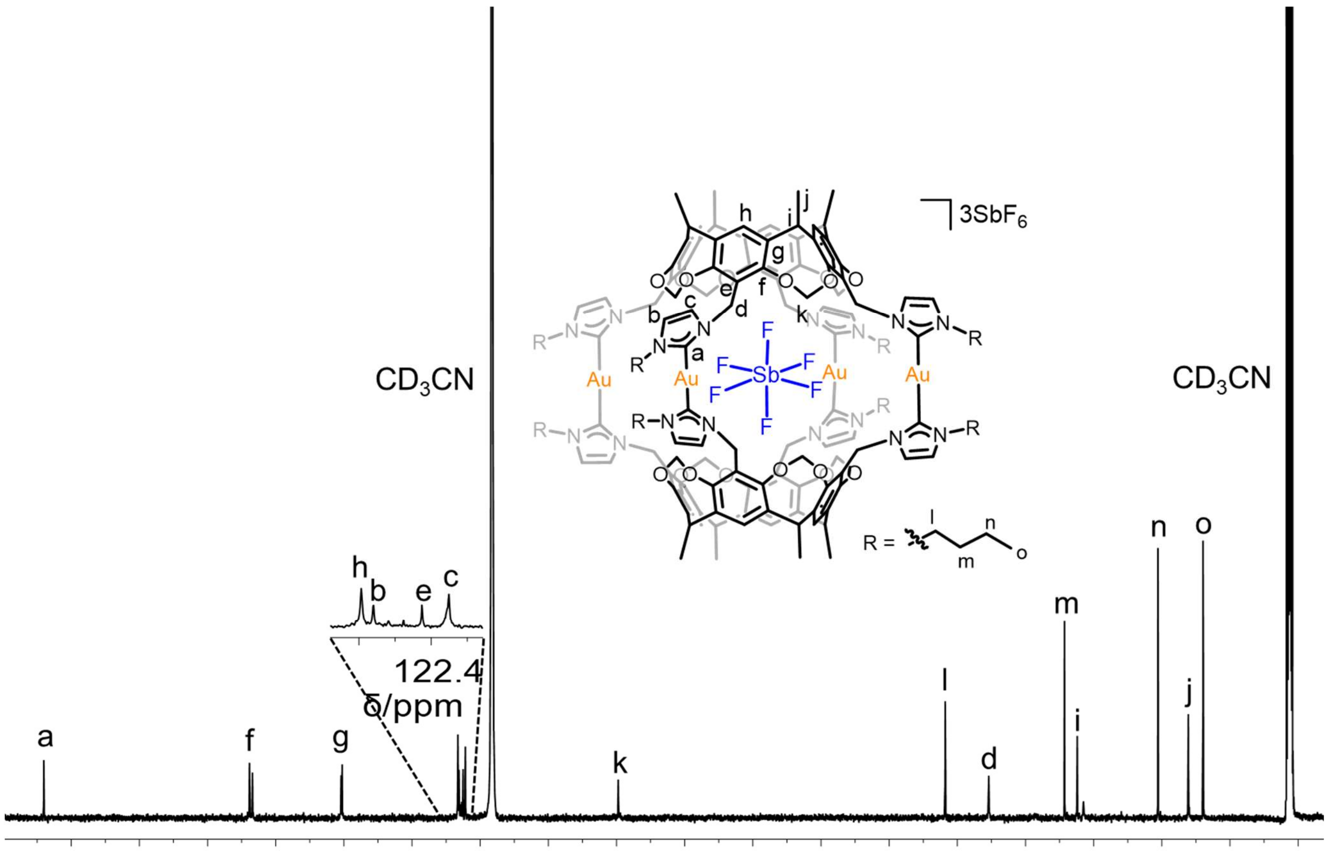

Figure S52. $^{13}C\{^{1}H\}$ NMR spectrum (150 MHz, CD_3CN) of $[(SbF_6)\subset Au_4(\mathbf{1})_2](SbF_6)_3$.

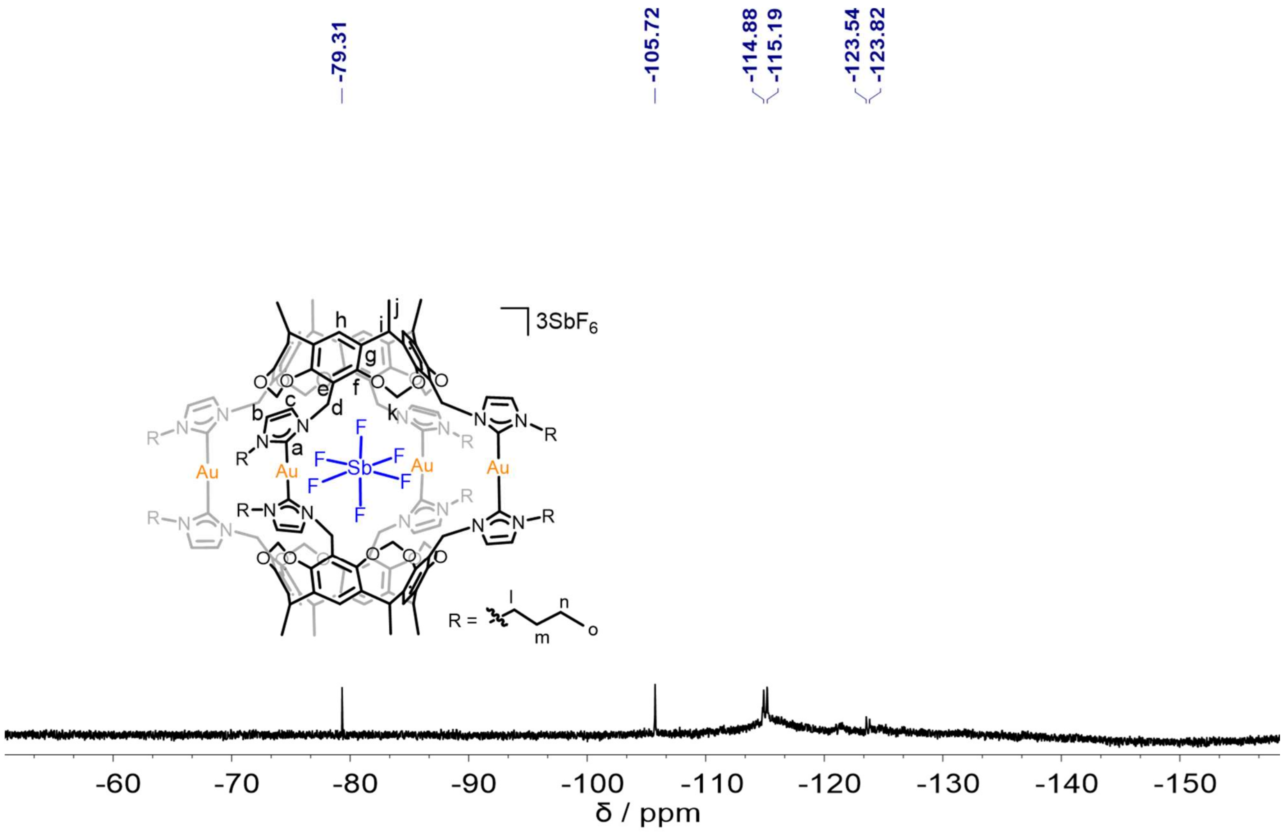

Figure S53. ^{19}F NMR spectrum (376 MHz, CD_3CN) of $[(SbF_6)\subset Au_4(\mathbf{1})_2](SbF_6)_3$.

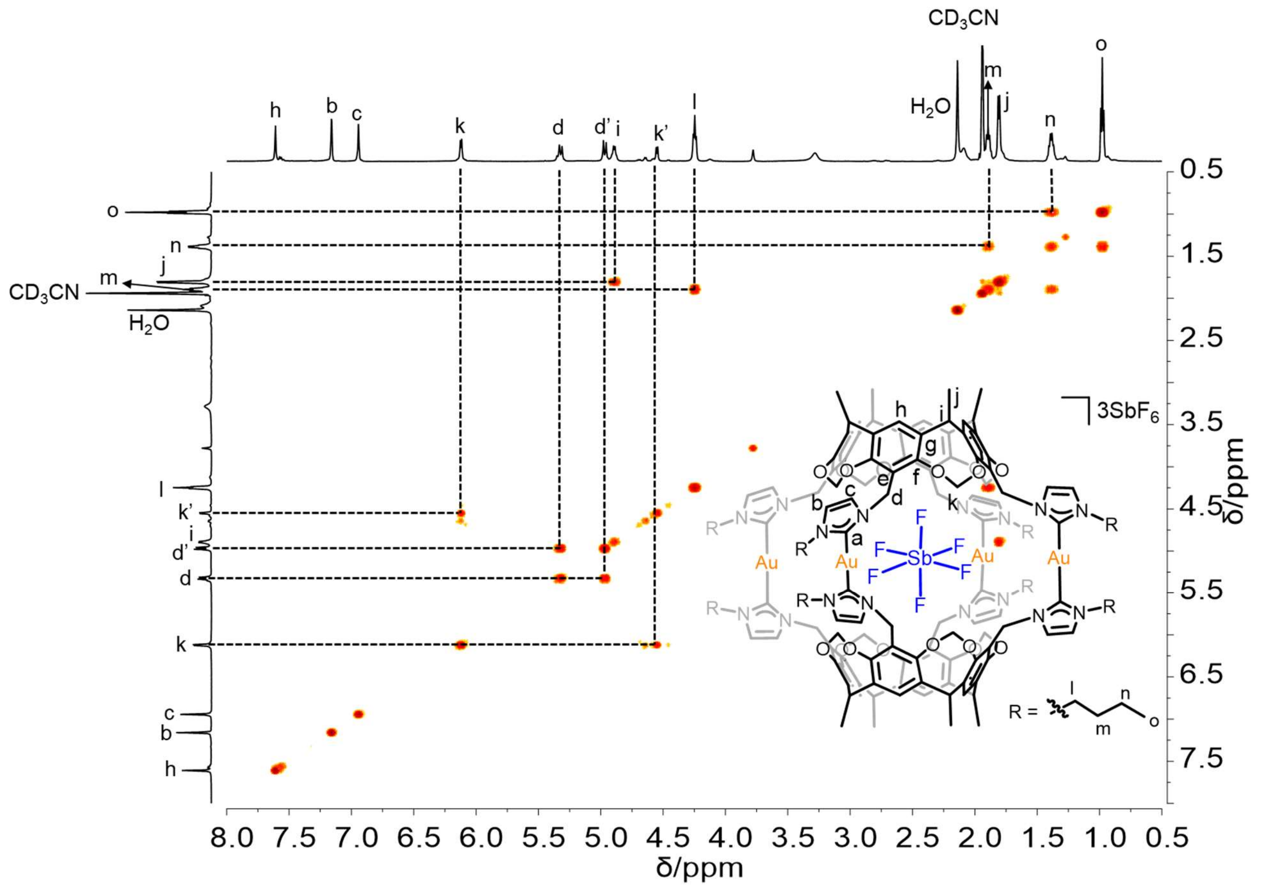

Figure S54. ^{1}H-^{1}H COSY spectrum (600 MHz, CD_3CN) of $[(SbF_6)\subset Au_4(\mathbf{1})_2](SbF_6)_3$.

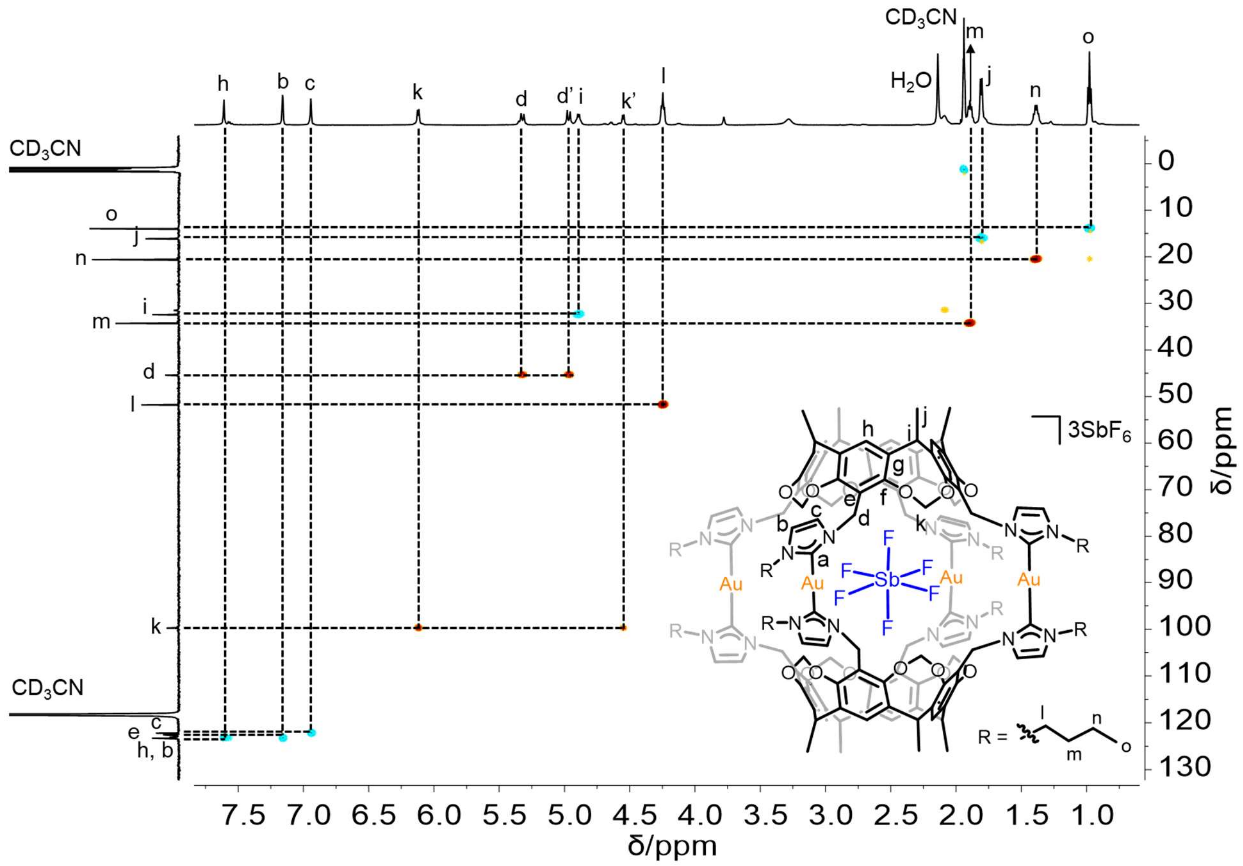

Figure S55. ^{1}H-^{13}C HSQC spectrum (600 MHz, CD$_3$CN) of [(SbF$_6$)⊂Au$_4$(**1**)$_2$](SbF$_6$)$_3$.

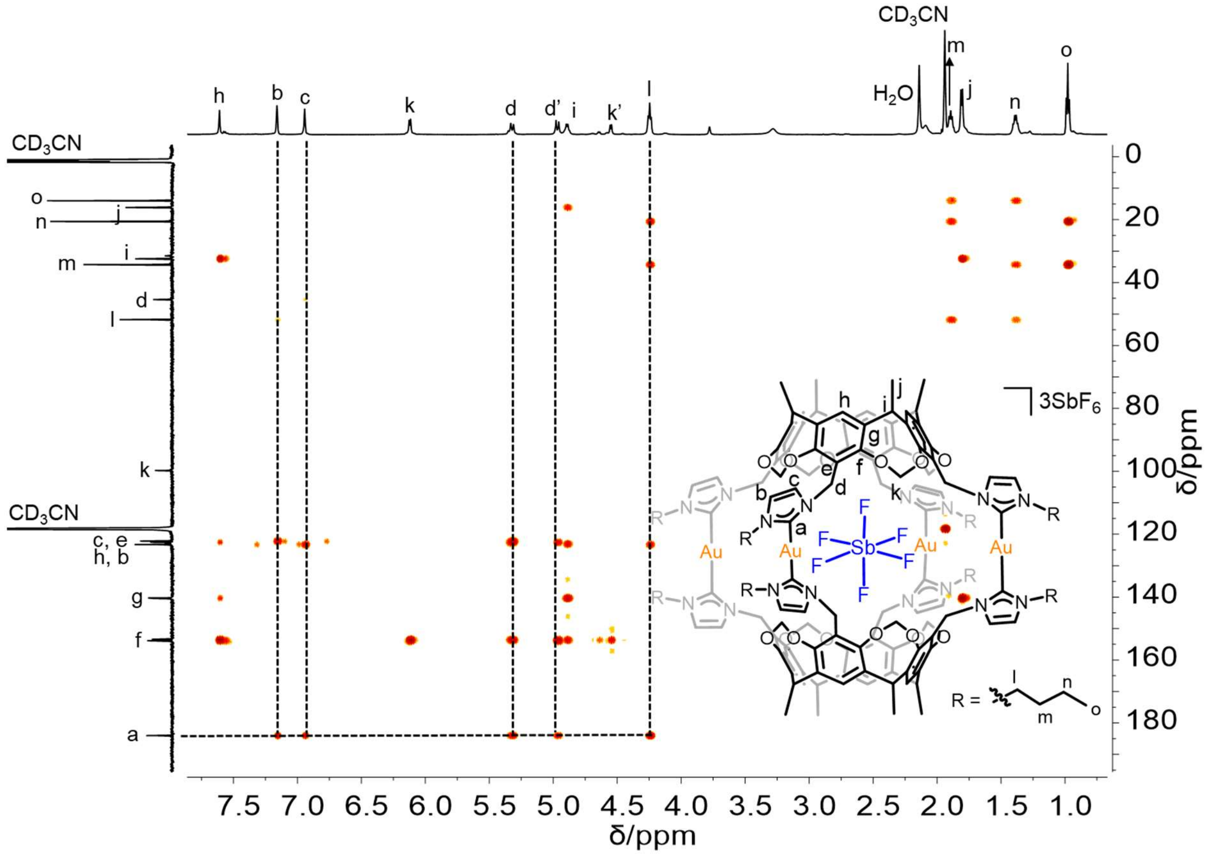

Figure S56. ^{1}H-^{13}C HMBC spectrum (600 MHz, CD$_3$CN) of [(SbF$_6$)⊂Au$_4$(**1**)$_2$](SbF$_6$)$_3$.

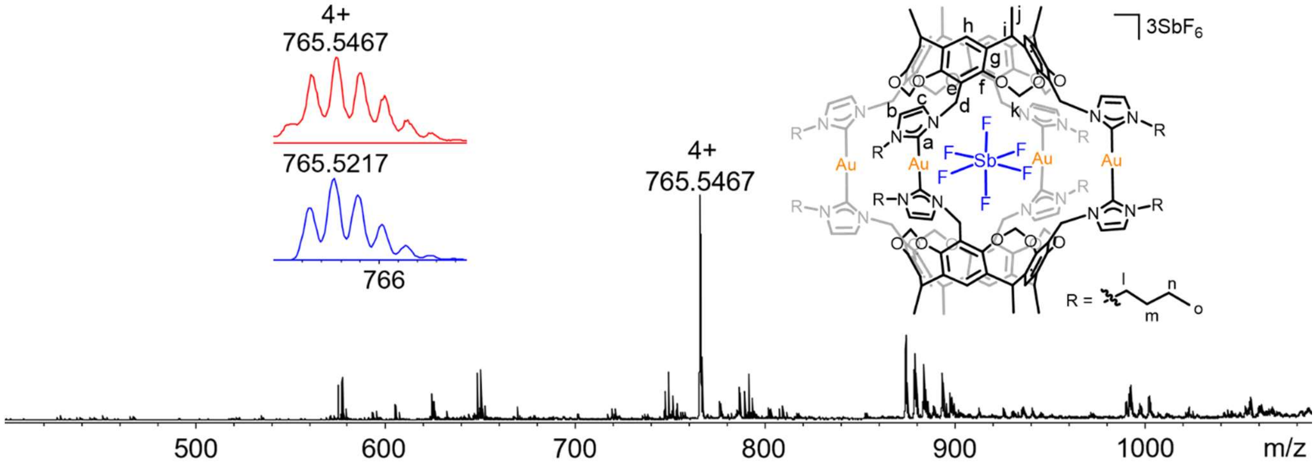

Figure S57. ESI-TOF mass spectrum of $[(SbF_6)\subset Au_4(\mathbf{1})_2](SbF_6)_3$ with isotope distribution for the 4+ cation peak (experimental in red, calculated in blue).

6. X-ray crystallography

Single crystals of compounds H_4-**1**$(BF_4)_4·2CH_3CN$, $[(CF_3SO_3)_8⊃Ag_{12}($**1**$)_6](CF_3SO_3)_4·54CH_3CN$, $[(SbF_6)⊃Ag_4($**1**$)_2](SbF_6)_3·2CH_3CN$, $[Au_4($**1**$)_2](BF_4)_4·11CH_3CN$ and $[(SbF_6)⊃Au_4($**1**$)_2](SbF_6)_3$ $·5CH_3CN·H_2O$ were grown by slow diffusion of diethyl ether into a saturated acetonitrile solutions of the complexes at ambient temperature. Single crystal of $[\{Ag(CH_3CN)_4(BF_4)_8\}⊃Ag_{12}($**1**$)_6](BF_4)_5·88CH_3CN$ suitable for X-ray diffraction analysis have been obtained by slowly diffusing diisopropyl ether into a saturated acetonitrile solution of the compound. Single crystal X-ray diffraction data for H_4-**1**$(BF_4)_4·2CH_3CN$, $[(CF_3SO_3)_8⊃Ag_{12}($**1**$)_6](CF_3SO_3)_4·54CH_3CN$, $[Au_4($**1**$)_2](BF_4)·11CH_3CN$ and $[(SbF_6)⊃Au_4($**1**$)_2](SbF_6)_3$ $·5CH_3CN·H_2O$ were collected with a Bruker D8 Venture diffractometer. Single-crystal X-ray diffraction data for $[\{Ag(CH_3CN)_4(BF_4)_8\}⊃Ag_{12}($**1**$)_6](BF_4)_5·88CH_3CN$ and $[(SbF_6)⊃Ag_4($**1**$)_2](SbF_6)_3$ $·2CH_3CN$ were collected at the BL17B macromolecular crystallography beamline of the National Facility for Protein Science at the Shanghai Synchrotron Radiation Facility (SSRF) at 153 K.

Structure solutions were found with the Olex 2 program using charge flipping and were refined with the SHELXL refinement package using Least Squares minimisation.[4–6] A number of disordered solvent molecules could not be restrained properly and were therefore removed using the SQUEEZE routine. While the introduction of *n*-butyl groups at the NHC donors increased the solubility of the complexes, it also causes a certain amount of disorder due to thermal motion. Some restrictions (SIMU, DELU, ISOR, SIMU, DFIX) were used to restrain anions, solvents and ligands for the refinements. The structure graphics shown were generated using the program Diamond. For selected metric parameters of the individual complexes see Tables S1. All crystal and data collection details are summarized in Tables S2–S7.

Table S1. Listing of the range of bond distances [Å] and bond angles [°] in compounds $[\{Ag(CH_3CN)_4(BF_4)_8\}⊃Ag_{12}($**1**$)_6](BF_4)_5·88CH_3CN$, $[(CF_3SO_3)_8⊃Ag_{12}($**1**$)_6](CF_3SO_3)_4·54CH_3CN$, $[(SbF_6)⊃Ag_4($**1**$)_2](SbF_6)_3·2CH_3CN$, $[Au_4($**1**$)_2](BF_4)_4·11CH_3CN$ and $[(SbF_6)⊃Au_4($**1**$)_2](SbF_6)_3$ $·5CH_3CN·H_2O$

Compound	bond lengths C_{NHC}–M [Å]	angles C_{NHC}–M–C_{NHC} [°]
$[\{Ag(CH_3CN)_4(BF_4)_8\}⊃Ag_{12}($**1**$)_6](BF_4)_5·88CH_3CN$	2.051(7)–2.064(10)	171.0(3)–174.2(4)
$[(CF_3SO_3)_8⊃Ag_{12}($**1**$)_6](CF_3SO_3)_4·54CH_3CN$	1.903(15)–2.20(2)	169.1(7)–175.7(8)
$[(SbF_6)⊃Ag_4($**1**$)_2](SbF_6)_3·2CH_3CN$	2.036(12)–2.120(10)	175.6(4)–177.3(4)
$[Au_4($**1**$)_2](BF_4)_4·11CH_3CN$	1.88(3)–2.17(3)	162.9(12)–178.7(6)
$[(SbF_6)⊃Au_4($**1**$)_2](SbF_6)_3·5CH_3CN·H_2O$	2.007(12)–2.029(11)	178.0(5)–179.3(5)

Table S2. Crystal and refinement data for H_4-**1**$(BF_4)_4·2CH_3CN$

Empirical formula	$C_{72}H_{90}N_{10}B_4F_{16}O_8$
Formula weight	1570.77
Temperature/K	248
Crystal system	monoclinic
Space group	$P2_1/c$
a/Å	15.648(11)
b/Å	17.271(10)
c/Å	32.33(2)
α/°	90
β/°	94.02(3)
γ/°	90
Volume/Å^3	8717(10)
Z	4
ρ_{calc} (g·cm^{-3})	1.197
μ (mm^{-1})	0.101
F(000)	3280
Crystal size (mm^3)	0.2 × 0.2 × 0.1
Radiation	Mo$K\alpha$ (λ = 0.71073)
2Θ range for data collection/°	4.22 to 50.81
Index ranges	$-18 \leq h \leq 18$, $-18 \leq k \leq 20$, $-38 \leq l \leq 38$
Reflections collected	160948
Independent reflections	15973 [R_{int} = 0.0729]
Data/restraints/parameters	15973/191/1029
Goodness-of-fit on F^2	1.048
Final R indexes [I> = 2σ (I)]	R_1 = 0.1046, wR_2 = 0.3171
Final R indexes [all data]	R_1 = 0.1361, wR_2 = 0.3457
Largest diff. peak/hole (e·Å^{-3})	0.70/–0.64
CCDC	2373367

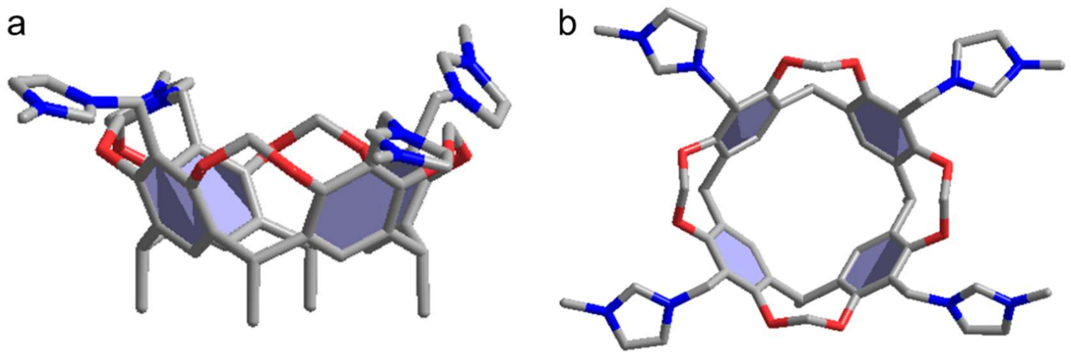

Figure S58. Side view (a) and top view (b) of the molecular structure of cation $[H_4\text{-}\mathbf{1}]^{4+}$ in $H_4\text{-}\mathbf{1}(BF_4)_4 \cdot 2CH_3CN$. Hydrogen atoms have been omitted for clarity.

Table S3. Crystal and refinement data for $[\{Ag(CH_3CN)_4(BF_4)_8\}\supset Ag_{12}(\mathbf{1})_6](BF_4)_5\cdot 88CH_3CN$

Empirical formula	$C_{592}H_{756}N_{140}Ag_{13}B_{13}F_{52}O_{48}$
Formula weight	13132.16
Temperature/K	153(2)
Crystal system	cubic
Space group	$Pn\bar{3}n$
a/Å	49.240(12)
b/Å	49.240(12)
c/Å	49.240(12)
α/°	90
β/°	90
γ/°	90
Volume/Å^3	119386(87)
Z	6
ρ_{calc} (g·cm^{-3})	1.096
μ (mm^{-1})	0.355
F(000)	40896
Crystal size (mm^3)	0.15× 0.12× 0.08
Radiation	synchrotron (λ = 0.68883)
2Θ range for data collection/°	2.78 to 51.0
Index ranges	$-61 \le h \le 61, -61 \le k \le 61, -61 \le l \le$ [illegible]
Reflections collected	1278230
Independent reflections	19991 [R_{int} = 0.1038]
Data/restraints/parameters	19991/864/739
Goodness-of-fit on F^2	1.069
Final R indexes [I> = 2σ (I)]	R_1 = 0.1006, wR_2 = 0.1883
Final R indexes [all data]	R_1 = 0.1405, wR_2 = 0.1983
Largest diff. peak/hole (e·Å^{-3})	0.63/–0.72
CCDC	2373432

Table S4. Crystal and refinement data for $[(CF_3SO_3)_8\supset Ag_{12}(\mathbf{1})_6](CF_3SO_3)_4\cdot 54CH_3CN$

Empirical formula	$C_{528}H_{642}N_{102}Ag_{12}F_{36}O_{84}S_{12}$
Formula weight	12124.56
Temperature/K	150(2)
Crystal system	triclinic
Space group	*P*–1
a/Å	26.221(13)
b/Å	32.57(2)
c/Å	40.079(13)
α/°	82.61(3)
β/°	79.475(14)
γ/°	68.23(2)
Volume/Å^3	31179(29)
Z	2
ρ_{calc} (g·cm^{-3})	1.291
μ (mm^{-1})	2.670
F(000)	12552
Crystal size (mm^3)	0.24× 0.22× 0.18
Radiation	Ga Kα (λ = 1.34139)
2Θ range for data collection/°	4.67 to 102.66
Index ranges	–30 ≤ h ≤ 27, –37 ≤ k ≤ 37, –46 ≤ l ≤ 46
Reflections collected	271453
Independent reflections	101851 [R_{int} = 0.0953]
Data/restraints/parameters	101851/7533/5482
Goodness-of-fit on F^2	1.047
Final *R* indexes [I> = 2σ (I)]	R_1 = 0.1510, wR_2 = 0.3127
Final *R* indexes [all data]	R_1 = 0.2489, wR_2 = 0.3614
Largest diff. peak/hole (e·Å^{-3})	1.64/–1.23
CCDC	2472685

Table S5. Crystal and refinement data for $[(SbF_6)\supset Ag_4(\mathbf{1})_2](SbF_6)_3\cdot 2CH_3CN$

Empirical formula	$C_{140}H_{166}N_{18}Ag_4F_{24}O_{16}Sb_4$
Formula weight	3731.38
Temperature/K	153(2)
Crystal system	monoclinic
Space group	*C*2/*c*
a/Å	25.240(16)
b/Å	21.050(2)
c/Å	30.050(3)
α/°	90
β/°	107.230(2)
γ/°	90
Volume/Å^3	15249(2)
Z	4
ρ_{calc} (g·cm^{-3})	1.625
μ (mm^{-1})	1.199
F(000)	7472
Crystal size (mm^3)	0.3× 0.1× 0.1
Radiation	synchrotron (λ = 0.68883)
2Θ range for data collection/°	2.75 to 52.0
Index ranges	–31 ≤ h ≤ 32, –26 ≤ k ≤ 26, –38 ≤ l ≤ 38
Reflections collected	101263
Independent reflections	15423 [R_{int} = 0.0930]
Data/restraints/parameters	15423/150/1031
Goodness-of-fit on F^2	1.052
Final *R* indexes [I> = 2σ (I)]	R_1 = 0.0813, wR_2 = 0.2376
Final *R* indexes [all data]	R_1 = 0.1302, wR_2 = 0.2754
Largest diff. peak/hole (e·Å^{-3})	2.31/–1.63
CCDC	2373431

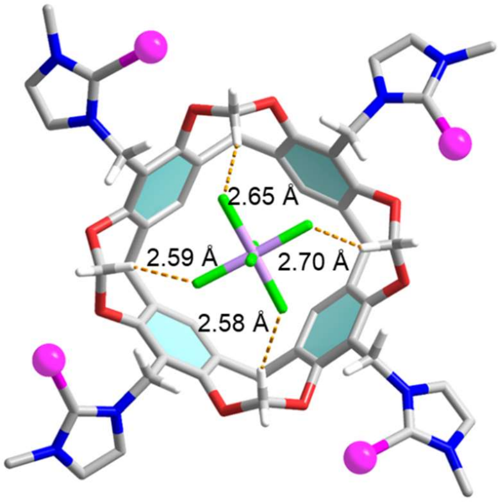

Figure S59. Sb–F···H hydrogen bonds to one of the tetracarbene ligands in the cation $[(SbF_6)\supset Ag_4(\mathbf{1})_2]^{3+}$ of assembly $[(SbF_6)\supset Ag_4(\mathbf{1})_2](SbF_6)_3{\cdot}2CH_3CN$. Due to the crystallographic twofold axis, an identical set of hydrogen bonds exist to the second tetracarbene ligand).

Table S6. Crystal and refinement data for $[Au_4(\mathbf{1})_2](BF_4)_4{\cdot}11CH_3CN$

Empirical formula	$C_{158}H_{193}N_{27}Au_4B_4F_{16}O_{16}$
Formula weight	3861.49
Temperature/K	210
Crystal system	orthorhombic
Space group	*Fddd*
a/Å	22.5573(12)
b/Å	42.419(3)
c/Å	67.100(4)
α/°	90
β/°	90
γ/°	90
Volume/Å^3	64205(7)
Z	16
ρ_{calc} (g·cm^{-3})	1.598
μ (mm^{-1})	3.734
F(000)	31008
Crystal size/mm^3	0.2 × 0.17 × 0.15
Radiation	Mo$K\alpha$ (λ = 0.71073)
2Θ range for data collection/°	3.81 to 50.75
Index ranges	$-27 \le h \le 25$, $-41 \le k \le 51$, $-50 \le l \le 80$
Reflections collected	48711
Independent reflections	14624 [R_{int} = 0.0905]
Data/restraints/parameters	14624/1420/944
Goodness-of-fit on F^2	1.067
Final R indexes [I> = 2σ (I)]	R_1 = 0.0701, wR_2 = 0.1330
Final R indexes [all data]	R_1 = 0.1721, wR_2 = 0.1583
Largest diff. peak/hole (e·Å^{-3})	1.55/–0.98
CCDC	2482489

Table S7. Crystal and refinement data for $[(SbF_6)\supset Au_4(\mathbf{1})_2](SbF_6)_3\cdot 5CH_3CN\cdot H_2O$

Empirical formula	$C_{146}H_{177}N_{21}Au_4F_{24}O_{17}Sb_4$
Formula weight	4228.94
Temperature/K	224
Crystal system	monoclinic
Space group	$C2/c$
a/Å	25.3621(8)
b/Å	21.3081(7)
c/Å	30.0801(9)
α/°	90
β/°	107.2090(10)
γ/°	90
Volume/Å^3	15528.1(8)
Z	4
ρ_{calc} (g·cm^{-3})	1.809
μ (mm^{-1})	4.546
F(000)	8288
Crystal size/mm^3	0.2 × 0.18 × 0.16
Radiation	Mo$K\alpha$ (λ = 0.71073)
2Θ range for data collection/°	3.42 to 49.43
Index ranges	$-29 \leq h \leq 29$, $-25 \leq k \leq 25$, $-35 \leq l \leq 35$
Reflections collected	138630
Independent reflections	13184 [R_{int} = 0.0348]
Data/restraints/parameters	13184/3211/1031
Goodness-of-fit on F^2	1.057
Final R indexes [I> = 2σ (I)]	R_1 = 0.0606, wR_2 = 0.1226
Final R indexes [all data]	R_1 = 0.0721, wR_2 = 0.1285
Largest diff. peak/hole (e·Å^{-3})	1.83/–2.20
CCDC	2373403

7. Calculation of the hydrodynamic radii and the cavity volume for selected capsules

The hydrodynamic radii were calculated from the diffusion coefficients obtained by DOSY NMR spectroscopy of the nanocapsules. The results are in good agreement with the radii calculated from the single crystal X-ray diffraction measurements. As expected, the two nanosized dodecanuclear nanocapsules were found to have a significantly larger hydrodynamic radius than the tetranuclear capsules.

Structural size simulation from ^{1}H DOSY NMR spectroscopy for compounds $[\{Ag(CH_3CN)_4(BF_4)_8\}\subset Ag_{12}(\mathbf{1})_6](BF_4)_5$, $[(OTf)_8\subset Ag_{12}(\mathbf{1})_6](OTf)_4$ and $[(SbF_6)\subset Ag_4(\mathbf{1})_2](SbF_6)_3$.

According to the Stokes-Einstein equation, the diffusion coefficients (D) can be calculated based on the hydrodynamic radius (r_h) for a sphere-shaped diffusing entity:

$$D = \frac{k_B T}{6\pi\eta r_h} \tag{1}$$

where "k_B" is the Boltzmann constant, "T" is the experimental temperature, and "η" is the viscosity of acetonitrile-d_3.

As for a sphere-shaped entity (compounds $[\{Ag(CH_3CN)_4(BF_4)_8\}\subset Ag_{12}(\mathbf{1})_6](BF_4)_5$, $[(OTf)_8\subset Ag_{12}(\mathbf{1})_6](OTf)_4$ and $[(SbF_6)\subset Ag_4(\mathbf{1})_2](SbF_6)_3$), equation 1 can be rewritten as equation 2 to obtain the hydrodynamic radius r_h:

$$r_h = \frac{k_B T}{6\pi\eta D} \tag{2}$$

For $[(OTf)_8 \subset Ag_{12}(\mathbf{1})_6](OTf)_4$, the diffusion coefficient is $D = 8.46 \times 10^{-10}$ m^2s^{-1} in CD_3CN (Figure 1c). The calculated simulated radius based on a sphere-shaped model and the radius calculated from X-ray diffraction data are shown below:

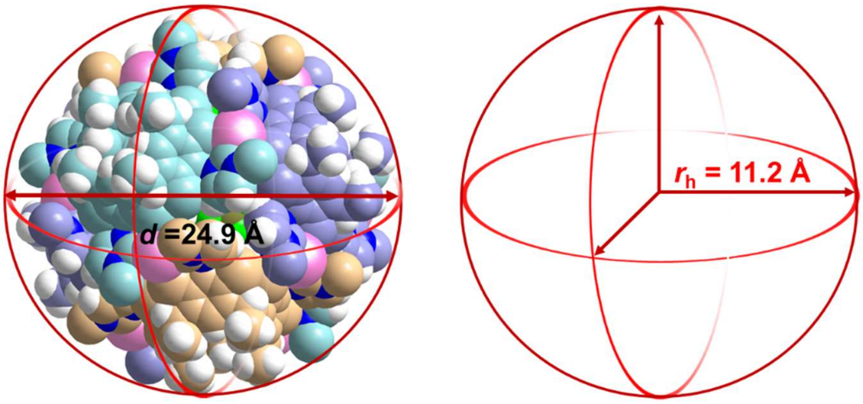

radius determined by X-ray diffraction

radius calculated from the diffusion coefficient

k_B(J/K)	1.38E-23	
T(K)	324	
η (Pa S, CH_3CN, 324 K)	2.45E-04	
D(m^2/s)	8.46E-10	π = 3.1415926
r_h(m)	1.12E-09	
r_h (Å)	11.2	

Figure S60. Microsoft Excel spreadsheet used to fit $[(OTf)_8 \subset Ag_{12}(\mathbf{1})_6](OTf)_4$ in a CD_3CN solution using the sphere-shaped model.

For $[\{Ag(CH_3CN)_4(BF_4)_8\} \subset Ag_{12}(\mathbf{1})_6](BF_4)_5$ the diffusion coefficient is $D = 8.91 \times 10^{-10}$ m^2s^{-1} in CD_3CN (Figure S17). The simulated radius based on a sphere-shaped model is calculated as shown below:

k_B(J/K)	1.38E-23	
T(K)	324	
η (Pa S, CH_3CN, 324 K)	2.45E-04	
D(m^2/s)	8.91E-10	π = 3.1415926
r_h(m)	1.09E-09	
r_h (Å)	10.9	

Figure S61. Microsoft Excel spreadsheet used to fit $[\{Ag(CH_3CN)_4(BF_4)_8\} \subset Ag_{12}(\mathbf{1})_6](BF_4)_5$ in a CD_3CN solution using the sphere-shaped model.

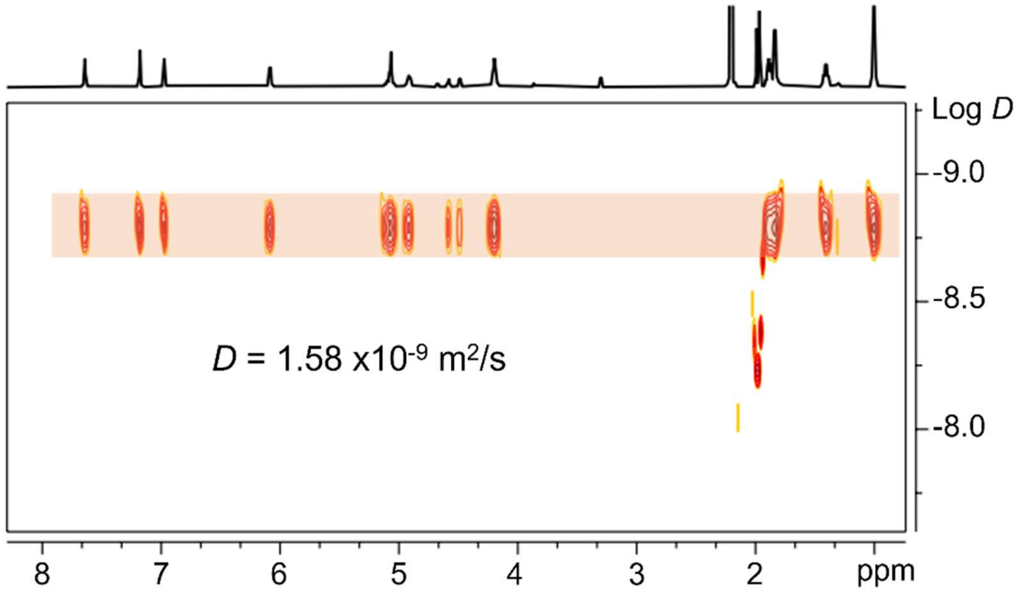

Figure S62. ^{1}H DOSY spectrum (400 MHz, CD_3CN) of $[(SbF_6)\subset Ag_4(\mathbf{1})_2](SbF_6)_3$.

For $[(SbF_6)\subset Ag_4(\mathbf{1})_2](SbF_6)_3$, the diffusion coefficient is $D = 1.58 \times 10^{-9}$ m^2s^{-1} in CD_3CN (Figure S62). The calculated simulated radius based on a sphere-shaped model and the radius calculated from X-ray diffraction data are shown below:

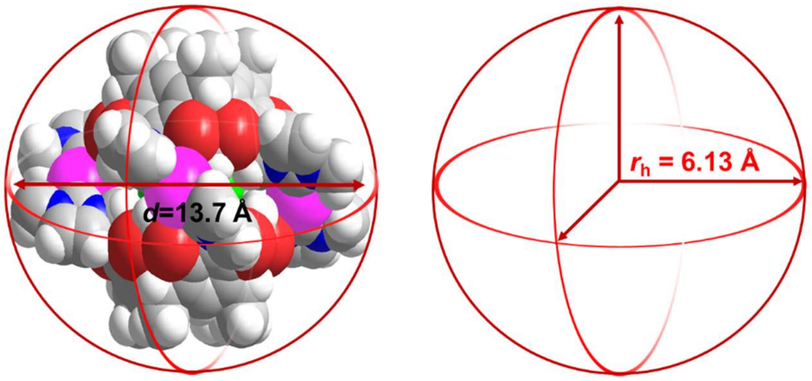

k_B(J/K)	1.38E-23	
T(K)	324	
η (Pa S, CH_3CN, 324 K)	2.45E-04	
$D(m^2/s)$	1.58E-09	π = 3.1415926
r_h(m)	6.13E-10	
r_h (Å)	6.13	

Figure S63. Microsoft Excel spreadsheet used to fit $[(SbF_6)\subset Ag_4(\mathbf{1})_2](SbF_6)_3$ in a CD_3CN solution using the sphere-shaped model.

The internal cavity volumes of the two types of nanocapsules were calculated using VOIDOO. The VOIDOO calculations are based on the X-ray structure data of $[\{Ag(CH_3CN)_4(BF_4)_8\}\supset Ag_{12}(\mathbf{1})_6](BF_4)_5\cdot 88CH_3CN$, $[(CF_3SO_3)_8\supset Ag_{12}(\mathbf{1})_6](CF_3SO_3)_4\cdot 54CH_3CN$ and $[(SbF_6)\supset Ag_4(\mathbf{1})_2](SbF_6)_3\cdot 2CH_3CN$. A virtual probe with the minimum radius selected that would not extend beyond the cavity of the largest structure was employed. The cavity volume of cation $[Ag_{12}(\mathbf{1})_6]^{12+}$ in $[\{Ag(CH_3CN)_4(BF_4)_8\}\supset Ag_{12}(\mathbf{1})_6](BF_4)_5$ was estimated by using the VOIDOO program and a 3.0 Å probe radius to measure 1614.6 Å^3 (Figure S64). The cavity volume of cation $[Ag_{12}(\mathbf{1})_6]^{12+}$ in $[(CF_3SO_3)_8\supset Ag_{12}(\mathbf{1})_6](CF_3SO_3)_4$ was estimated with a 3.0 Å probe radius at 1674.8 Å^3 (Figure S65) and the cavity volume of $[Ag_4(\mathbf{1})_2]^{4+}$ in $[(SbF_6)\supset Ag_4(\mathbf{1})_2](SbF_6)_3$ measured 204.9 Å^3 (probe radius 1.3, Figure S66).

Primary grid spacing: 0.100

Maximum number of volume-refinement cycles: 30

Minium size of secondary grid: 3

Grid for plot files: 0.100

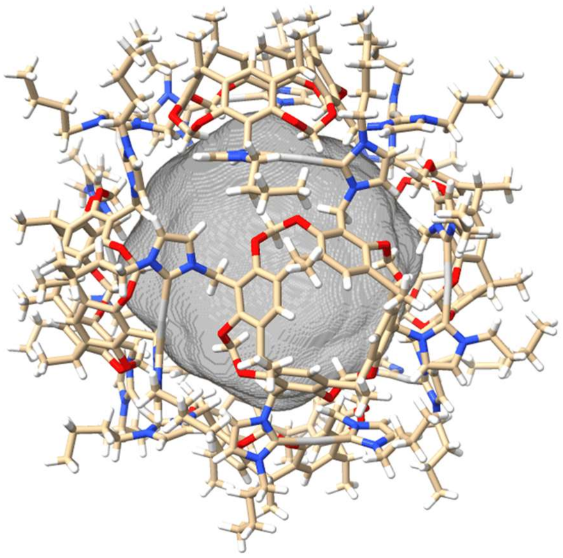

Figure S64. Cationic part of the molecular structure of $[(CF_3SO_3)_8\supset Ag_{12}(\mathbf{1})_6](CF_3SO_3)_4$ as determined by single crystal X-ray diffraction showing the internal cavity volume calculated using VOIDOO.

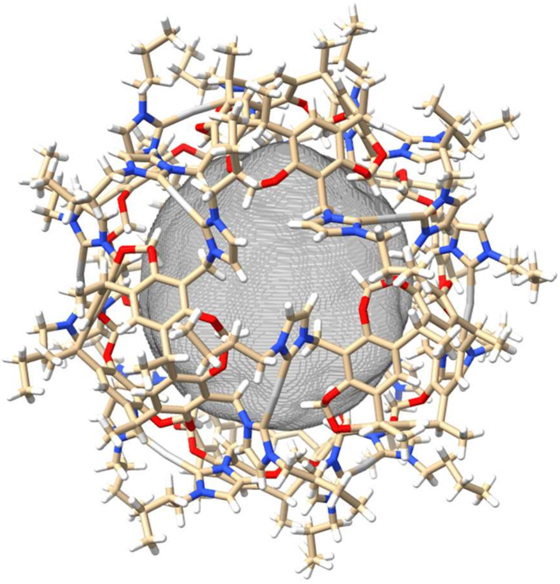

Probe radius: 3.0 V = 1614.6 Å^3

Figure S65. Cationic part of the molecular structure of $[\{Ag(CH_3CN)_4(BF_4)_8\}\supset Ag_{12}(\mathbf{1})_6](BF_4)_5$ as determined by single crystal X-ray diffraction showing the internal cavity volume calculated using VOIDOO.

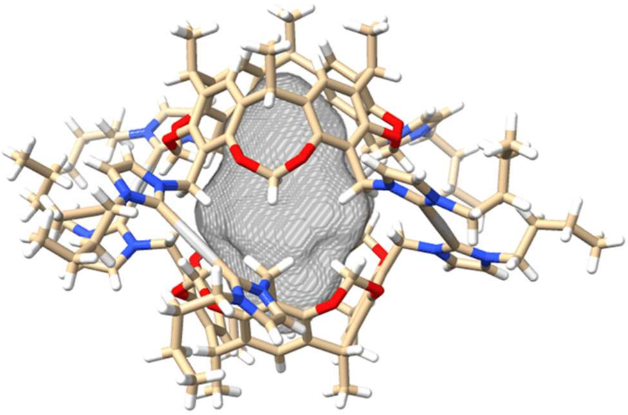

Probe radius: 1.3 V = 204.9 Å^3

Figure S66. Cationic part of the molecular structure of $[(SbF_6)\supset Ag_4(\mathbf{1})_2](SbF_6)_3$ as determined by single crystal X-ray diffraction showing the internal cavity volume calculated using VOIDOO.

7. References

[1] L. M. Tunstad, J. A. Tucker, E. Dalcanale, J. Weiser, J. A. Bryant, J. C. Sherman, R. C. Helgeson, C. B. Knobler, D. J. Cram., *J. Org. Chem.* **1989**, *54*, 1305–1312.

[2] a) H. Boerrigter, W. Verboom, D. N. Reinhoudt, *J. Org. Chem.* **1997**, *62*, 7148–7155; b) S. A. Moussaoui, Z. Damaj, M. Wehbie, S. P. Rostaing, I. Karamé, *Int. J. Org. Chem.* **2017**, *4*, 403–411.

[3] Y.-J. Hu, J. Yang, Y.-Y. Liu, S. Song, J.-F. Ma, *Cryst. Growth Des.* **2015**, *15*, 3822–3831.

[4] O. V. Dolomanov, L. J. Bourhis, R. J. Gildea, J. A. K. Howard, H. Puschmann, *J. Appl. Cryst.* **2009**, *42*, 339–341.

[5] L. J. Bourhis, O. V. Dolomanov, R. J. Gildea, J. A. K. Howard, H. Puschmann, *Acta Crystallogr.* **2015**, *A71*, 59–75.

[6] G. M. Sheldrick, *Acta Crystallogr.* **2015**, *C71*, 3–8.
